# A catalog of validity indices for step counting wearable technologies during treadmill walking: the CADENCE-adults study

**DOI:** 10.1186/s12966-022-01350-9

**Published:** 2022-09-08

**Authors:** Jose Mora-Gonzalez, Zachary R. Gould, Christopher C. Moore, Elroy J. Aguiar, Scott W. Ducharme, John M. Schuna, Tiago V. Barreira, John Staudenmayer, Cayla R. McAvoy, Mariya Boikova, Taavy A. Miller, Catrine Tudor-Locke

**Affiliations:** 1 PROFITH “PROmoting FITness and Health Through Physical Activity” Research Group, Department of Physical Education and Sports, Faculty of Sport Sciences, Sport and Health University Research Institute (iMUDS), University of Granada, Granada, Spain; 2 College of Health and Human Services, University of North Carolina at Charlotte, 9201 University City Blvd, Charlotte, NC 28223 USA; 3 Department of Kinesiology, University of Massachusetts Amherst, Amherst, MA USA; 4 Department of Epidemiology, University of North Carolina at Chapel Hill, Chapel Hill, NC USA; 5 Department of Kinesiology, The University of Alabama, Tuscaloosa, AL USA; 6 Department of Kinesiology, California State University, Long Beach, Long Beach, CA USA; 7 School of Biological and Population Health Sciences, Oregon State University, Corvallis, OR USA; 8 Exercise Science Department, Syracuse University, Syracuse, NY USA; 9 Department of Mathematics and Statistics, University of Massachusetts Amherst, Amherst, MA USA; 10 Hanger Institute for Clinical Research and Education, Austin, TX USA

**Keywords:** Accelerometer, Accuracy, Bias, Measurement, Pedometer, Physical activity

## Abstract

**Background:**

Standardized validation indices (i.e., accuracy, bias, and precision) provide a comprehensive comparison of step counting wearable technologies.

**Purpose:**

To expand a previously published child/youth catalog of validity indices to include adults (21–40, 41–60 and 61–85 years of age) assessed across a range of treadmill speeds (slow [0.8–3.2 km/h], normal [4.0–6.4 km/h], fast [7.2–8.0 km/h]) and device wear locations (ankle, thigh, waist, and wrist).

**Methods:**

Two hundred fifty-eight adults (52.5 ± 18.7 years, 49.6% female) participated in this laboratory-based study and performed a series of 5-min treadmill bouts while wearing multiple devices; 21 devices in total were evaluated over the course of this multi-year cross-sectional study (2015–2019). The criterion measure was directly observed steps. Computed validity indices included accuracy (mean absolute percentage error, MAPE), bias (mean percentage error, MPE), and precision (correlation coefficient, *r*; standard deviation, SD; coefficient of variation, CoV).

**Results:**

Over the range of normal speeds, 15 devices (Actical, waist-worn ActiGraph GT9X, activPAL, Apple Watch Series 1, Fitbit Ionic, Fitbit One, Fitbit Zip, Garmin vivoactive 3, Garmin vivofit 3, waist-worn GENEActiv, NL-1000, PiezoRx, Samsung Gear Fit2, Samsung Gear Fit2 Pro, and StepWatch) performed at < 5% MAPE. The wrist-worn ActiGraph GT9X displayed the worst accuracy across normal speeds (MAPE = 52%). On average, accuracy was compromised across slow walking speeds for all wearable technologies (MAPE = 40%) while all performed best across normal speeds (MAPE = 7%). When analyzing the data by wear locations, the ankle and thigh demonstrated the best accuracy (both MAPE = 1%), followed by the waist (3%) and the wrist (15%) across normal speeds. There were significant effects of speed, wear location, and age group on accuracy and bias (both *p* < 0.001) and precision (*p* ≤ 0.045).

**Conclusions:**

Standardized validation indices cataloged by speed, wear location, and age group across the adult lifespan facilitate selecting, evaluating, or comparing performance of step counting wearable technologies. Speed, wear location, and age displayed a significant effect on accuracy, bias, and precision. Overall, reduced performance was associated with very slow walking speeds (0.8 to 3.2 km/h). Ankle- and thigh-located devices logged the highest accuracy, while those located at the wrist reported the worst accuracy.

**Trial registration:**

Clinicaltrials.gov NCT02650258. Registered 24 December 2015.

**Supplementary Information:**

The online version contains supplementary material available at 10.1186/s12966-022-01350-9.

## Introduction

Wearable technologies continue to evolve and diversify, yet step counting remains a popular and essential feature of devices designed to assess, monitor, and modulate everyday physical activity behavior [1]. However, the varying mechanisms/decisions/rules/algorithms operationalized by these technologies can result in differential sensitivity and specificity to step measurement and ultimately produce dissimilar estimates of the directly observed ground criterion [2, 3]. As we have previously argued [4], standardized validation indices, specifically those related to accuracy, bias, and precision, are needed to establish device performance expectations and ensure comparability of outputs if step counting is to move beyond popular gadgetry and to be incorporated into clinical practice and public health guidelines.

As an initial step in this process, we conducted a scoping review [4] to identify studies evaluating wearable technologies using direct observation of step counting as a criterion standard during treadmill ambulation, itself an easily controllable and thus replicable validation method [5]. As part of the present study, we have updated the 2020 scoping review [4] and identified 28 additional articles in adults, of which validation indices related to accuracy, bias, and precision are reported in the Additional file 1. Out of the newly 28 identified studies, 7 reported some form of each of the validation indices assessing device performance in adults between ages of 18 to 87 years. Slightly more than a third (39% [*n* = 11]) of the studies reported analyses of mean absolute percent error (MAPE) as an index of accuracy. The smallest values, indicating higher accuracy, were observed during faster speeds by ankle-worn devices. Decreased device performance was recognized at slower speeds with wrist-worn devices. And as we have previously reported [4], the performance of consumer and research wearable technologies are affected by speed, wear location, and step counting algorithms/settings. Despite the identification of additional studies, the literature remains fractured and inconsistently presented. It is clear that this area of research requires a more comprehensive effort to fully catalog a broader array of standardized validity indices representing concurrently worn wearable technologies while accounting for acknowledged threats to validity, including speed, wear location, and age.

The purpose of this secondary analysis of the CADENCE-Adults’ data set [6–8] is to build upon a previously published catalog based on the results of the CADENCE-Kids study that focused on data collected in 6–20 year old children/youth [9]. Specifically, herein we computed accuracy (MAPE), bias (mean percent error, MPE), and precision (standard deviation, SD; coefficient of variation, CoV; and correlation coefficient, *r*) validity indices related to treadmill speed and wear location of 21 different wearable technologies tested in individuals across the adult lifespan (i.e., 21–85 years of age). Together, these concatenating works and ultimately comprehensive catalog forms the basis for simplifying performance evaluation of step counting wearable technologies and easing comparison of device-derived data.

## Methods

### Study design and regulatory information

CADENCE-Adults was a multi-year laboratory-based, cross-sectional study registered with ClinicalTrials.gov (NCT02650258) and designed to determine cadence (steps/min) thresholds associated with physical activity intensity across the adult lifespan (i.e., 21–85 years of age) [6–8]. Data collection took place at the University of Massachusetts Amherst in three phases: January to October 2016 for 21–40 year olds [8], January to October 2017 for 41–60 year olds [7], November 2018 to August 2019 for 61–85 year olds [6].

CADENCE-Adults was approved by the University of Massachusetts Amherst Institutional Review Board. After first phone screening to identify eligible participants, we scheduled an in-person screening evaluation where participants provided signed informed consent prior to beginning with data collection.

### Participants

A sex- and age-balanced sample of 10 men and 10 women for each 5-year age category between 21–85 years (i.e., 21–25, 26–30, 31–35 years of age, …) was recruited [6–8], and a final sample of 260 individuals participated. This recruitment strategy was carried out with the aim of favoring minimization of sources of bias, improving generalizability of findings and ensuring an equal distribution of participants across the lifespan age range of this study. Recruitment strategies included newspaper and radio advertisements, e-mails, electronic postings, flyers, general recruitment events (i.e., retirement villages, assisted living centers, and community centers), and word-of-mouth. Interested individuals contacted us via telephone or email. We then phone screened them to determine eligibility based on our inclusion/exclusion criteria. Potential participants were re-screened again to confirm eligibility during an in-person visit before obtaining informed consent and prior to beginning any data collection. Exclusion criteria included: use of wheelchairs, walking aids or any impairment for normal ambulation; mental illness hospitalization in the 5 years previous to the data collection; pregnancy; current tobacco use; a stroke or any other cardiovascular disease; a body mass index (BMI) indicating underweight or severe obesity (BMI < 18.5 kg/m^2^ or > 40 kg/m^2^); stage 2 hypertension (≥ 160 mmHg systolic blood pressure or ≥ 100 mmHg diastolic blood pressure); use of pacemaker or similar implanted medical device; or any condition and/or use of medication that could alter physiological response to exercise. Our medical investigator reviewed a resting electrocardiogram to approve higher risk participants for exercise evaluation.

### Treadmill testing procedure

Full details on treadmill testing procedures have been provided elsewhere [6–8]. Briefly, participants were asked to complete a series of up to twelve incrementally faster walking 5-min bouts on a level (0% grade) Cybex 751 T treadmill (Cybex International Inc, MA, USA). To facilitate collection and count of speed-specific steps from the tested wearable technologies, each bout was separated by a 2-min rest. Speed was verified using a tachometer and started at 0.8 km/h (0.5 mph) with subsequent increments of 0.8 km/h per bout to a maximum of 9.7 km/h (6.0 mph). The treadmill protocol was terminated at the end of the 5-min bout when the participant naturally transitioned from walking to jogging/running, achieved ≥ 75% of age predicted heart rate maximum, reported a Borg rating of perceived exertion > 13 [10] or if either the research staff or the participant decided not to continue for any reason (e.g., perceived fatigue or safety concerns).

### Measures

#### Participant characteristics and anthropometric measures

Biological sex, age, and race/ethnicity were self-reported. Participants’ weight, height, leg length, waist circumference, and BMI were measured using standardized protocols as detailed previously [8].

#### Step counting

The criterion measure of steps taken was directly observed and hand-tally counted. The method for assessing treadmill stepping was rarely problematic, likely because this was the sole assignment of one research technician during the treadmill test but also because the steps taken were largely rhythmic and predictable (except for the very few steps taken at the beginning and end of a bout) and the observed movements were reinforced with the audible sound made when the foot hit the treadmill band. We also aimed a video camera at the participant's feet during the test to provide a redundant copy of the event for verification purposes as needed. Our practice was that when the responsible research technician self-disclosed miscounting or the value reported was immediately identified as unusual or unexpected (i.e., higher or lower than expected given the preceding bout and/or recorded bout speed), the step count for that particular bout was verified and corrected as needed using the video file immediately following the testing session. During analysis, we had the opportunity to again examine rare cases of anomalous values (including questionable results compared to associated outputs from the multiple wearable technologies) by recounting steps on the video. If a discrepancy was found between the original logged value and the second viewing of the video, a third viewing was used to finalize the criterion value. We emphasize that this process was rarely required.

As mentioned above, data were collected over multiple years, and during this time, some wearable technologies were discontinued while others were updated and/or new ones became available. As a result, the exact number and description of devices differs somewhat between age groups. Ultimately, 21 different devices were evaluated over the full period of data collection. See Additional file 2: Suppl Fig. 1 and Suppl Table 1 for visual and tabular description of device locations, settings, distribution among age groups, and initialization and data extraction procedures: StepWatch (OrthoCare Innovations, Seattle, WA, USA) on the right ankle; an activPAL (PAL Technologies Ltd, Glasgow, UK) on the right thigh; an Actical (Philips Respironics, Murrysville, PA, USA), ActiGraph GT9X (ActiGraph, Pensacola, FL, USA), GENEActiv (Activinsights Ltd, Cambridgeshire, UK), New Lifestyles NL-1000 (New Lifestyles Inc., Lee’s Summit, MO, USA) and Fitbit One (Fitbit Inc, San Francisco, CA, USA) on the right waist, and a Digi-Walker SW-200 (Yamax Corporation, Tokyo, Japan), Fitbit Zip and PiezoRx (StepsCount, Ontario, Canada) on the left waist; an ActiGraph GT9X, Garmin vivoactive 3 (Garmin International Inc., Olathe, KS, USA), Garmin vivoactive HR, Garmin vivofit 2, Garmin vivofit 3, GENEActiv and Polar M600 (Polar Electro Oy, Kempele, Finland) on the non-dominant wrist, and an Apple Watch Series 1 (Apple Inc., Cupertino, CA USA), Fitbit Ionic (Fitbit Inc, San Francisco, CA, USA), Samsung Gear Fit2 (Samsung Electronics America Inc., Ridgefield Park, NJ, USA) and Samsung Gear Fit2 Pro on the dominant wrist.

### Data processing and aggregation

The Apple Watch Series 1, Digi-Walker SW-200, NL-1000, PiezoRx, Polar M600, and all Fitbit, Garmin and Samsung devices displayed step count data in real-time that was manually recorded at the end of each bout. For the waist- and wrist-worn GENEActiv, we used the step detection algorithm that we recently published [11]. The Actical, ActiGraph GT9X, activPAL, GENEActiv, and StepWatch recorded steps automatically time-stamped according to internal functioning. These data were downloaded according to manufacturers’ specifications as detailed in Additional file 2: Suppl Table 1. Specifically, the time-stamped step count data were synchronized to the study protocol’s digital timing record to facilitate post-processing of bout-specific step counts. Therefore, each wearable technology was managed to provide a total number of steps per bout, and these, along with the directly observed step data, were merged into a single comma-delimited flat file for further analysis.

### Analytic sample

The final analytic data set included 258/260 originally recruited participants after removing data from two women (84.5 ± 0.7 years of age) whose participation was terminated due to safety concerns identified as unsteadiness during treadmill ambulation. Sample sizes linked to each wearable technology varied due to the fact that some devices were worn by all age groups while some others were only available (and thus worn) in specific age groups over the multiple years of data collection of the original study. Further, some individual devices malfunctioned and therefore these specific data were lost. A full description of sample sizes and number of steps derived from direct observation and each tested wearable technology at each treadmill speed by age group is provided in Additional file 3.

Ultimately, the sample of 258 participants provided 1,842 treadmill bouts, with 30 of which being running bouts. Following the same procedures established in the previous catalog based on the CADENCE-kids study [12], we decided to exclude running bouts from this analysis for three specific reasons: 1) the lack of robustness of the sample size providing these bouts (running bouts represented only the 1.6% of total bouts); 2) the speeds at which people actually ran varied from 4.8 to 8.8 km/h, making conclusions challenging about any specific speed); and, 3) the well-known biomechanical differences between running and walking [13]. Thus, the final analytical data set of 258 participants comprised a total of 1,812 treadmill walking bouts ranging from slow to fast speeds. The data set and the corresponding data dictionary were formatted in accordance with the previously published catalog [9] and are available in Additional file 4.

### Statistical analysis

#### Descriptive statistics

Sample characteristics are presented as means and SDs or percentages (%), as appropriate. We previously defined and rationalized validity indices related to accuracy, bias, and precision [9] yet are briefly reviewed again here. Accuracy was determined using MAPE, calculated as follows [4].

$${E}_{j}={W}_{j}- \, {C}_{j}$$

$$\mathrm{MAPE}= \frac{100\%}{n}{\sum }_{j=1}^{n}\frac{\left|{E}_{j}\right|}{{C}_{j}}$$

where *W*_*j*_ is the number of steps recorded by the device being tested in the *j*^th^ person-bout (*j* = 1, 2, …, *n*), *C*_*j*_ is the criterion measure of directly observed steps in that same person-bout, and *E*_*j*_ is the corresponding step count error expressed in absolute terms.

Bias was represented as MPE, calculated as follows [14]:

$$\mathrm{MPE}= \frac{100\%}{n}{\sum }_{j=1}^{n}\frac{{E}_{j}}{{C}_{j}}$$

By dividing the difference in steps derived from wearable technology and the directly observed steps (*E*_*j*_) by the directly observed steps (*C*_*j*_), the result is a scaled index that explains the difference, regardless of the total number of steps taken.

Precision indices were: SD, CoV and correlation coefficient (*r*) [15]. SD of error values (*E*) was calculated as follows:

$$\mathrm{SD}=\sqrt{ \frac{1}{{\text{n}}}{\sum }_{{\text{j}}= \text{1}}^{\text{n}}({\text{E}}_{\text{j}}-{\overline{E })}^{2}}$$

CoV was calculated as:

$$\mathrm{CoV}=\left(\frac{\mathrm{SD}}{\overline{E} }\right)\times 100\%$$

where *SD* represents the wearable technology’s variance in steps, and $\overline{E }$ is the average of errors. Finally, the Pearson correlation coefficient (*r*) representing the strength of the relationship between directly observed steps and steps derived from wearable was computed accordingly:

$$r=\frac{{\sum }_{j=1}^{n}{(W}_{j}-\overline{W }){(C_{j}-\overline{C })}}{\sqrt{\left[{\sum }_{j=1}^{n}{(W_{j}-\overline{W })}^{2}\right]\left[{\sum }_{j=1}^{n}{(C_{j}-\overline{C })}^{2}\right]}}$$

where *W*_*j*_ is the wearable technology’s number of steps being tested in the *j*^*th*^ person-bout (j = 1, 2, …, n), and *C*_*j*_ is the observed steps in that same person-bout.

Again, following the procedures established in our previously published children/youth catalog [9], MAPE (accuracy) and MPE (bias) values, with their associated SD and CoV (precision) values, were averaged across the available samples for each wearable technology, and presented for each walking speed, speed level (i.e., slow speed level = 0.8, 1.6, 2.4, and 3.2 km/h; normal speed level = 4.0, 4.8, 5.6, and 6.4 km/h; and fast speed level = 7.2 and 8.0 km/h), wear location (ankle, thigh, waist, and wrist), and age group (young adults, 21–40 years; middle-age adults, 41–60 years; and older adults, 61–85 years). Correlation coefficients (*r*) were computed for the whole sample and reported across all walking bouts as these required a wider range of step counts to provide meaningful results. To classify speed levels, we defined slow and fast relative to (and accepting of) the Consumer Technology Association (CTA) description of a normal speed range [5]. Interpretation of validation indices adhered to accepted conventions. For example, the lower the MAPE the better the accuracy. Similarly, the closer the MPE values to 0% the better the bias. Lower SDs and CoV were interpreted as better precision. Also, correlation coefficients closer to 1 indicated better precision.

#### Inferential analysis

The effects of speed, wear location and age group on accuracy, bias, and precision were tested via mixed effect models. First, we tested the effect of speed on MAPE by fitting a set of 21 mixed effects models for each of the 21 tested wearable technologies. Thus, the MAPE for participant *i* = 1, 2, …, *N* at speed *j* = 1, 2, …, *q* (inserted in the model as a categorical variable), conditional on their participant-specific deviation, was estimated for each device as follows:

$$E[{Y}_{i}|{b}_{i}]={{\varvec{X}}}_{{\varvec{i}}}\beta +{b}_{i}$$

where *Y*_*i*_ is a *q* × *1* vector of absolute percentage error values, ***X***_*i*_ represents a *q* × *q* diagonal matrix of dummy variables (i.e., equal to 0 or 1) indexing the corresponding speed, *β* is a *q* × *1* vector of regression coefficients for the fixed effect (i.e., speed as categorical variable), and *b*_*i*_ represents the random intercept for a participant *i*. To test the effect of speed (*β*) on MAPE, likelihood ratio tests (α = 0.05) were used for each wearable technology-specific model. We also estimated 95% CIs of MAPE at each speed. Congruent with the direction of our previously published approach [9] and with previous indications [16], 95% CIs were interpreted as significantly different when they did not overlap with another point estimate. When the CIs overlapped, statistical significance was not clear. Another valid approach would be to construct CIs around the differences. However, we chose not to do that because the statistically unclear differences were practically small, irrespective of statistical significance. We used the same mixed model analysis to examine the effect of wear location and age group. To do so, we substituted for ***X***_*i*_ and refitted the model separately for each of the three speed levels (i.e., slow, normal, and fast). For example, to test the effect of age group on MAPE for each of the speed levels, we treated ***X***_*i*_ as a diagonal matrix of dummy variables (equal to 0 or 1) corresponding to age-speed combinations. Main analyses of the present study were performed and are presented for wearable technologies’ MAPE since accuracy reflects both bias and precision as it accounts for the overall performance of a step counting device [15]. Additionally, all mixed model analyses were used to examine the effects of speed, wear location and age on bias (MPE) and precision (*r*) and are presented as supplementary material. All analyses were performed using R-Studio (version 3.0.2, R Foundation for Statistical Computing, Vienna, Austria).

## Results

### Descriptive statistics

#### Sample characteristics

Table 1 presents descriptive characteristics for the whole sample (*N* = 258) and by age group. Also, Additional file 3 includes the total sample sizes for those who completed each walking bout and the average number of steps counted at each speed by direct observation and by each of the wearable technologies. Only four young adults (23.8 mean years of age) and one middle-aged participant (50 years of age) reached the maximum observed speed of 8.0 km/h (5.0 mph), while no older adults (61–85 years of age) achieved this speed.

**Table 1:** Descriptive characteristics of the participants

Variable	All (*N* = 258)	Young Adults, 21–40 years (*N* = 80)	Middle-Age Adults, 41–60 years (*N* = 80)	Older Adults, 61–85 years (*N* = 98)
Sex, n male (%)	130 (50.4)	40 (50.0)	40 (50.0)	50 (51.0)
Age (years)	52.5 ± 18.7	30.1 ± 5.8	50.2 ± 5.9	72.6 ± 6.9
Height (cm)	169.5 ± 9.1	170.7 ± 9.2	171.0 ± 9.2	167.3 ± 8.5
Weight (kg)	73.8 ± 14.0	72.5 ± 14.0	76.3 ± 14.2	72.7 ± 12.6
Leg Length (cm)	80.1 ± 5.4	79.7 ± 5.8	80.7 ± 5.2	79.9 ± 5.2
Waist circumference (cm)	85.6 ± 11.7	79.8 ± 10.7	86.2 ± 10.8	89.8 ± 11.3
BMI	25.6 ± 3.6	24.8 ± 3.4	26.0 ± 4.0	25.9 ± 3.5
BMI Classification, n (%)				
Normal	127 (49.2)	47 (58.8)	37 (46.2)	43 (43.9)
Overweight	105 (40.7)	29 (36.2)	31 (38.8)	45 (45.9)
Obese	26 (10.1)	4 (5.0)	12 (15.0)	10 (10.2)
Race/ethnicity, n (%)				
White	201 (77.9)	49 (61.3)	68 (85.0)	84 (85.7)
African-American	5 (1.9)	2 (2.5)	2 (2.5)	1 (1.0)
Hispanic	6 (2.3)	4 (5.0)	2 (2.5)	0 (0.0)
Asian	18 (7)	16 (20.0)	1 (1.2)	1 (1.0)
American Indian	3 (1.2)	1 (1.2)	0 (0.0)	2 (2.0)
Other	5 (1.9)	2 (2.5)	3 (3.8)	0 (0.0)
Unknown/No response	14 (5.4)	3 (3.8)	2 (2.5)	9 (9.2)
More than one	6 (2.3)	3 (3.8)	2 (2.5)	1 (1.0)

Values are means ± standard deviation or number of cases (percentages). *BMI* Body Mass Index (kg/m^2^). BMI classifications: Normal or healthy weight (18.5–24.9 kg/m^2^), overweight (25.0–29.9 kg/m^2^), obese (≥ 30 kg/m^2^)

#### Accuracy, bias, and precision by speed

**Fig. 1:**
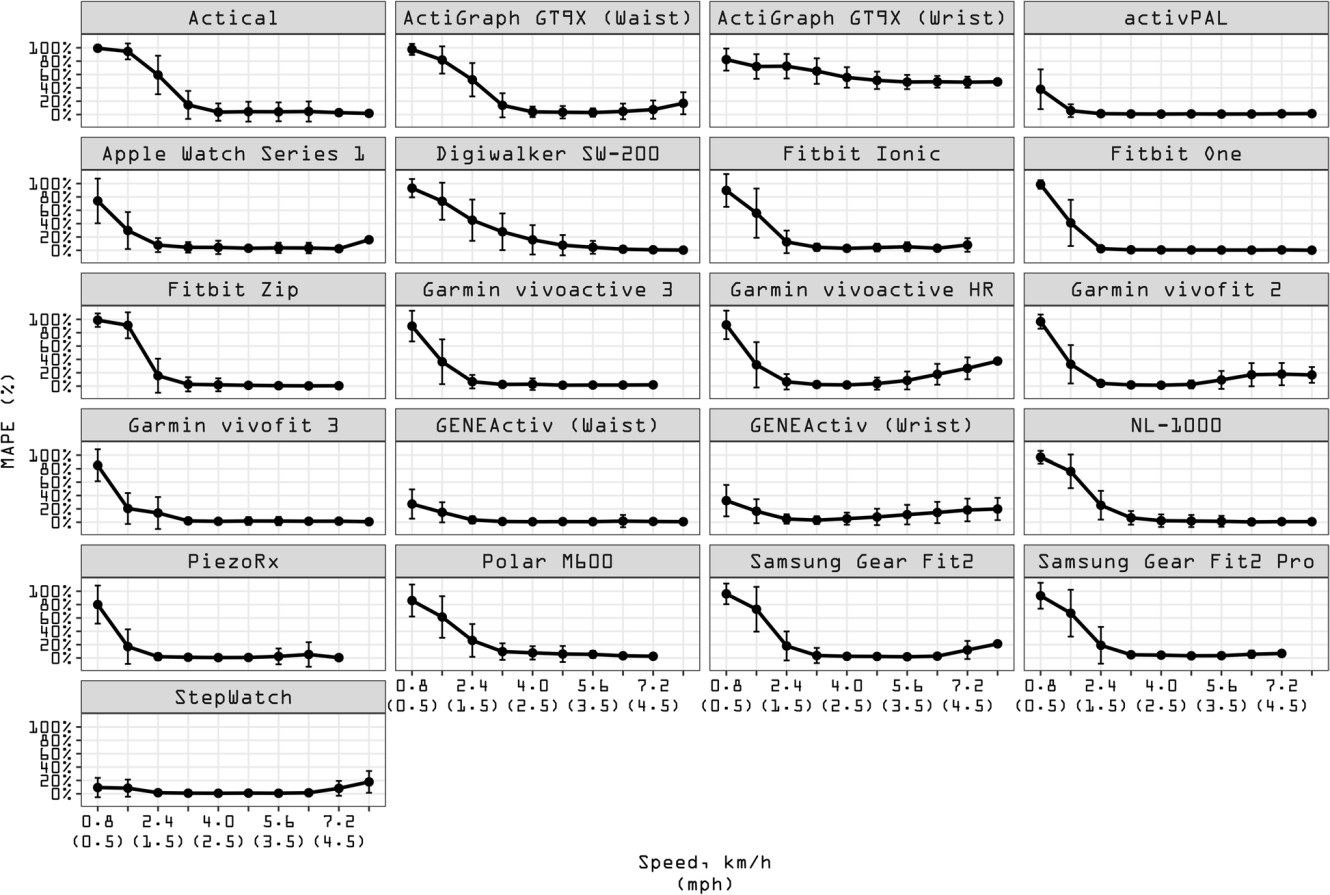
Mean absolute percentage error (MAPE) of each wearable technology across walking speeds. Participants walked on a treadmill for 5-min bouts beginning at 0.8 km/h (0.5 mph) and increasing in 0.8 km/h (0.5 mph). MAPE (%) was computed for each person bout subtracting the directly observed steps (criterion measurement) from the wearable technology-derived steps and dividing it in absolute value by the directly observed steps. Black dots represent the averaged MAPE across all sample for a given speed. Bars represent standard deviation of MAPE. The standard deviation bars were not drawn when they were shorter than the height of the symbol. Lower MAPE values indicate higher accuracy of the wearable technology of interest. See Additional file 2 for a graphical classification of wearable technologies by age groups

Additional file 5 includes an interactive digital catalog of validity indices of MAPE, MPE, SD and CoV values indicating the tested wearable technologies’ step counting performance compared to direct observation for different speeds, wear locations, and age groups. As shown in the catalog and in Fig. 1, the activPAL, Fitbit One, Fitbit Zip, Garmin vivoactive 3, Garmin vivofit 3, waist-worn GENEActiv, NL-1000, PiezoRx, Samsung Gear Fit2 and StepWatch displayed the highest accuracy (MAPE = 1–2%) over the range of normal speeds (4.0–6.4 km/h). These devices’ accuracy was followed by the Actical, waist-worn ActiGraph GT9X, Apple Watch Series 1, Fitbit Ionic, Samsung Gear Fit2 Pro (all MAPE = 4%), the Polar M600 (6%), the Garmin vivoactive HR and Garmin vivofit 2 (both MAPE = 7%), the Digiwalker SW-200 (8%), and the wrist-worn GENEActiv (9%). In contrast, the wrist-worn ActiGraph GT9X displayed the worst accuracy across normal speeds (MAPE = 52%). Over the whole range of slow, normal and fast speed levels, the StepWatch displayed the best accuracy (MAPE = 3%), followed by the activPAL and waist-worn GENEActiv (both MAPE = 7%) and by a group of devices (Apple Watch Series 1, Fitbit One, Garmin vivofit 3, wrist-worn GENEActiv and PiezoRx) that performed at an accuracy between 12–19% MAPE. Fitbit Ionic, Garmin vivoactive 3, Garmin vivoactive HR, Garmin vivofit 2, NL-1000, and Samsung Gear Fit2 displayed an accuracy between 20–29% MAPE. Actical, waist-worn ActiGraph GT9X, Digiwalker SW-200, Fitbit Zip, Polar M600, and Samsung Gear Fit2 Pro displayed an accuracy between 30–39% MAPE, while the wrist-worn ActiGraph GT9X performed at the worst accuracy among devices (MAPE = 63%). On average, accuracy for all devices was compromised across slow walking speeds (MAPE = 40 ± 40%). The best performance in terms of accuracy was observed for all devices on average across normal speeds (MAPE = 7 ± 16%).

Additional file 6: Suppl Fig. 1 depicts MPE values and the corresponding SD for each wearable technology across walking speeds. On average, the greater bias was observed across slow walking speeds by all devices (MPE = -37 ± 43%), while the best bias values were observed across the normal speeds (MPE = -6 ± 17%). Additional file 7: Suppl Fig. 1 also includes the correlation coefficients depicting the strength of the relationship between directly observed steps and those derived from each wearable technology. The StepWatch, activPAL, and Fitbit One showed the strongest correlation with directly observed steps (*r* = 0.97, 0.96, 0.94, respectively), while the wrist-worn ActiGraph, wrist-worn GENEActiv, and Fitbit Zip showed the weakest correlation (*r* = 0.76, 0.80, 0.80, respectively).

#### Accuracy, bias, and precision by wear location

**Fig. 2:**
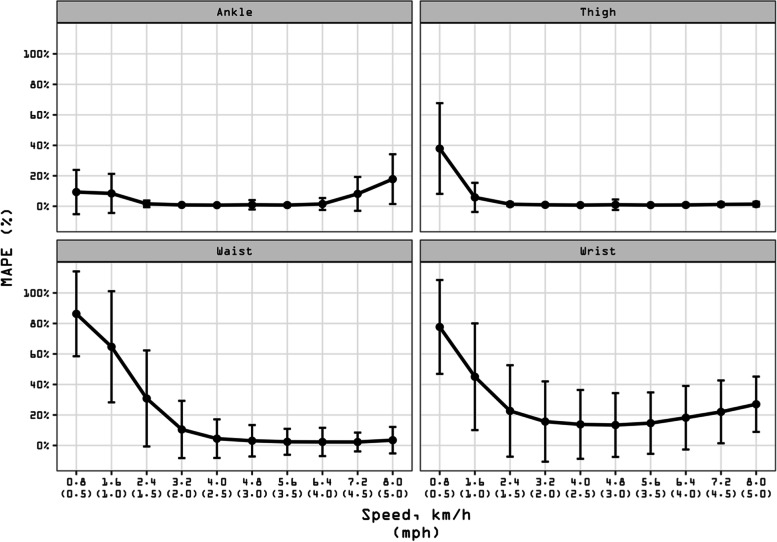
Mean absolute percentage error (MAPE) across walking speeds presented by wear location. Participants walked on a treadmill for 5-min bouts beginning at 0.8 km/h (0.5 mph) and increasing in 0.8 km/h (0.5 mph). MAPE (%) was computed for each person bout subtracting the directly observed steps (criterion measurement) from the wearable technology-derived steps and dividing it in absolute value by the directly observed steps. Black dots represent the averaged MAPE across specific wear location for a given speed. Bars represent standard deviation of MAPE. The standard deviation bars were not drawn when they were shorter than the height of the symbol. Lower MAPE values indicate higher wear location accuracy. Ankle-worn wearable: StepWatch (*N* = 253). Thigh-worn wearable: activPAL (*N* = 249). Waist-worn wearables: Actical (*N* = 250), ActiGraph GT9X (*N* = 254), Digi-Walker SW-200 (*N* = 258), Fitbit One (*N* = 160), Fitbit Zip (*N* = 98), GENEActiv (*N* = 224), NL-1000 (*N* = 258), PiezoRx (*N* = 98). Wrist-worn wearables: ActiGraph GT9X (*N* = 254), Apple Watch Series 1 (*N* = 174), Fitbit Ionic (*N* = 98), Garmin vivoactive 3 (*N* = 96), Garmin vivoactive HR (*N* = 77), Garmin vivofit 2 (*N* = 80), Garmin vivofit 3 (*N* = 77), GENEActiv (*N* = 217), Polar M600 (*N* = 97), Samsung Gear Fit2 (*N* = 80), Samsung Gear Fit2 Pro (*N* = 98). See Additional file 2 for a graphical classification of wearable technologies by age groups and Additional File 8: Suppl Table 1 for a tabular description of validity indices by wear locations

Figure 2 presents MAPE values at each speed for the four different wear locations (see also Additional file 8: Suppl Table 1 for a tabular description of validity indices). Over the range of normal speeds, the ankle and thigh locations displayed the best accuracy (both MAPE = 1 ± 2%), followed by the waist (3 ± 11%). The wrist displayed the worst accuracy (MAPE = 15 ± 21%). When considering the whole range of speeds, the ankle displayed the best accuracy (MAPE = 3 ± 8%), followed by the thigh (7 ± 17%), while the waist and wrist locations showed reduced accuracy (MAPE = 28 ± 39% and 30 ± 35%, respectively).

Additional file 8: Suppl Table 1 also presents the MPE values at each speed bout for each wear location. Over the whole range of speeds, the ankle displayed the best bias values (MPE = 0 ± 9%), followed by the thigh (MPE = -6 ± 18%). The waist and wrist displayed the worst bias (both MPE = -27%). Additional file 9: Suppl Fig. 2 presents the correlation coefficients between directly observed steps and those detected by device averaged across wear location. The ankle and the thigh displayed a mean correlation of *r* = 1.0, while the waist and wrist displayed a mean correlation of *r* = 0.9.

#### Accuracy, bias, and precision by age group

**Fig. 3:**
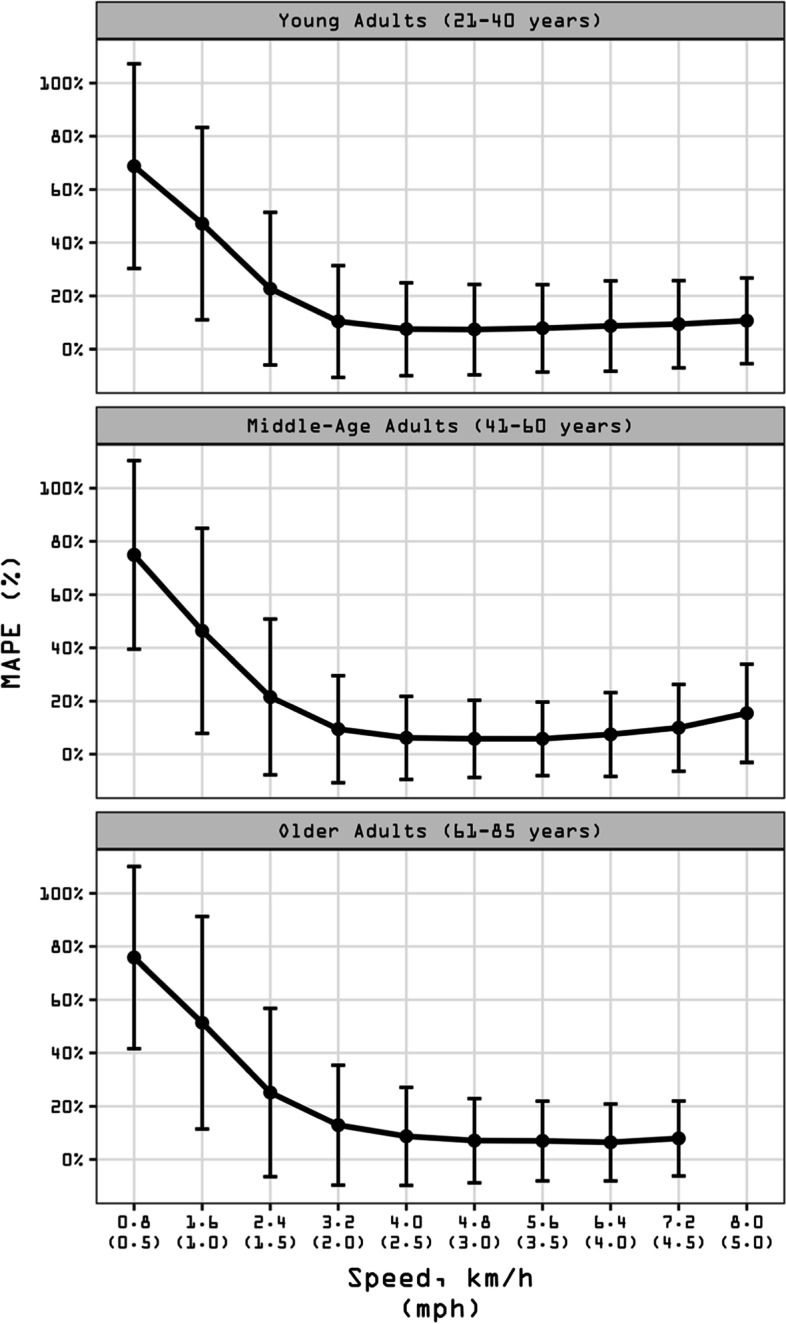
Mean absolute percentage error (MAPE) across walking speeds presented by age group. Participants walked on a treadmill for 5-min bouts beginning at 0.8 km/h (0.5 mph) and increasing in 0.8 km/h (0.5 mph). MAPE (%) was computed for each person bout subtracting the directly observed steps (criterion measurement) from the wearable technology-derived steps and dividing it in absolute value by the directly observed steps. Black dots represent the averaged MAPE across specific age group for a given speed. Bars represent standard deviation of MAPE. The standard deviation bars were not drawn when they were shorter than the height of the symbol. Lower MAPE values indicate higher age group accuracy. All age groups (21–85 years) wore the Actical (*N* = 250), ActiGraph GT9X (Waist) (*N* = 254), ActiGraph GT9X (Wrist) (*N* = 254), activPAL (*N* = 249), Digi-Walker SW-200 (*N* = 258), GENEActiv (Waist) (*N* = 224), GENEActiv (Wrist) (*N* = 217), NL-1000 (*N* = 258), and the StepWatch (*N* = 253). Young Adults (21–40 years) also wore the Fitbit One (*N* = 80) and Garmin vivofit 2 (*N* = 80). Middle-Age Adults (41–60 years) also wore the Apple Watch Series 1 (*N* = 76), Fitbit One (*N* = 80), Garmin vivoactive HR (*N* = 77), Garmin vivofit 3 (*N* = 77), and the Samsung Gear Fit2 (*N* = 80). Older Adults (61–85 years) also wore the AppleWatch Series 1 (*N* = 98), Fitbit Ionic (*N* = 98), Fitbit Zip (*N* = 98), Garmin vivoactive 3 (*N* = 96), PiezoRx (*N* = 98), Polar M600 (*N* = 97), and the Samsung Gear Fit2 Pro (*N* = 98). See Additional file 2 for a graphical classification of wearable technologies by age groups. See Additional File 8: Suppl Table 2 for a tabular description of validity indices by age groups

Figure 3 presents MAPE values at each speed for the three age groups (see also Additional file 8: Suppl Table 2). The best accuracy was reported for middle-aged adults across normal speeds (MAPE = 6 ± 15%) followed closely by young and older adults (both 8 ± 17%). The worst accuracy was reported for older adults across slow speeds (43 ± 44%), followed closely by middle-age and young adults (38 ± 40% and 37 ± 39%, respectively). Over the whole range of speeds, young and middle-aged adults displayed similar accuracy (MAPE = 23%), and older adults displayed worse accuracy (MAPE = 30%).

Average MPE values by speed and age group are presented in Additional file 8: Suppl Table 2. Young and middle-aged adults displayed the best bias values (MPE = -20 ± 35% and -21 ± 36%, respectively), while older adults displayed the worst bias (-28 ± 39%). Correlation coefficients between directly observed steps and device-detected steps averaged across each age groups are in Additional file 9: Suppl Fig. 3. The mean correlation was 0.9 for both young and middle-age adults compared with 0.8 for older adults.

### Inferential analyses

#### Effect of speed on accuracy, bias, and precision

**Fig. 4:**
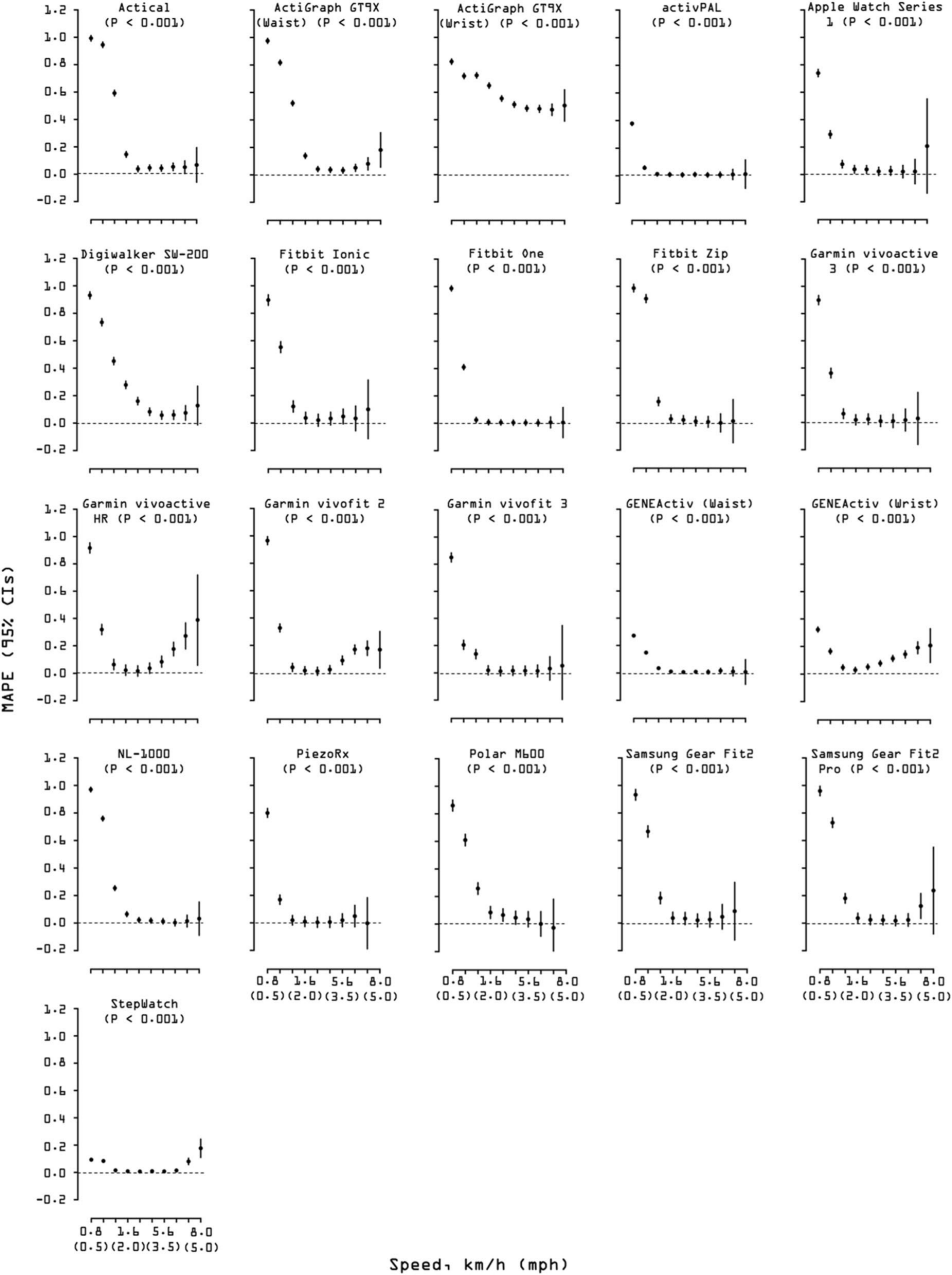
Effect of speed on overall accuracy (mean absolute percentage error, MAPE) of wearable technology’s step counting ability. MAPE and corresponding 95% confidence intervals (CIs) respective to each technology are plotted across speed bouts. Slow speed bouts: 0.8, 1.6, 2.4, 3.2 km/h (0.5, 1.0, 1.5, 2.0 mph); normal speed bouts: 4.0, 4.8, 5.6, 6.4 km/h (2.5, 3.0, 3.5, 4.0 mph); fast speed bouts: 7.2, 8.0 km/h (4.5, 5.0 mph). Each black dot represents grouped averages of MAPE values, with 95% CIs estimated using mixed effect models and extending above and below that point estimate. The 95% CIs bars were not drawn when they were shorter than the height of the symbol. MAPE values closer to 0 (indicated by a dashed line) are indicative of greater accuracy. 95% CIs bars that do not overlap are interpreted as significantly different among them. Likelihood ratio test *P* value is reported for the effect of all speeds on MAPE for each specific device. See Additional file 2 for a graphical classification of wearable technologies’ location by age groups

There was an overall significant effect of speed on accuracy (*p* < 0.001; Fig. 4) that was driven by an increased MAPE at slow speeds (0.8–3.2 km/h). That is, 19/21 tested devices displayed a significantly reduced accuracy at 0.8 km/h compared to 1.61 km/h or 2.4 km/h, except the wrist-worn ActiGraph GT9X (MAPE, 95% CI = 0.72, 0.70–0.74 at 1.6 km/h and 0.72, 0.70–0.74 at 2.4 km/h) and the StepWatch (MAPE, 95% CI = 0.09, 0.08–0.10 at 0.8 km/h and 0.08, 0.07–0.10 at 1.6 km/h). Only three devices displayed significant differences in accuracy (*p* < 0.001) across normal walking speeds: the Digiwalker SW-200 (MAPE, 95% CI = 0.16, 0.13–0.19 at 4.0 km/h and 0.08, 0.06–0.11 at 4.8 km/h), the Garmin vivoactive HR (MAPE, 95% CI = 0.08, 0.04–0.12 at 5.6 km/h and 0.18, 0.13–0.23 at 6.4 km/h), and the Garmin vivofit 2 (MAPE, 95% CI = 0.09, 0.06–0.12 at 5.6 km/h and 0.17, 0.14–0.20 at 6.4 km/h). Over the range of fast speeds, only the StepWatch (MAPE, 95% CI = 0.08, 0.06–0.10 at 7.2 km/h and 0.18, 0.11–0.24 at 8.0 km/h) displayed a significant difference in accuracy (*p* < 0.001).

MPE results paralleled those of MAPE. We observed significant differences in bias occurring mainly across slow walking speeds (*p* < 0.001; Additional file 10: Suppl Fig. 1). There was no significant speed effect on precision as defined by correlation coefficients computed between directly observed steps and those derived from the tested wearable technologies (*p* = 0.120; Additional file 9: Suppl Fig. 1).

#### Effect of wear location on accuracy, bias, and precision

**Fig. 5:**
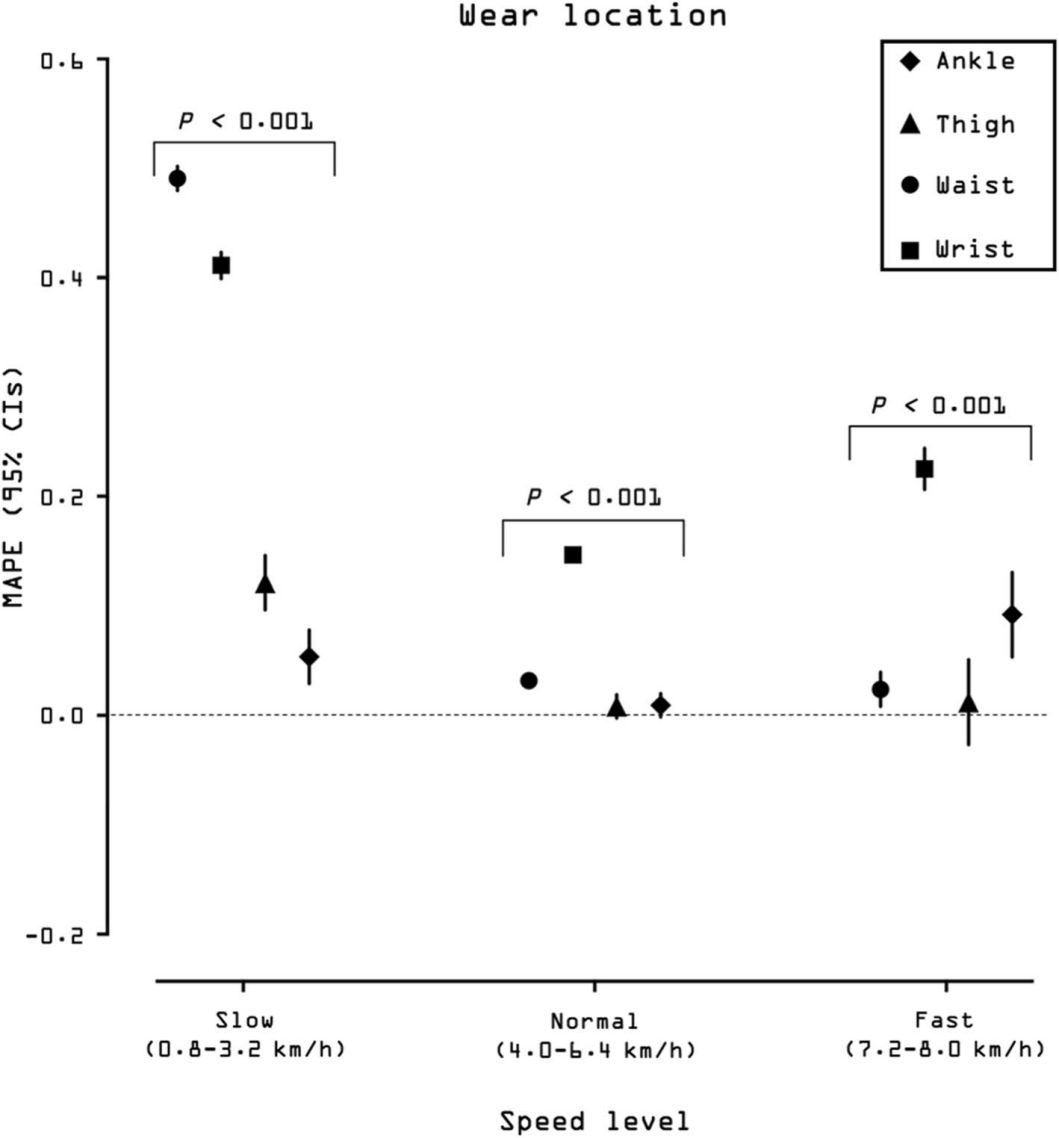
Effect of wear location on overall accuracy (mean absolute percentage error, MAPE) of wearable technologies’ step counting ability. MAPE and corresponding 95% confidence intervals (CIs; estimated using mixed effect models) of each wear location are presented at slow, normal, and fast walking speeds. Slow speed bouts: 0.8, 1.6, 2.4, 3.2 km/h (0.5, 1.0, 1.5, 2.0 mph); normal speed bouts: 4.0, 4.8, 5.6, 6.4 km/h (2.5, 3.0, 3.5, 4.0 mph); fast speed bouts: 7.2, 8.0 km/h (4.5, 5.0 mph). MAPE values were averaged across devices respective to each wear location for slow, normal, and fast walking speeds. MAPE values closer to 0 indicate greater accuracy. The 95% CIs bars were not drawn when they were shorter than the height of the symbol. Further, where 95% CIs do not overlap, there are significant differences between locations. Likelihood ratio test* P* value is reported for the effect of wear location on MAPE for each specific speed level. Ankle-worn wearable: StepWatch (*N* = 253). Thigh-worn wearable: activPAL (*N* = 249). Waist-worn wearables: Actical (*N* = 250), ActiGraph GT9X (*N* = 254), Digi-Walker SW-200 (*N* = 258), Fitbit One (*N* = 160), Fitbit Zip (*N* = 98), GENEActiv (*N* = 224), NL-1000 (*N* = 258), PiezoRx (*N* = 98). Wrist-worn wearables: ActiGraph GT9X (*N* = 254), Apple Watch Series 1 (*N* = 174), Fitbit Ionic (*N* = 98), Garmin vivoactive 3 (*N* = 96), Garmin vivoactive HR (*N* = 77), Garmin vivofit 2 (*N* = 80), Garmin vivofit 3 (*N* = 77), GENEActiv (*N* = 217), Polar M600 (*N* = 97), Samsung Gear Fit2 (*N* = 80), Samsung Gear Fit2 Pro (*N* = 98). See Additional file 2 for a graphical classification of wearable technologies by age groups

Wear location was a significant factor in determining overall accuracy of device performance across all walking speeds (*p* < 0.001; Fig. 5). The waist and wrist locations displayed significantly reduced accuracy (MAPE, 95% CI = 0.49, 0.48–0.50 and 0.41, 0.40–0.42, respectively) compared with the ankle and thigh (MAPE, 95% CI = 0.05, 0.03–0.08 and 0.12, 0.10–0.15, respectively), indicating a reduced relative accuracy of the waist- and wrist-worn devices at slow speeds. Over the range of normal speeds, the ankle and thigh displayed exactly the same accuracy (MAPE, 95% CI = 0.01, 0.00–0.02; not significantly different, *p* < 0.05), while the waist and wrist showed significantly reduced accuracy (MAPE, 95% CI = 0.03, 0.04–0.05 and 0.14, 0.13–0.15, respectively). Regarding the fast walking speed, the wrist displayed the worst accuracy (MAPE, 95% CI = 0.23, 0.21–0.24), followed by the ankle (MAPE, 95% CI = 0.09, 0.05–0.13). There was no difference in accuracy between thigh and waist locations (MAPE, 95% CI = 0.01, -0.03–0.05 and 0.02, 0.01–0.04, respectively) at the fast speed level.

Similar wear location effects were observed for bias as indicated by MPE (Additional file 10: Suppl Fig. 2). There was a significant wear location effect on precision when testing the relationship between directly observed steps and those derived from wearable technologies (*p* = 0.045; Additional file 9: Suppl Fig. 2).

#### Effect of age on accuracy, bias, and precision

**Fig. 6:**
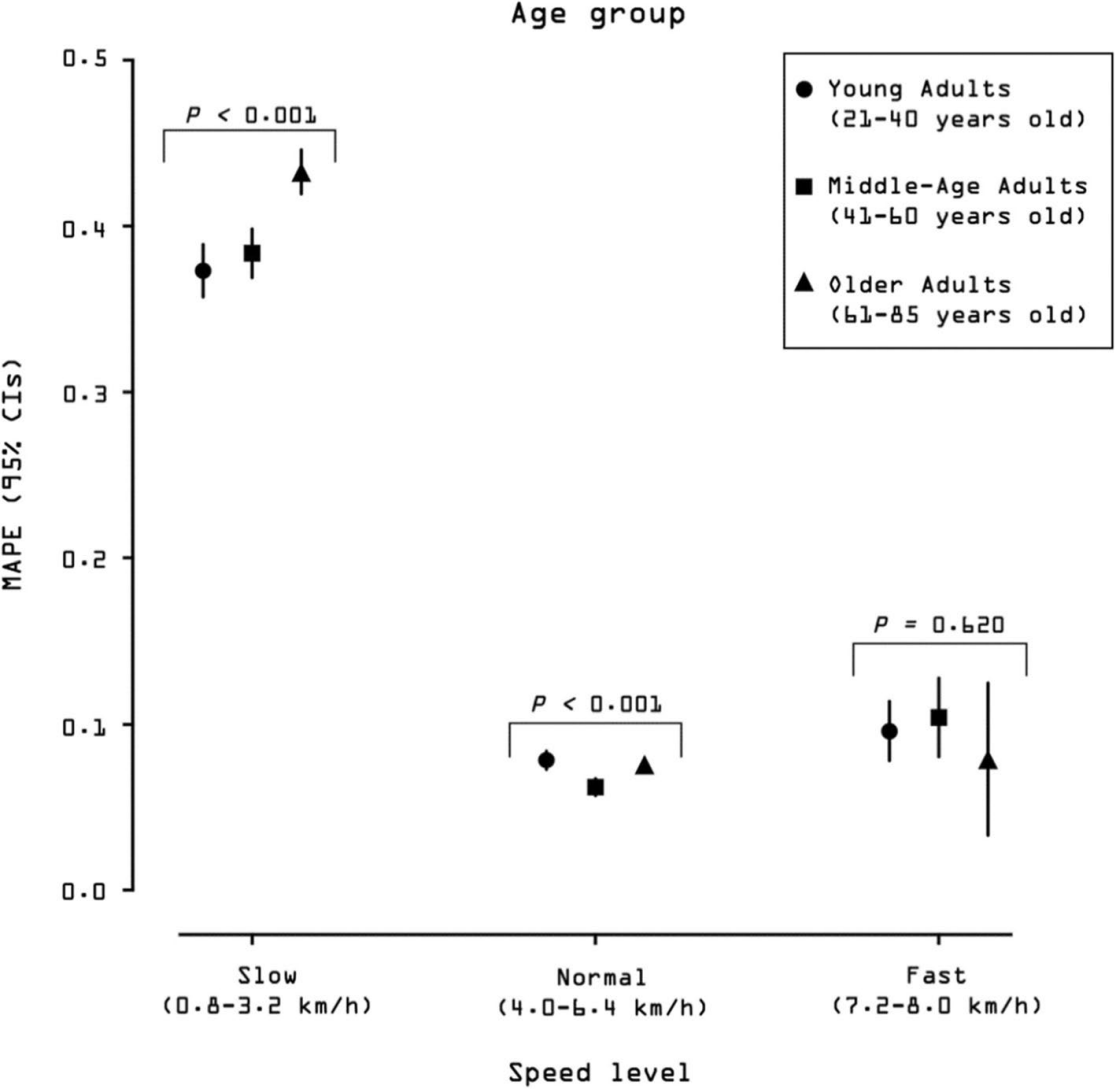
Effect of age on overall accuracy (mean absolute percentage error, MAPE) of wearable technologies’ step counting ability. MAPE and corresponding 95% confidence intervals (CIs; estimated using mixed effect models) of each age group are presented at slow, normal, and fast walking speeds. MAPE values were averaged across devices respective to each age group for slow, normal, and fast walking speeds. Slow speed bouts: 0.8, 1.6, 2.4, 3.2 km/h (0.5, 1.0, 1.5, 2.0 mph); normal speed bouts: 4.0, 4.8, 5.6, 6.4 km/h (2.5, 3.0, 3.5, 4.0 mph); fast speed bouts: 7.2, 8.0 km/h (4.5, 5.0 mph). MAPE values closer to 0 represent greater accuracy. The 95% CIs bars were not drawn when they were shorter than the height of the symbol. Further, where 95% CIs do not overlap, there are significant differences between locations. Likelihood ratio test* P* value is reported for the effect of age on MAPE for each specific speed level. All age groups (21–85 years) wore the Actical (*N* = 250), ActiGraph GT9X (Waist) (*N* = 254), ActiGraph GT9X (Wrist) (*N* = 254), activPAL (*N* = 249), Digi-Walker SW-200 (*N* = 258), GENEActiv (Waist) (*N* = 224), GENEActiv (Wrist) (*N* = 217), NL-1000 (*N* = 258), and the StepWatch (*N* = 253). Young Adults (21–40 years) also wore the Fitbit One (*N* = 80) and Garmin vivofit 2 (*N* = 80). Middle-Age Adults (41–60 years) also wore the Apple Watch Series 1 (*N* = 76), Fitbit One (*N* = 80), Garmin vivoactive HR (*N* = 77), Garmin vivofit 3 (*N* = 77), and the Samsung Gear Fit2 (*N* = 80). Older Adults (61–85 years) also wore the AppleWatch Series 1 (*N* = 98), Fitbit Ionic (*N* = 98), Fitbit Zip (*N* = 98), Garmin vivoactive 3 (*N* = 96), PiezoRx (*N* = 98), Polar M600 (*N* = 97), and the Samsung Gear Fit2 Pro (*N* = 98). See Additional file 2 for a graphical classification of wearable technologies by age groups

The regression models indicated that age had a significant effect on the step counting accuracy at slow and normal walking speed levels (both *p’s* < 0.001; Fig. 6). The wearable technologies displayed a significantly reduced accuracy when tested on the older adults (MAPE, 95% CI = 0.45, 0.42–0.43) compared to the young and middle-aged adults (MAPE, 95% CI = 0.37, 0.36–0.39 and 0.38, 0.37–0.40, respectively) across slow speeds. These two latter age groups did not differ in accuracy. Over the range of normal speeds, devices tested on the middle-aged adults showed significantly better accuracy (MAPE, 95% CI = 0.06, 0.05–0.06) compared to the young and older groups who displayed the same accuracy (MAPE, 95% CI = 0.08, 0.07–0.08). There was no difference observed in accuracy between age groups at the faster speeds (*p* = *0.620*).

Similar findings were observed for the effect of age group on bias (Additional file 10: Suppl Fig. 3). A significant effect of age group was observed on precision (*p* = 0*.*012; Additional file 9: Suppl Fig. 3).

## Discussion

The interactive digital catalog produced herein (Additional file 5) is the single largest cohesive assemblage of multiple step counting wearable technologies (representing different wear locations) tested using a standardized multi-speed treadmill protocol across the adult lifespan of 21–85 years of age. The catalog was designed to expand upon our previously published similar work in 6–20-year-old children/youth [9]. Together, this body of work addresses knowledge gaps identified in our earlier scoping review [4] that examined the state of the scientific literature related to standardizing step counting wearable technology validation protocols. Both catalogs (Additional file 5 for adults and Additional file 6 in the children/youth study [9] can be searched for any of the specific validity indices computed (i.e., MAPE, MPE, SD, CoV, *r*) and filtered to examine and compare device performance. Researchers may wish to consult these catalogs when selecting appropriate wearable technologies for their specific purposes after having considered their target populations and desired validity profiles. They may also use these indices to help compare and interpret results of studies that have employed different devices. Moreover, manufacturers of wearable technologies may wish to refer to these validity indices when developing new devices and setting their performance criteria.

The CTA previously suggested that step counting wearable technologies should perform within a MAPE of ≤ 10% [5]. Other researchers have suggested that MAPE values ≤ 5% do not have “practical relevance” [17, 18]. However, Hatano [19] reported that the Japanese Ministry of Economy Trade and Industry expected pedometers to perform at a MAPE ≤ 3%. The empirical basis for any of this guidance was limited before we conducted a scoping review [4] on the topic and identified eleven studies that provided weighted median MAPE values that were ≤ 1% for thigh-worn devices, 1–4% for waist-worn, and 7–11% for wrist-worn devices for treadmill speeds of 4.0 to 6.5 km/h. Even in lieu of this reporting, we described the literature as fractured and inconsistent and called for a standardized approach that includes validation indices such as MAPE for accuracy and employment of study designs that systematically test moderating factors such as speed, wear location, and age. Having achieved this herein, we can conclude that, in adults 21–85 years of age tested over the range of normal speeds (4.0–6.4 km/h), 15 devices (Actical, waist-worn ActiGraph GT9X, activPAL, Apple Watch Series 1, Fitbit Ionic, Fitbit One, Fitbit Zip, Garmin vivoactive 3, Garmin vivofit 3, waist-worn GENEActiv, NL-1000, PiezoRx, Samsung Gear Fit2, Samsung Gear Fit2 Pro, and StepWatch) performed at < 5% MAPE. The wrist-worn ActiGraph GT9X displayed the worst accuracy across normal speeds (MAPE = 52%). On average, accuracy was compromised across slow walking speeds for all wearable technologies (MAPE = 40%) while all performed best across normal speeds (MAPE = 7%). When analyzing the data by wear locations, the ankle and thigh demonstrated the best accuracy (both MAPE = 1%), followed by the waist (3%) and the wrist (15%) across normal walking speeds.

In the past, some wearable technologies have been referred to as “consumer-grade” (e.g., Fitbit, Samsung, etc.) and others as “research-grade” (e.g., ActivPAL, ActiGraph). We provide evidence herein that such off-handed labels do not necessarily imply quality based on measurement performance. As previously reported [9], the cost of the wearable technology also cannot be used to predict performance in terms of step count accuracy, bias, or precision. Case in point, a ~ $20 (US dollars) pedometer (e.g., Digi-Walker SW-200) displayed a better MAPE (1%) over normal speeds compared to a ~ $450 device (e.g., Actical; MAPE = 4%) tested at the same wear location that also requires a ~ $500 software to extract data. We have previously presented a more detailed discussion of the effects of speed, wear location, and age on the validity indices represented by accuracy, bias, and precision in children/youth [9]. In general, the present findings are similar to those reported from the children/youth catalog’s analysis [9]. Specifically, step counting wearable technologies perform best at normal speeds and wrist-based devices comparatively yield the worst accuracy. What stands out, however, is the nuanced findings in the children/youth catalog [9] that speed and wear location affected accuracy and bias, with no apparent effect on precision. Also, age of child/youth had no effect on any validity index, while in this analysis herein focused on adults, there were significant effects of speed, wear location, and age group on accuracy and bias (both *p* < 0.001) and precision (*p* ≤ 0.045). In regards with the effect of age group on accuracy and bias reported herein, we run exploratory analysis to test whether this effect persisted when including only the devices worn by all three age groups in the analysis, and the effect of age remained significant. These discrepant findings are tied, in part, to the fact that the specific wearable technologies tested have varied over the multiple years of original data collection tied to both the CADENCE-Kids [12] and CADENCE-Adults [6–8] studies due primarily to commercial availability. It is also possible that age effects (including decreased precision operationalized as increased variability) only became apparent with inclusion of the oldest adults in the study sample.

The success of this analysis is linked to several design strengths in the original study including the use of direct observation of step counting as the indisputable criterion measure [5], an intentionally broad range of treadmill walking speeds that included very slow speeds (starting at 0.8 km/h), the purposeful recruitment of a large sex- and age-balanced sample representing the adult lifespan, and the inclusion of a large number (21) of wearable technologies. One of the trade-offs necessary to test this large sample in such a standardized manner was the need to protract data collection over multiple years (2015 to 2019), and this pragmatic necessity was associated with the unavoidable and uncontrollable discontinuation of some commercial wearable technologies and the updating and/or emergence of others. In total, 21 wearable technologies were evaluated over the multi-year data collection period, but we must acknowledge that these represent only a proportion of the current and future market. Also, only a single device was evaluated at the ankle and one at the thigh, limiting the generalization to other devices that could also be worn at these locations. Some of the originally tested wearable technologies, such as Fitbit One, are also now obsolete. However, we consider the publication of validity values specific to these devices still important to enable strong comparisons between past, present, and future wearable technologies. Another limitation is that sample sizes naturally dwindled as progressively fewer individuals were able to achieve incrementally higher treadmill speeds. Related to this, and as noted earlier, the walk-to-run transition was highly individualized, and we ultimately chose to focus this present analysis on the much larger walking-based dataset to inform this catalog. Device performance during running remains an important point of consideration that is worthy of its own more focused examination which includes a larger sample selected for their fitness to complete a protocol that includes running stages. Importantly, this was a highly-controlled laboratory based study that is best positioned to informed standardized validity metrics [20]. Continued evaluation of wearable technologies under simulated activities of daily living and/or free-living conditions is warranted to understand device performance under the full range of settings and conditions.

## Conclusion

Standardized validation indices cataloged by speed, wear location, and age group across the adult lifespan facilitate selecting, evaluating, and/or comparing performance of step counting wearable technologies. Speed, wear location, and age had a significant effect on accuracy, bias, and precision. Overall, reduced performance was associated with slow walking speeds (0.8 to 3.2 km/h). Ankle- and thigh-located devices produced the highest accuracy, while those located at the wrist logged the worst accuracy. These results, along with the previously published children/youth catalog [9], provide an important foundation from which to build as new wearable technologies become available and can be evaluated using these same standardized approaches.

## Supplementary Information

**Additional file 1.** Table displaying step counting treadmill validation studies in adults and older adults.

**Additional file 2.** Visual and tabular presentations of the wearable technologies worn by the CADENCE-Adults participants.

**Additional file 3.** Tables displaying sample sizes, and number of steps derived by each treadmill speed for all sample and by age groups.

**Additional file 4.** Spreadsheets displaying the final analytical data set and the corresponding data dictionary.

**Additional file 5.** Catalog of validity indices for step counting wearable technologies at different speeds, wear locations, and age groups.

**Additional file 6.** Figures for descriptive Mean Percentage Error (MPE) representing bias of each wearable technology across walking speeds, and also presented by wear location and by age groups.

**Additional file 7.** Graphical representation of correlation coefficients (*r*) of the relationship between directly observed steps and steps derived from wearable technologies.

**Additional file 8.** Tabular description of validity indices by wear locations and by age groups.

**Additional file 9.** Figures of the effect of speed, wear location, and age on the overall precision of wearable technologies step counting ability.

**Additional file 10.** Figures of the effect of speed, wear location, and age on bias of wearable technologies step counting ability.

## Data Availability

All data generated or analyzed during this study (including a dataset) are included in this manuscript and its additional files.

## Abbreviations

BMI: Body mass index
CI: Confidence interval
CoV: Coefficient of variation
CTA: Consumer Technology Association
km/h: Kilometers per hour
MAPE: Mean absolute percentage error
mph: Miles per hour
MPE: Mean percentage error
mmHg: millimetres of mercury
SD: Standard deviation
