# Supplementary material for: A catalog of validity indices for step counting wearable technologies during treadmill walking: the CADENCE-adults study
Source: Int J Behav Nutr Phys Act. 2022 Sep 8;19:117. doi: 10.1186/s12966-022-01350-9 (PMC9461139; doi:10.1186/s12966-022-01350-9)
Supplement: Supplementary file 1 — Additional file 1. Table displaying step counting treadmill validation studies in adults and older adults. [file 12966_2022_1350_MOESM1_ESM.pdf]

Additional file 1: Suppl Table 1 Step counting treadmill validation studies among wearable technologies in adults and older adults

Reference (first author)	Sample	Protocol (duration; speeds)	Wearable Technologies (location)	Accuracy	Bias	Precision
Adamakis[1] 2021	30<br>25.9 ± 5.7<br>19-43<br>63%	5-min;<br>128.7, 160.9 m/min	Accupedo iOS: ACiwa (waist)<br>Pacer iOS: Piwa (waist)<br>Runtastic iOS: Riwa (waist)<br>Argus iOS: ARiwa (waist)<br>Accupedo Android: ACAwa (waist)<br>Pacer Android: PAwa (waist)<br>Runtastic Android: RAwa (waist)<br>Argus Android: ARAwa (waist)	MAPE:<br>ACiwa: 2.05%, 1.37%<br>Piwa: 2.05%, 1.37%<br>Riwa: 2.62%, 1.37%<br>ARiwa: 3.20%, 2.75%<br>ACAwa: 1.51%, 0.82%<br>PAwa: 1.56%, 0.82%<br>RAwa: 2.85%, 0.82%<br>ARAwa: 1.51%, 0.82%	Bland – Altman plots	Not reported
Ali[2] 2018	20<br>26 ± 3<br>18-65<br>85%	5-min;<br>53.4, 67.2, 79.8 m/min	3 Fitbit Flex: FFwr (wrist)	Not reported	MPE: -12%, -9%, -7%	ICC: 0.82, 0.78, 0.69
Alinia[3] 2017	15<br>21-31<br>47%	5-min;<br>41.7, 83.3, 133.3 m/min	Fitbit Zip: FZp (pant pocket)<br>Fitbit One: FOc (chest)<br>Fitbit Flex: FFwr (wrist)	MAPE:<br>FZp: 5.5%, 3.7%, 5.5%<br>FOc: 6.7%, 1.4%, 1.8%<br>FFwr: 6.8%, 3.9%, 4.4%	Not reported	ICC:<br>FZp: 0.91, 0.83, 0.71<br>FOc: 0.70, 1.37, 0.68<br>FFwr: 0.76, 1.07, 1.07
Clemes[4] 2010	68<br>19.2 ± 2.7<br>% not reported	3-min;<br>53.6, 67, 80.5, 93.9, 107.3 m/min	Silva: SLhr (hip; right)<br>Silva: SLhl (hip; left)<br>NL-1000: NLh (hip)	Not reported	MPE:<br>SLhr: 30.5-9.3%<br>SLhl: 46.9-6.7%<br>NLh: 12.9-0.9%	Not reported

DeCocker[5]<br>2012	40<br>29.5 ± 7.7<br>50%	5-min;<br>53.3, 66.7, 80, 93.3, 106.7 m/min	Omron HJ-203: OMp (pant pocket)<br>Omron HJ-203: OMb (carrier bag)<br>Omron HJ-203: OMn (neck)	MAPE:<br>OMp: 80%, 75%, 57.5%, 27.5%, 32.5%<br>OMc: 47.5%, 22.5%, 10%, 10%, 7.5%<br>OMn: 20%, 0%, 0%, 0%, 2.5%	MPE:<br>OMp: -15 – 65%<br>OMb: -5 – 20%<br>OMn: -2.5 – 17.5%	ICC: 0.25, 0.27, 0.53, 0.73, 0.96
Ducharme[6]<br>2021	75<br>29.57 ± 5.56<br>21-39<br>51%	5-min;<br>13.4, 26.8, 40.2, 53.6, 67, 80.5, 93.9, 107.3, 120.7 m/min	GENEActiv: GAwr (wrist)<br>GENEActiv: GAwa (waist)<br>ActiGraph GT9X: AGwr (wrist)<br>ActiGraph GT9X: AGwa (waist)	Not reported	Mean step error:<br>GAwr: -112.90 to 53.30<br>GAwa: -10.43 to -0.04<br>AGwr: -127.55 to 89.85<br>AGwa: -1.32 to 24.24	SD (±):<br>GAwr: 33.35 to 102.30<br>GAwa: 3.34 to 83.71<br>AGwr: 32.05 to 107.35<br>AGwa: 2.29 to 52.29
Feng[7]<br>2017	25<br>25.96 ± 7.86<br>18-53<br>44%	4-min;<br>54, 66, 78 m/min	APDM Opal: OPwa (waist)<br>Actigraph wGT3X-BT: AGwa (waist)<br>Axivity AX3: AXwa (waist)	Not reported	MPE:<br>OPwa: 0.292%<br>AGwa: -0.271%<br>AXwa: -0.089%	95% CI:<br>OPwa: (0.401-0.183)<br>AGwa: (-0.372 to -0.170)<br>AXwa: (-0.166 to -0.012)
Fokkema[8]<br>2017	31<br>32 ± 12<br>48%	10-min;<br>53.3, 80, 106.7 m/min	Polar Loop: PLwr (wrist)<br>Garmin Vivosmart: GVwr (wrist)<br>Fitbit Charge HR: FBwr (wrist)<br>Apple Watch Sport: AWwr (wrist)<br>Pebble Smartwatch: PSwr (wrist)<br>Samsung Gear S: SGwr (wrist)<br>Misfit Flash: MFh (hip)<br>Jawbone Up Move: JBh (hip)	Test 1<br>MAPE:<br>PLwr: 26.4%, 3.0%, 3.6%<br>GVwr: 1.0%, -0.2%, 9.0%<br>FBwr: -0.7%, 2.0%, 5.2%<br>AWwr: 1.9%, 0.0%, 0.5%<br>PSwr: 6.0%, 2.9%, 1.3%<br>SGwr: 5.6%, 4.0%, 1.1%	Bland-Altman plots	Test 1<br>ICC:<br>PLwr: 0.08, 0.26, 0.24<br>GVwr: 0.95, 0.57, 0.10<br>FBwr: 0.62, 0.20, 0.31<br>AWwr: 0.57, 0.93, 0.91<br>PSwr: 0.28, 0.34, 0.86,<br>SGwr: 0.04, 0.02, 0.85

			Flyfit: FFa (ankle)<br>Moves: MVp (pant pocket)	MFh: 15.2%, 5.4%, 6.0%<br>JBh: 8.7%, 5.9%, 1.2%<br>FFa: 16.1%, 5.3%, 2.3%<br>MVp: 14.0%, -2.6%, 0.3%<br><br>Test 2<br>MAPE:<br>PLwr: 26.3%, 10.7%, 3.0%<br>GVwr: 0.9%, 0.3%, 11.9%<br>FBwr: -0.9%, 1.7%, 7.7%<br>AWwr: 1.4%, 2.6%, 0.1%<br>PSwr: 3.0%, 1.4%, 1.0%<br>SGwr: 4.8%, 3.5%, 0.8%<br>MFh: 18.1%, 8.5%, 7.5%<br>JBh: 11.7%, 2.6%, 1.6%<br>FFa: 19.5%, 7.0%, 6.8%<br>MVp: 12.6%, -0.8%, -0.2%		MFh: 0.06, 0.26, 0.11<br>JBh: 0.12, 0.09, 0.71<br>FFa: 0.18, 0.31, 0.27<br>MVp: 0.15, 0, 0.25<br><br>Test 2<br>ICC:<br>PLwr: 0.09, 0, 0.42<br>GVwr: 0.95, 0.95, 0<br>FBwr: 0.74, 0.27, 0.15<br>AWwr: 0.73, 0.52, 0.86<br>PSwr: 0.78, 0.77, 0.91<br>SGwr: 0, 0.17, 0.92<br>MFh: 0.15, 0.07, 0.08<br>JBh: 0.17, 0.56, 0.72<br>FFa: 0.17, 0.32, 0.08<br>MVp: 0, 0, 0.37
Foster[9]<br>2005	10 (lean)<br>10 (obese)<br>30 ± 13<br>32 ± 7<br>21-51<br>50%	15-min;<br>16.7, 33.3,<br>50 m/min	Omron HF-100: OMkl (knee; lean)<br>Omron HF-100: OMko (knee; obese)<br>Digi-Walker 2: DWkl (knee; lean)<br>Digi-Walker 2: DWko (knee; obese)<br>Stepwatch: SWal (ankle; lean)<br>Stepwatch: SWao (ankle; obese)	Not reported	MPE:<br>OMkl: 56%, 98%, 99%<br>OMko: 66%, 94%, 97%<br>DWkl: 36%, 89%, 100%<br>DWko: 17%, 76%, 100%<br>SWal: 99%, 100%, 100%<br>SWao: 100%, 100%, 100%	SD:<br>OMkl: 14, 2.9, 2.1<br>OMko: 10, 3.5, 2.2<br>DWkl: 11, 4.7, 2.6<br>DWko: 7, 7.3, 2.7<br>SWal: 0.56, 0.34, 0.30<br>SWao: 0.35, 0.26, 0.37
Grant[10]<br>2008	21<br>65-87	5-min;	ActivPAL: APt (thigh)	MAPE:<br>only visualized in graph	Bland-Altman plots<br>Mean difference:	SD:<br>437 ± 56

	52%	40.2, 54, 67.2, 79.8, 93.6 m/min	Digi-Walker SW-200: DWwa (waist)<br>NL-2000: NLwa (waist)		APt: 2.6, 0.6, -0.1, 0.4, 0.4<br>SWwa: 184.3, 132.7, 71.8, 31.2, 4<br>NLwa: 85.4, 4.8, 0, -0.9, -2.4	490 ± 55<br>532 ± 47<br>585 ± 47<br>624 ± 43
Han[11]<br>2020	36<br>21.2 ± 4.5<br>18-65<br>50%	2-min;<br>53.3, 66.7, 80, 93.3, 106.7 m/min	Garmin Forerunner 235: GMwr (wrist)<br>Polar M430: PLwr (wrist)<br>Garmin Foot Pod: FPf (foot)<br>Polar Stride Sensor Smart: SSf (foot)	MAPE:<br>GMwr: 14.3%, 4.2%, 1.2%, 0.9%, 1.0%<br>PLwr: 9.9%, 7.0%, 3.1%, 1.5%, 0.5%<br>FPf: 0.4%, 0.5%, 0.5%, 0.5%, 0.6%<br>SSf: 0.3%, 0.4%, 0.3%, 0.3%, 0.2%	Bland-Altman plots	CCI:<br>GMwr: 0.919<br>PLwr: 0.908<br>FPf: 0.999<br>SSf: 1.000
Horvath[12]<br>2007	20<br>27.8 ± 8.8<br>45%	50 steps;<br>54, 80, 107 m/min	Yamax SW200: DWlm (left mid-axillary)<br>Yamax SW200: DWrm (right mid-axillary)<br>Yamax SW200: DWrt (right thigh)<br>Yamax SW200: DWlt (left thigh)<br>Yamax SW200: DWwa (waist)	Not reported	Net error:<br>DWlm: 7.3%, 0.6%, -1.2%<br>DWrm: 10.5%, 2.1%, -0.5%<br>DWrt: 18.1%, 3.1%, -0.2%<br>DWlt: 10.3%, 1.9%, 0.6%<br>DWwa: 17.7%, 0.4%, -0.8%	Not reported
John[13]<br>2018	20<br>26.7 ± 4.9<br>40%	1-min;<br>53.4, 58.7, 64.1, 69.4, 74.8, 80.1, 85.4, 90.8,	Yamax Digiwalker SW200: DWwa (waist)<br>OmronHJ720ITC: OMwa (waist)<br>ActiGraph GT3X+: 3Xwa (waist)<br>ActiGraph GT3X+: 3Xwr (wrist)	Not reported	Bias:<br>DWwa: 1 – 21<br>OMwa: -5 – 6<br>3Xwa: 8 – 39<br>3Xwr: 32 – 46	SD:<br>DWwa: 10-34<br>OMwa: 9-31<br>3Xwa: 7-19<br>3Xwr: 15-26

		96.1, 101.5, 106.8 m/min	ActiGraph GT9X: 9Xwa (waist)<br>ActiGraph GT9X: 9Xwr (wrist)		9Xwa: 55 – 64<br>9Xwr: 66 – 72	9Xwa: 19-28<br>9Xwr: 11-18
Jung[14]<br>2020	32<br>26.03 ± 6.59<br>18-40<br>53%	5-min;<br>53.6, 107.3 m/min	Fitbit HR: FBrwrp (right wrist proximal)<br>Fitbit HR: FBrwrd (right wrist distal)<br>Fitbit HR: FBlwrp (left wrist proximal)<br>Fitbit HR: FBlwrd (left wrist distal)	Not reported	Mean difference:<br>FBrwrp: -13.47, 19.84<br>FBrwrd: -12.22, -6.06<br>FBlwrp: -20.63, 12.59<br>FBlwrd: -12.63, -2.72	95% CI:<br>FBrwrp: (-25.39, -1.55), (-9.05, 48.74)<br>FBrwrd: (-25.21, 0.78), (-31.48, 19.36)<br>FBlwrp: (-37.8, -3.45), (-21.49, 46.68)<br>FBlwrd: (-23.7, -1.55), (-25.67, 20.24)
Karaca[15]<br>2021	29<br>26.3 ± 6.2<br>18-40<br>0%	2-min;<br>33.3, 66.7, 100 m/min	ActiGraph wGT3X-BT: AGrwr (right wrist)<br>ActiGraph wGT3X-BT: AGlwr (left wrist)<br>ActiGraph wGT3X-BT: AGwa (waist)<br>ActiGraph wGT3X-BT: AGra (right ankle)<br>ActiGraph wGT3X-BT: AGua (upper arm)	MAPE:<br>AGrwr: 41.7%, 16.3%, 25.1%<br>AGlwr: 32.3%, 26.5%, 28.3%<br>AGwa: 80.0%, 8.3%, 1.2%<br>AGra: 12.4%, 1.0%, 4.9%<br>AGua: 66.5%, 11.7%, 3.3%	Bland-Altman plots:<br>AGrwr: -62.3, -32.7, -64.5<br>AGlwr: -48.6, -54.2, -97<br>AGwa: -120.9, -15.5, 2<br>AGra: -12.2, 1, -12.7<br>AGua: -99.3, -23.2, -5.2	SD:<br>AGrwr: 48.2-31.5<br>AGlwr: 51.8-37.3<br>AGwa: 34.6-4.5<br>AGra: 39.4-3.6<br>AGua: 27.5-18.7
Lowe[16]<br>2021	18<br>30.6<br>18-65<br>56%	5-min;<br>53.6, 80.5, 107.3 m/min	Walk Star: iPhone 5: WSp (pant pocket)<br>Accupedo: Samsung S3 mini: SSp (pant pocket)	Not reported	Bland-Altman plots could not be extricated for treadmill	ICC:<br>WSp: 0.145-0.951<br>SSp: -0.400-0.544<br>DWwa: 0.027-0.383<br>OMwa: 0.631-0.977

			Yamax Digi-Walker CW-700: DWwa (waist)<br>Omron HJ-720ITC: OMwa (waist)<br>Tanita PD-724: TAn (neck)			TAn: 0.391-0.866
Nuss[17]<br>2020	30<br>22.93 ± 3.3<br>50%	7-min;<br>45.6, 67, 91.1, 112.6, 134.1, 147.5, 160.9 m/min	ActiGraph: AGrwr (right wrist)<br>ActiGraph: AGlwr (left wrist)<br>ActiGraph: AGrh (right hip)<br>ActiGraph: AGlh (left hip)	MAPE:<br>AGrwr: 50.31-31.33%<br>AGlwr: 50.35-36.84%<br>AGrh: 54.52-1.55%<br>AGlh: 59.97-1.55%	Not reported	CCC:<br>AGrwr: -0.008 – 0.1<br>AGlwr: -0.006 – 0.08<br>AGrh: -0.004 – 0.99<br>AGlh: -0.002 – 0.92
O'Brien [18]<br>2018	43<br>39.4 ± 15.2<br>20-64<br>58%	6-min;<br>40, 53.3, 66.7, 93.3, 106.7, 120 m/min	Omron HJ-320: OMwa (waist)<br>ActiGraph GT3X: AGwa (waist)<br>PiezoRx: PRwa (waist)	Not reported	Bland-Altman plots	ICC:<br>OMwa: 0.14, 0.28, 0.94, 0.39, 0.53, 0.66, 0.62<br>AGwa: 0.16, 0.22, 0.70, 0.96, 0.93, 0.98, 0.72<br>PRwa: 0.71, 0.80, 0.98, 0.99, 0.95, 0.99, 0.97
O'Brien[19]<br>2021	19<br>68.8 ± 2.3<br>37%	6-min;<br>40, 53.3, 93.3, 106.7 m/min	Omron HJ-320: OMwa (waist)<br>ActiGraph GT3X: AGwa (waist)<br>PiezoRx: PRwa (waist)	MAPE:<br>OMwa: 78.3 - 1.5%<br>AGwa: 72.9 - 1.4%<br>PRwa: 8.4 - 1.2%	Bland-Altman plots	ICC:<br>OMwa: 0.26, 0.36, 0.85, 0.92, 0.91<br>AGwa: -0.13, -0.20, 0.30, 0.94, 0.71<br>PRwa: 0.18, 0.98, 0.96, 0.92, 0.90
Reil[20]<br>2016	30<br>27.9 ± 4.2	2-min;	ActiGraph wGT3X-BT: AGwa (waist)	Not recorded	Bland-Altman plots<br>MPE:	ICC:<br>AGwa: 0.03, 0.55, 0.64

	50%	53.3, 80, 106.7 m/min	Mother: MOwa (waist)		AGwa: 26.7%, 3.7%, 2.8%<br>MOwa: 2.5%, 1.3%, 1.9%	MOwa: 0.88, 0.96, 0.89
Rhudy[21]<br>2018	15<br>20.2 ± 3.8<br>18.2-32.9<br>% not reported	1.5-min;<br>72 m/min	gyroscope: PEAKwr (wrist)<br>AC - setting: ACwr (wrist)<br>FFT - setting: FFTwr (wrist)<br>gyroscope: PEAKa (ankle)<br>AC - setting: ACa (ankle)<br>FFT - setting: FFTa (ankle)	Not reported	Bland-Altman plots	ICC:<br>PEAKwr: 0.640<br>ACwr: 0.962<br>FFTwr: not reported<br>PEAKa: 0.991<br>ACa: 0.926<br>FFTa: 0.985
Simonsen[22]<br>2020	30<br>28.20 ± 4.33<br>13%	3-min;<br>33.3, 50, 66.7 m/min	Polar M200: PMf (forearm)<br>Polar A300: PAf (forearm)<br>Samsung Galaxy S9: SSp (pant pocket)<br>Dunlop Sport: DSwa (waist)	Not reported	Bland-Altman plots<br>SES:<br>PMf: 20.39, 14.15, 19.87<br>PAf: 22.43, 14.28, 19.78<br>SSp: 9.17, 9.41, 15.94<br>DSwa: 22.40, 14.16, 19.36	Correlation coefficient:<br>PMf: -0.19, 0.29, 0.23<br>PAf: -0.23, 0.24, 0.13<br>SSp: 0.52, 0.86, 0.76<br>DSwa: 0.10, 0.22, 0.32
Svarre[23]<br>2020	30<br>26.6 ± 6.2<br>18-64<br>60%	5-min;<br>26.7, 40, 53.3, 66.7, 80, 93.3 m/min	Garmin Vivosmart HR: GVwr (wrist)<br>StepWatch 3: SWa (ankle)	MAPE:<br>GVwr: 26.35%, 3.49%, 1.27%, 0.61%, 0.61%, 6.45%<br>SWa: 3.60%, 0.35%, 0.09%, 0.08%, 0.08%, 0.09%	Bland-Altman plots	SD (±):<br>GVwr: 23.63, 4.10, 1.67, 0.61, 0.68, 11.46<br>SWa: 6.03, 1.00, 0.12, 0.09, 0.10, 0.12
Swartz[24]<br>2003	66<br>28.6 ± 10<br>47%	3-min;	Yamax SW-200: DWwaf (waist; front)	Not reported	MPE: visualized only	Not reported

		54, 67, 80, 94, 107 m/min	Yamax SW-200: DWwab (waist; back)<br>Yamax SW-200: DWwas (waist; side)			
Tedesco[25]<br>2019	18<br>65-74<br>61%	3-min;<br>16.7, 25, 33.3 m/min	Fitbit Charge 2: FCD (D wrist)<br>Fitbit Charge 2: FCND (ND wrist)<br>Garmin VivoSmart HR+: GVD (D wrist)<br>Garmin VivoSmart HR+: GVND (ND wrist)<br>Philips Health Watch: PHD (D wrist)<br>Philips Health Watch: PHND (ND wrist)<br>Withings Pulse Ox: WPD (D wrist)<br>Withings Pulse Ox: WPND (ND wrist)<br>Omron HJ-72OITC: OM (waist)<br>ActiGraph GT9X-BT: AGW (waist)<br>ActiGraph GT9X-BT: AGA (ankle)<br><br>D = dominant<br>ND = non-dominant	MAPE:<br>FCD: 44.53%, 29.31%, 27.65%<br>FCND: 44.86%, 28.33%, 26.50%<br>GVD: 54.74%, 22.32%, 7.78%<br>GVND: 53.01%, 25.66%, 9.99%<br>PHD: 51.30%, 28.39%, 20.38%<br>PHND: 44.76%, 20.88%, 11.24%<br>WPD: 86.07%, 76.20%, 59.90%<br>WPND: 86.98%, 81.55%, 57.45%<br>OM: 77.60%, 58.92%, 32.76%<br>AGW: 87.05%, 77.20%, 62.99%<br>AGA: 51.20%, 31.78%, 8.87%	Bland - Altman plots:<br>FCD: -101.06, 20.5, -181.18<br>FCND: -91.35, 19.5, 19.19<br>GVD: -118.29, -50.71, -18.63<br>GVND: -111.41, -58.53, -19.75<br>PHD: -110, -44.56, 8.93<br>PHND: -82.65, -29.19, 6.31<br>WPD: -200.41, -181.18, -155.31<br>WPND: -201.59, -188.59, -144.56<br>OM: -177.76, -140.59, -84.94<br>AGW: -207.91, -183.65, -159.63<br>AGA: -107.85, -69.21, -19.47	ICC:<br>FCD: 0.44, 0.30, 0.13<br>FCND: 0.44, 0.41, 0.12<br>GVD: 0.36, 0.64, 0.91<br>GVND: 0.46, 0.58, 0.79<br>PHD: 0.41, 0.44, 0.47<br>PHND: 0.45, 0.44, 0.63<br>WPD: 0.22, 0.13, 0.17<br>WPND: 0.24, 0.18, 0.26<br>OM: 0.24, 0.22, 0.23<br>AGW: 0.11, 0.17, 0.18<br>AGA: 0.51, 0.52, 0.83

Tophoj[26] 2018	20<br>25.6 ± 2<br>50%	100-steps; 33.3, 66.7, 75.0, 91.7 m/min	Fitbit Surge: FSwr (wrist)<br>Fitbit Charge HR: FCwr (wrist)<br>Microsoft Band 2: MBwr (wrist)<br>A&D Activity Monitor: ADh (hip)	Not reported	Bland – Altman plots<br>MPE:<br>FSwr: -8%, -2%, -2%, -6%<br>FCwr: 15%, 18%, 21%, 7%<br>MBwr: -15%, 0%, 0%, 0%<br>ADh: -53%, 1%, 2%, 2%	ICC:<br>FSwr: 0.58<br>FCwr: 0.35<br>MBwr: 0.79<br>ADh: 0.96<br>SD:<br>FSwr: 23, 9, 8, 13<br>FCwr: 56, 50, 36, 36<br>MBwr: 46, 4, 3, 3<br>ADh: 42, 4, 3, 2
Toth[27] 2017	25<br>26 ± 8<br>18-57<br>% not reported	2-min; 26.8, 53.6, 80.5, 107.2, 134.1, 160.9 m/min	StepWatch 3: SWac100 (ankle; cadence setting 100%)<br>StepWatch 3: SWac83 (ankle; cadence setting 83%)<br>StepWatch 3: SWac70 (ankle; cadence setting 70%)<br>StepWatch 3: SWac60 (ankle; cadence setting 60%)<br>StepWatch 3: SWas18 (ankle; sensitivity 18)<br>StepWatch 3: SWas16: (ankle; sensitivity 16)<br>StepWatch 3: SWas14: (ankle; sensitivity 14)<br>StepWatch 3: SWas13: (ankle; sensitivity 13)<br>StepWatch 3: SWas12: (ankle; sensitivity 12)	Not reported	Percentage of Criterion Steps:<br>SWac100: 108.9%, 99.6%, 99.6%, 99.4%, 75.5%, 70.5%<br>SWac83: 124.0%, 101.6%, 99.6%, 99.7%, 98.0%, 95.9%<br>SWac70: 130.5%, 121.6%, 108.8%, 101.0%, 99.6%, 99.4%<br>SWac60: 149.5%, 149.8%, 119.3%, 108.4%, 101.0%, 100.5%<br>SWas18: 106.8%, 100.3%, 100.2%, 99.6%, 98.1%, 99.2%	Not reported

					SWas16: 110.5%, 103.4%, 104.4%, 99.6%, 99.1%, 99.7%<br>SWas14: 126.3%, 115.0%, 106.0%, 99.7%, 99.7%, 99.6%<br>SWas13: 130.5%, 121.6%, 108.8%, 101.0%, 99.6%, 99.4%<br>SWas12: 135.8%, 125.2%, 111.2%, 101.4%, 100.2%, 99.5%	
Vetrovsky[28]<br>2019	20<br>34.3 ± 11.6<br>25%	3-min;<br>40, 50, 60, 70 m/min	Fitbit Charge 2: FCwr (wrist)<br>Garmin vivofit 1: G1wr (wrist)<br>Garmin vivofit 3: Gwr3 (wrist)<br>Omron HJ-322U-E: OMwa (waist)<br>SmartLAB walk+: SLn (neck)	MAPE:<br>FBwr: 10%, 4%, 1%, 2%<br>G1wr: 24%, 7%, 3%, 2%<br>G3wr: 13%, 8%, 1%, 1%<br>OMwa: 45%, 7%, 1%, 1%<br>SLn: 18%, 4%, 1%, 1%	MPE:<br>FBwr: 3%, 0%, -1%, -2%<br>G1wr: -24%, -7%, -2%, -2%<br>G3wr: 4%, 4%, -1%, -1%<br>OMwa: -44%, -7%, -1%, -1%<br>SLn: -18%, -3%, 0%, 0%	CCC:<br>FBwr: 0.38, 0.82, 0.99, 0.90<br>G1wr: 0.11, 0.66, 0.95, 0.96<br>G3wr: 0.45, 0.46, 0.99, 0.97<br>OMwa: 0.11, 0.21, 0.99, 0.99<br>SLn: 0.22, 0.59, 0.99, 0.98

CCC = concordance correlation coefficients; CCI = correlation confidence interval; CI = confidence interval; ICC = intra-class coefficient; MAPE = mean absolute percentage error; MPE = mean percentage error; PE = percent error; SES = standard error of the estimate; SD = standard deviation.

Population descriptions are presented as means ± standard deviation, year ranges, and percent females. Speeds were converted from mph, km/h, or m/s into m/min if necessary.

Values are presented for each speed for each device. Inconsistencies across the rows in the table are due to discrepancies in the article reports.

Search was performed using an expanded Boolean string of (((step*) AND (treadmill*)) AND (adult*)) AND ((valid*) OR (reliab*)) in search engines PubMed, SPORTDiscuss, Web of Science, and CINHAL. Studies were included if the validation of step-counting devices was performed on a treadmill at walking speeds using the criterion standard of direct observation in ostensibly healthy adults. The exclusion criteria were: populations under the age of 18 years, indistinguishably combined step data for children/youth and adults, incline > 0% grade, non-standardized treadmill speed, unspecified walking/jogging/running bouts, overground/stair/track walking, and steps not reported as an outcome.

References

1. Adamakis M. Criterion Validity of iOS and Android Applications to Measure Steps and Distance in Adults. *TECHNOLOGIES.* SEP 2021;9(3).
2. Ali E, Wani D, Ling W, Rao S. Reliability and validity of wrist-worn activity monitors in healthy young adults. *Physiotherapy Practice & Research.* 2018;39(2):117-124.
3. Alinia P, Cain C, Fallahzadeh R, Shahrokni A, Cook D, Ghasemzadeh H. How Accurate Is Your Activity Tracker? A Comparative Study of Step Counts in Low-Intensity Physical Activities. *JMIR Mhealth Uhealth.* Aug 11 2017;5(8):e106.
4. Clemes SA, O'Connell S, Rogan LM, Griffiths PL. Evaluation of a commercially available pedometer used to promote physical activity as part of a national programme. *British Journal of Sports Medicine.* Dec 2010;44(16):1178-1183.
5. De Cocker KA, De Meyer J, De Bourdeaudhuij IM, Cardon GM. Non-traditional wearing positions of pedometers: Validity and reliability of the Omron HJ-203-ED pedometer under controlled and free-living conditions. *Journal of Science and Medicine in Sport.* Sep 2012;15(5):418-424.
6. Ducharme SW, Jongil L, Busa MA, et al. A Transparent Method for Step Detection Using an Acceleration Threshold. *Journal for the Measurement of Physical Behaviour.* 2021;4(4):311-320.
7. Feng Y, Wong CK, Janeja V, Kuber R, Mentis HM. Comparison of tri-axial accelerometers step-count accuracy in slow walking conditions. *Gait & Posture.* 2017:11-16.
8. Fokkema T, Kooiman TJ, Krijnen WP, CP VDS, M DEG. Reliability and Validity of Ten Consumer Activity Trackers Depend on Walking Speed. *Med Sci Sports Exerc.* Apr 2017;49(4):793-800.

9. Foster RC, Lanningham-Foster LM, Manohar C, et al. Precision and accuracy of an ankle-worn accelerometer-based pedometer in step counting and energy expenditure. *Prev Med.* Sep-Oct 2005;41(3-4):778-783.

10. Grant PM, Dall PM, Mitchell SL, Granat MH. Activity-monitor accuracy in measuring step number and cadence in community-dwelling older adults. *Journal of Aging and Physical Activity.* Apr 2008;16(2):201-214.

11. Han H, Kim H, Sun W, Malaska M, Miller B. Validation of wearable activity monitors for real-time cadence. *Journal of Sports Sciences.* Feb 2020;38(4):383-389.

12. Horvath S, Taylor DG, Marsh JP, Kriellaars DJ. The effect of pedometer position and normal gait asymmetry on step count accuracy. *Applied Physiology, Nutrition & Metabolism.* 2007;32(3):409-415.

13. John D, Morton A, Arguello D, Lyden K, Bassett D. "What Is a Step?" Differences in How a Step Is Detected among Three Popular Activity Monitors That Have Impacted Physical Activity Research. *Sensors.* Apr 2018;18(4).

14. Jung HC, Kang M, Lee NH, Jeon S, Lee S. Impact of Placement of Fitbit HR under Laboratory and Free-Living Conditions. *Sustainability.* Aug 2020;12(16).

15. Karaca A, Demirci N, Yilmaz V, Aytar SH, Can S, Unver E. Validation of the ActiGraph wGT3X-BT Accelerometer for Step Counts at Five Different Body Locations in Laboratory Settings. *Measurement in Physical Education and Exercise Science.*

16. Lowe CJM, Isaac C, Kelly P, Barker KL. Measuring step count: why it is important not to assume measures are reliable. *Physiotherapy.* Mar 2021;110:1-4.

17. Nuss KJ, Hulett NA, Erickson A, et al. Comparison of Energy Expenditure and Step Count Measured by ActiGraph Accelerometers Among Dominant and Nondominant Wrist and Hip Sites. *Journal for the Measurement of Physical Behaviour.* 2020;3(4):315-322.

18. O'Brien MW, Wojcik WR, Fowles JR. Medical-Grade Physical Activity Monitoring for Measuring Step Count and Moderate-to-Vigorous Physical Activity: Validity and Reliability Study. *JMIR Mhealth Uhealth.* Sep 5 2018;6(9):e10706.

19. O'Brien MW, Wojcik WR, Fowles JR. Validity and Interinstrument Reliability of a Medical Grade Physical Activity Monitor in Older Adults. *Journal for the Measurement of Physical Behaviour*. 2021;4(1):31-38.

20. Riel H, Rathleff CR, Kalstrup PM, et al. Comparison between Mother, ActiGraph wGT3X-BT, and a hand tally for measuring steps at various walking speeds under controlled conditions. *PeerJ.* 2016;4:e2799.

21. Rhudy MB, Mahoney JM. A comprehensive comparison of simple step counting techniques using wrist- and ankle-mounted accelerometer and gyroscope signals. *Journal of Medical Engineering & Technology.* 2018;42(3):236-243.

22. Simonsen MB, Thomsen MJ, Hirata RP. Validation of different stepping counters during treadmill and over ground walking. *Gait Posture.* Jul 2020;80:80-83.

23. Svarre FR, Jensen MM, Nielsen J, Villumsen M. The validity of activity trackers is affected by walking speed: the criterion validity of Garmin Vivosmart(®) HR and StepWatch(™) 3 for measuring steps at various walking speeds under controlled conditions. *PeerJ.* 2020;8:e9381.

24. Swartz AM, Bassett DR, Moore JB, Thompson DL, Strath SJ. Effects of body mass index on the accuracy of an electronic pedometer. *International Journal of Sports Medicine.* Nov 2003;24(8):588-592.

25. Tedesco S, Sica M, Ancillao A, Timmons S, Barton J, O'Flynn B. Accuracy of consumer-level and research-grade activity trackers in ambulatory settings in older adults. *PLoS One.* May 2019;14(5).

26. Tophøj KH, Petersen MG, Sæbye C, Baad-Hansen T, Wagner S. Validity and Reliability Evaluation of Four Commercial Activity Trackers' Step Counting Performance. *Telemed J E Health.* Sep 2018;24(9):669-677.

27. Toth LP, Bassett DR, Crouter SE, et al. StepWatch accuracy during walking, running, and intermittent activities. *Gait & Posture.* Feb 2017;52:165-170.

28. Vetrovsky T, Siranec M, Marencakova J, et al. Validity of six consumer-level activity monitors for measuring steps in patients with chronic heart failure. *PLoS One.* 2019;14(9):e0222569.
