# Supplementary material for: A catalog of validity indices for step counting wearable technologies during treadmill walking: the CADENCE-adults study
Source: Int J Behav Nutr Phys Act. 2022 Sep 8;19:117. doi: 10.1186/s12966-022-01350-9 (PMC9461139; doi:10.1186/s12966-022-01350-9)
Supplement: Supplementary file 2 — Additional file 2. Visual and tabular presentations of the wearable technologies worn by the CADENCE-Adults participants. [file 12966_2022_1350_MOESM2_ESM.pdf]

1
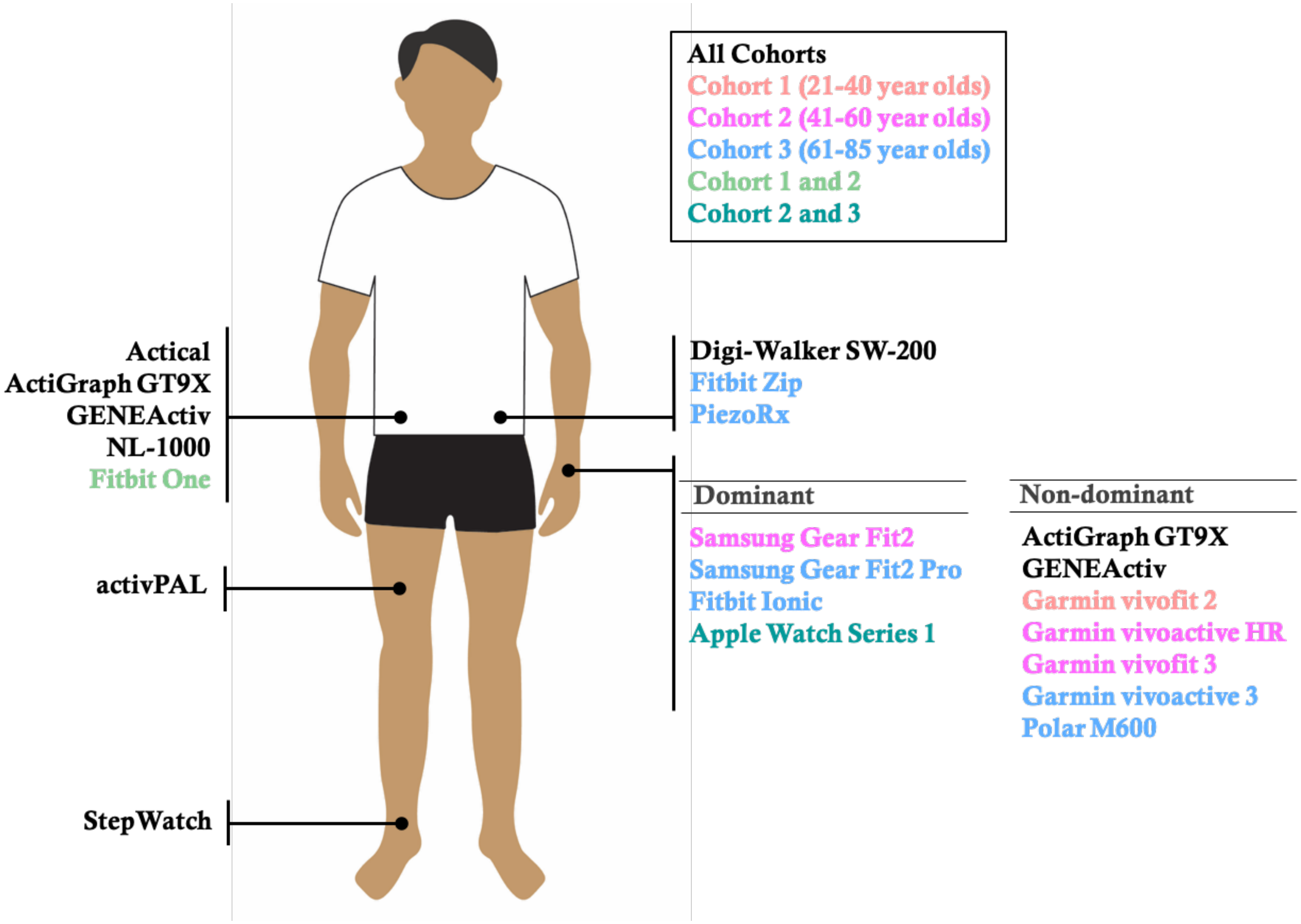

2 **Additional file 2: Suppl Fig 1** Visual representation of all wearable technologies and their locations by age cohorts

Additional file 2: Suppl Table 1. Relation of wearable technologies used in the present study

Wearable	Location	Digital Display	Data Extraction Software	Initial Step Data Resolution (Epoch)	Device Settings and Initialization Information	Commercial availability
Actical	Right waist	No	Actical®<br>(v. 2.12.0002)	1 s	• Set "*Epoch length*" to 1 s.<br>• "*Record Steps?*" box checked but not the "*Raw Data*" check box.	Available
ActiGraph GT9X	Right waist and non-dominant wrist	No	ActiLife<br>(v. 6.11.8)	1 s	• Sampling rate: 80 Hz.<br>• LED flashes not checked.<br>• "*Idle sleep mode*" disabled.<br>• "*Low Frequency Extension*" disabled.<br>• "*Enable Wireless*" option should be disabled.<br>• "*Enable IMU*" unchecked.<br>• *"By default"* algorithm for step counting analysis.<br>• When downloading, make sure the *"# of Axis:*" reads *3*. Check "*Steps*" box but not "*Lux*", "*Inclinometer*", or "*Low Frequency Extension*" boxes.	Available
activPAL	Right thigh	No	activPAL™ process and presentation<br>(v. 6.4.1)	15 s	• *"Future Time"* selected for Start condition.<br>• "*Record for 1 day*" selected.	Available
Apple Watch Series 1	Dominant wrist	Yes	N/A	N/A	• Go to *"Activity"* and press *"Get Started"*.<br>• Turn the Digital Crown to see the totals. Keep scrolling to see total steps.	Updated to Apple Watch Series 7
Digi-Walker SW-200	Left waist	Yes	N/A	N/A	• Press "RESET" button to clear the currently stored "STEP" value.	Available

Wearable	Location	Digital Display	Data Extraction Software	Initial Step Data Resolution (Epoch)	Device Settings and Initialization Information	Commercial availability
Fitbit Ionic	Dominant wrist	Yes	N/A	N/A	• After the device is placed on the location, values will be recorded.<br>• Swipe up from the clock face on the watch to access "Fitbit Today" where the steps are prompted.	Discontinued
Fitbit One	Right waist	Yes	N/A	N/A	• After the device is placed on the location, values will be recorded.<br>• The value of steps and icon is displayed immediately after pressing the tracker's button for faster viewing.<br>• Before starting the test, the tracker is reset.	Updated to FitBit Inspire 2
Fitbit Zip	Left waist	Yes	N/A	N/A	• After the device is placed on the location, values will be recorded.<br>• Zip will track and display the steps taken	Discontinued
Garmin vivoactive 3	Non-dominant wrist	Yes	N/A	N/A	• The device comes preloaded with widgets that provide at-a glance information. The number of steps taken appears on the steps widget.<br>• Press the "Key" button.<br>• Select *"Walk"* from all possible activities.<br>• Press *"Ready"* button and steps will be displayed on the screen.	Updated to Garmin vivoactive 4
Garmin vivoactive HR	Non-dominant wrist	Yes	N/A	N/A	• The device comes preloaded with widgets that provide at-a glance	Available

Wearable	Location	Digital Display	Data Extraction Software	Initial Step Data Resolution (Epoch)	Device Settings and Initialization Information	Commercial availability
					information. The number of steps taken appears on the steps widget.<br>• Press the "Key" button.<br>• Select *"Walk"* from all possible activities.<br>• Press *"Ready"* button and steps will be displayed on the screen.	
Garmin vivofit 2	Non-dominant wrist	Yes	N/A	N/A	• Hold the device key until "*Start*" appears.<br>• The value of steps and icon is displayed immediately after starting a new activity.	Updated to Garmin vivofit 4
Garmin vivofit 3	Non-dominant wrist	Yes	N/A	N/A	• Hold the device key until "*Start"* appears. The timer begins.<br>• The value of steps and icon is displayed immediately after starting a new activity.	Updated to Garmin vivofit 4
GENEActiv	Right waist and non-dominant wrist	No	GENEActiv PC Software	1 s	• *"Measurement frequency"*: 60 Hz.<br>• *"Measurement Period"*: 1 day 00 hours.<br>• *"Recording Start Mode"*: At Future Time. Set the start date and time at the time subject orientation is scheduled to begin.<br>• Enter the "*Body Location*" where the device will be used (right waist or non-dominant wrist).<br>• Select the check box next to the device(s) you wish to initialize.	Available

Wearable	Location	Digital Display	Data Extraction Software	Initial Step Data Resolution (Epoch)	Device Settings and Initialization Information	Commercial availability
					• Click "*Erase & Configure*" to initialize the device.<br>• To download data, click on the "*Data Extractor*" tab.	
New Lifestyles NL-1000	Right waist	Yes	N/A	N/A	• Mode button pushed to display "STEPS".	Available
PiezoRx	Left waist	Yes	N/A	N/A	• Open the pedometer's front cover, reset the device by pressing and holding the *"X"* button for a few seconds to reset the step count.<br>• Open the pedometer's front cover and record the step value from the display indicated by the she in the top left corner.	Discontinued
Polar M600	Non-dominant wrist	Yes	N/A	N/A	• Press the side power button to open the "*Apps*" menu, and find and tap *"Polar"*. The "*Polar"* app on M600 tracks activity with a 3D accelerometer that records wrist movements.<br>• Click on the *"24/7 Activity tracking"* feature. This feature is always on and displays the steps progress on the home screen.	Available
Samsung Gear Fit2	Dominant wrist	Yes	N/A	N/A	• Turn on the device and the home screen will display the number of steps taken.<br>• Tap *"Steps"* on the *"Apps"* page.	Discontinued

Wearable	Location	Digital Display	Data Extraction Software	Initial Step Data Resolution (Epoch)	Device Settings and Initialization Information	Commercial availability
					• The current step total and target step number will be displayed.	
Samsung Gear Fit2 Pro	Dominant wrist	Yes	N/A	N/A	• Turn on the device and the home screen will display the number of steps taken.<br>• Tap *"Steps"* on the *"Apps"* page.<br>• The current step total and target step number will be displayed.	Discontinued
StepWatch	Right ankle	Yes	StepWatch™ Analysis Software (v. 3.1)	60 s	• Recording Interval of "*3 seconds*" selected.<br>• Select *"Later*" for the "Start recording steps" option.<br>• Select the last option "*Other*" and manually enter "*1 day"* for the "Stop recording after" option.<br>• "*Set to Max*" button selected.	Updated to StepWatch 4

Devices with "N/A" listed for Data Extraction Software and Initial Step Data Resolution (Epoch) were standalone devices which did not require interfacing with a computer for data extraction – data read directly from available visual displays at the end of each 5-minute stage
