# Supplementary material for: A catalog of validity indices for step counting wearable technologies during treadmill walking: the CADENCE-adults study
Source: Int J Behav Nutr Phys Act. 2022 Sep 8;19:117. doi: 10.1186/s12966-022-01350-9 (PMC9461139; doi:10.1186/s12966-022-01350-9)
Supplement: Supplementary file 3 — Additional file 3. Tables displaying sample sizes, and number of steps derived by each treadmill speed for all sample and by age groups. [file 12966_2022_1350_MOESM3_ESM.pdf]

Additional File 3: Suppl Table 1 Sample sizes (*n*) and number of steps derived from direct observation and each wearable technology at each treadmill speed for all sample (21–85 years)

	**Treadmill speed, km/h (mph)**									
	**Slow**				**Normal**				**Fast**	
	**0.8 (0.5)**	**1.6 (1.0)**	**2.4 (1.5)**	**3.2 (2.0)**	**4.0 (2.5)**	**4.8 (3.0)**	**5.6 (3.5)**	**6.4 (4.0)**	**7.2 (4.5)**	**8.0 (5.0)**
n	*258*	*248*	*245*	*239*	*232*	*223*	*194*	*128*	*40*	*5*
**Direct observation**	282.8 ± 96.9<br>(140 – 657)	365.4 ± 69.4<br>(195 – 666)	431.9 ± 52.1<br>(326 – 707)	484.7 ± 39.0<br>(386 – 653)	529.4 ± 33.2<br>(445 – 648)	568.9 ± 32.2<br>(501 – 659)	603.9 ± 34.4<br>(528 – 718)	644.0 ± 37.8<br>(562 – 742)	689.3 ± 43.2<br>(620 – 780)	727.4 ± 42.6<br>(675 – 787)
n	*250*	*241*	*238*	*232*	*226*	*218*	*190*	*125*	*39*	*5*
**Actical**	2.8 ± 21.8<br>(0 – 278)	21.2 ± 56.9<br>(0 – 650)	176.1 ± 128.9<br>(0 – 630)	419.7 ± 110.9<br>(0 – 648)	527.8 ± 78.5<br>(0 – 660)	564.9 ± 93.4<br>(0 – 675)	602.3 ± 94.7<br>(0 – 738)	642.0 ± 108.9<br>(0 – 818)	704.6 ± 43.9<br>(635 – 820)	740.0 ± 41.8<br>(685 – 795)
n	*254*	*244*	*241*	*235*	*229*	*220*	*191*	*126*	*40*	*5*
**ActiGraph GT9X (Waist)**	7.8 ± 28.8<br>(0 – 283)	64.5 ± 74.5<br>(0 – 505)	202.9 ± 103.0<br>(3 – 448)	418.9 ± 87.2<br>(30 – 558)	520.7 ± 49.1<br>(255 – 603)	567.2 ± 62.9<br>(3 – 675)	605.5 ± 51.7<br>(308 – 713)	633.5 ± 87.8<br>(0 – 815)	654.6 ± 104.9<br>(315 – 803)	607.0 ± 119.7<br>(418 – 728)
n	*254*	*244*	*241*	*235*	*229*	*220*	*192*	*126*	*40*	*5*
**ActiGraph GT9X (Wrist)**	52.1 ± 59.2<br>(0 – 315)	99.6 ± 67.7<br>(0 – 343)	118.6 ± 80.4<br>(0 – 413)	169.9 ± 95.2<br>(0 – 495)	235.8 ± 83.8<br>(5 – 563)	277.6 ± 75.6<br>(23 – 608)	310.0 ± 68.7<br>(68 – 738)	328.1 ± 59.2<br>(30 – 648)	357.6 ± 80.4<br>(245 – 800)	371.0 ± 22.7<br>(340 – 398)
n	*249*	*239*	*236*	*230*	*225*	*217*	*188*	*124*	*39*	*5*
**activPAL**	183.1 ± 121.9<br>(0 – 620)	352.6 ± 80.1<br>(20 – 660)	431.8 ± 50.8<br>(325 – 665)	484.6 ± 39.4<br>(380 – 650)	530.5 ± 33.2<br>(445 – 650)	569.4 ± 38.8<br>(270 – 665)	605.9 ± 34.4<br>(530 – 725)	646.0 ± 38.0<br>(560 – 775)	686.8 ± 38.2<br>(620 – 760)	717.0 ± 39.0<br>(675 – 780)
n	*174*	*164*	*161*	*155*	*149*	*140*	*117*	*60*	*15*	*1*
**Apple Watch Series 1***	108.6 ± 157.5<br>(0 – 641)	291.0 ± 132.8<br>(0 – 666)	423.5 ± 74.6<br>(115 – 701)	475.9 ± 58.6<br>(98 – 728)	524.1 ± 64.1<br>(399 – 981)	565.5 ± 42.6<br>(449 – 681)	587.6 ± 59.4<br>(217 – 740)	630.6 ± 67.0<br>(272 – 751)	697.5 ± 62.2<br>(624 – 850)	662.0
n	*258*	*248*	*245*	*238*	*232*	*220*	*194*	*127*	*40*	*5*
**Digiwalker SW-200**	23.0 ± 56.0<br>(0 – 435)	100.8 ± 116.3<br>(0 – 659)	241.5 ± 145.0<br>(0 – 703)	352.7 ± 139.4<br>(0 – 710)	448.1 ± 120.3<br>(23 – 642)	527.0 ± 90.9<br>(57 – 653)	582.4 ± 67.1<br>(302 – 700)	636.9 ± 45.9<br>(427 – 734)	688.3 ± 46.3<br>(570 – 780)	730.2 ± 47.7<br>(675 – 801)
n	*98*	*89*	*86*	*81*	*75*	*66*	*48*	*17*	*3*	
**Fitbit Ionic**†	48.3 ± 118.4<br>(0 – 526)	185.9 ± 160.9<br>(0 – 534)	404.1 ± 90.2<br>(22 – 581)	477.4 ± 49.6<br>(385 – 640)	517.6 ± 40.3<br>(384 – 634)	550.2 ± 42.8<br>(420 – 659)	576.0 ± 52.1<br>(442 – 705)	629.7 ± 53.1<br>(534 – 749)	631.0 ± 44.6<br>(580 – 663)	
n	*160*	*159*	*159*	*158*	*157*	*156*	*146*	*110*	*37*	*6*
**Fitbit One**‡	6.0 ± 32.1<br>(0 – 344)	211.1 ± 135.4<br>(0 – 666)	412.8 ± 48.7<br>(317 – 665)	477.1 ± 35.6<br>(382 – 651)	526.0 ± 32.3<br>(442 – 647)	566.5 ± 32.5<br>(498 – 652)	601.7 ± 32.7<br>(528 – 675)	642.0 ± 37.8<br>(562 – 740)	686.9 ± 43.9<br>(620 – 780)	605.2 ± 299.1<br>(0 – 786)

n	*98*	*89*	*86*	*81*	*75*	*66*	*48*	*17*	*3*	
**Fitbit Zip†**	3.8 ± 27.5<br>(0 – 267)	35.4 ± 75.6<br>(0 – 383)	385.7 ± 124.6<br>(0 – 563)	480.1 ± 63.4<br>(29 – 608)	533.1 ± 62.3<br>(467 – 979)	566.1 ± 31.9<br>(503 – 652)	603.6 ± 38.6<br>(526 – 712)	644.8 ± 41.3<br>(563 – 738)	681.0 ± 39.2<br>(640 – 718)	
n	*96*	*89*	*86*	*80*	*73*	*66*	*48*	*17*	*3*	
**Garmin vivoactive 3†**	47.7 ± 114.6<br>(0 – 553)	271.0 ± 156.7<br>(0 – 550)	430.6 ± 64.8<br>(236 – 636)	482.1 ± 37.2<br>(398 – 573)	515.2 ± 54.4<br>(154 – 615)	564.0 ± 33.0<br>(495 – 655)	597.2 ± 34.4<br>(527 – 707)	637.4 ± 38.7<br>(558 – 707)	671.3 ± 31.9<br>(637 – 700)	
n	*77*	*76*	*75*	*74*	*75*	*75*	*69*	*44*	*12*	*1*
**Garmin vivoactive HR****	35.1 ± 102.6<br>(0 – 502)	308.5 ± 146.2<br>(0 – 665)	402.8 ± 70.0<br>(47 – 627)	471.0 ± 38.3<br>(379 – 619)	518.5 ± 31.0<br>(437 – 595)	546.5 ± 45.6<br>(322 – 603)	556.8 ± 78<br>.0 (329 –<br>897)	527.2 ± 90.1<br>(326 – 727)	510.4 ± 105.9<br>(369 – 663)	492.0
n	*80*	*80*	*80*	*80*	*79*	*78*	*73*	*64*	*25*	*4*
**Garmin vivofit 2††**	15.5 ± 66.3<br>(0 – 525)	267.1 ± 130.8<br>(0 – 565)	401.7 ± 41.3<br>(320 – 515)	472.7 ± 33.7<br>(390 – 555)	524.9 ± 28.6<br>(460 – 580)	554.1 ± 36.9<br>(395 – 625)	547.9 ± 71.9<br>(295 – 675)	531.2 ± 105.1<br>(265 – 665)	572.4 ± 129.6<br>(325 – 790)	595.0 ± 102.7<br>(460 – 710)
n	*77*	*76*	*76*	*75*	*75*	*72*	*70*	*45*	*12*	*1*
**Garmin vivofit 3****	60.3 ± 123.0<br>(0 – 567)	337.1 ± 117.9<br>(21 – 665)	465.6 ± 95.2<br>(329 – 727)	477.9 ± 40.4<br>(413 – 651)	521.9 ± 34.7<br>(435 – 643)	566.8 ± 40.3<br>(497 – 757)	599.3 ± 42.7<br>(527 – 819)	637.9 ± 42.3<br>(563 – 725)	696.6 ± 52.3<br>(631 – 795)	781.0
n	*224*	*215*	*214*	*210*	*208*	*201*	*179*	*122*	*39*	*5*
**GENEActiv (Waist)**	236.3 ± 100.4<br>(30 – 625)	382.8 ± 75.5<br>(150 – 663)	430.6 ± 50.4<br>(325 – 660)	481.4 ± 37.9<br>(385 – 645)	529.3 ± 33.3<br>(445 – 650)	567.9 ± 35.5<br>(365 – 665)	605.2 ± 36.7<br>(468 – 725)	640.6 ± 72.2<br>(0 – 798)	694.9 ± 43.2<br>(623 – 788)	729.5 ± 42.6<br>(673 – 780)
n	*217*	*209*	*207*	*203*	*201*	*194*	*175*	*118*	*40*	*5*
**GENEActiv (Wrist)**	212.9 ± 106.6<br>(3 – 623)	392.4 ± 66.5<br>(208 – 670)	429.9 ± 45.4<br>(338 – 690)	471.2 ± 41.3<br>(333 – 625)	503.2 ± 51.0<br>(325 – 625)	525.4 ± 70.1<br>(295 – 665)	538.3 ± 90.7<br>(318 – 723)	552.1 ± 105.2<br>(303 – 740)	566.4 ± 126.4<br>(370 – 805)	578.5 ± 99.4<br>(425 – 685)
n	*258*	*248*	*245*	*239*	*232*	*222*	*194*	*126*	*40*	*5*
**NL–1000**	12.4 ± 46.4<br>(0 – 435)	95.8 ± 113.0<br>(0 – 679)	342.0 ± 120.6<br>(3 – 664)	458.8 ± 62.7<br>(74 – 653)	519.7 ± 59.2<br>(55 – 645)	565.2 ± 60.8<br>(90 – 967)	597.8 ± 60.4<br>(62 – 715)	643.7 ± 37.5<br>(562 – 741)	687.1 ± 41.1<br>(620 – 780)	723.6 ± 46.3<br>(670 – 793)
n	*98*	*89*	*85*	*81*	*75*	*66*	*48*	*17*	*3*	
**PiezoRx†**	85.7 ± 132.0<br>(0 – 553)	342.0 ± 126.9<br>(0 – 568)	448.2 ± 54.5<br>(217 – 578)	489.3 ± 37.6<br>(406 – 610)	530.8 ± 34.5<br>(465 – 639)	570.0 ± 33.4<br>(502 – 657)	594.2 ± 82.1<br>(105 – 717)	672.5 ± 130.1<br>(566 – 1159)	680.7 ± 36.7<br>(642 – 715)	
n	*97*	*87*	*85*	*80*	*74*	*65*	*47*	*17*	*3*	
**Polar M600†**	62.3 ± 123.6<br>(0 – 512)	174.0 ± 160.2<br>(4 – 564)	347.3 ± 134.7<br>(34 – 578)	463.4 ± 86<br>(20 – 617)	510.8 ± 65.6<br>(292 – 803)	552.4 ± 82.5<br>(24 – 708)	585.6 ± 63.8<br>(456 – 844)	626.2 ± 47.4<br>(548 – 731)	666.7 ± 36.1<br>(635 – 706)	
n	*80*	*79*	*79*	*77*	*77*	*78*	*73*	*47*	*12*	*1*
**Samsung Gear Fit2****	18.4 ± 75.9<br>(0 – 485)	106.1 ± 147.7<br>(0 – 631)	407.2 ± 119.4<br>(31 – 717)	467.1 ± 67.6<br>(1 – 638)	514.4 ± 36.7<br>(398 – 621)	557.7 ± 37.9<br>(467 – 664)	590.3 ± 33.8<br>(494 – 660)	627.0 ± 38.8<br>(557 – 711)	610.1 ± 69.0<br>(474 – 683)	620.0

n	*98*	*89*	*86*	*81*	*74*	*66*	*48*	*17*	*3*	
**Samsung Gear Fit2 Pro**†	33.9 ± 103.1 (0 – 589)	143.1 ± 157.7 (0 – 529)	386.4 ± 136.0 (0 – 679)	468.9 ± 38.9 (394 – 577)	508.8 ± 40.3 (439 – 613)	552.6 ± 41.9 (356 – 658)	584.9 ± 34.9 (485 – 685)	609.2 ± 27.5 (559 – 662)	637.0 ± 12.5 (625 – 650)	
n	*253*	*243*	*240*	*234*	*227*	*218*	*189*	*125*	*40*	*5*
**StepWatch**	271.7 ± 109.7 (45 – 625)	387.9 ± 65.0 (200 – 670)	433.8 ± 50.7 (355 – 690)	484.9 ± 38.3 (395 – 648)	530.3 ± 33.1 (450 – 650)	568.7 ± 37.2 (310 – 665)	605.2 ± 32.9 (530 – 700)	640.7 ± 35.6 (520 – 705)	636.0 ± 54.9 (530 – 705)	593.0 ± 86.9 (520 – 700)

Values represent the average number of steps ± standard deviation (minimum – maximum). *AppleWatch Series 1 was worn by Middle-Age (41–60 years) and Older Adults (61–85 years). †Fitbit Ionic, Fitbit Zip, Garmin vivoactive 3, PiezoRx, Polar M600, Samsung Gear Fit2 Pro were worn by Older Adults (61–85 years). ‡Fitbit One was worn by Young (21–41 years) and Middle-Age Adults (41–60 years). **Garmin vivoactive HR, Garmin vivofit 3, Samsung Gear Fit2 were worn by Middle-Age Adults (41–60 years). ††Garmin vivofit 2 was worn by Young Adults (21–41 years). See **Additional file 2** for a graphical classification of wearable technologies by age groups.

Additional File 3: Suppl Table 2 Sample sizes (*n*) and number of steps derived from direct observation and each wearable technology at each treadmill speed for Young Adults (21–40 years)

	**Treadmill speed, km/h (mph)**									
	**Slow**				**Normal**				**Fast**	
	**0.8 (0.5)**	**1.6 (1.0)**	**2.4 (1.5)**	**3.2 (2.0)**	**4.0 (2.5)**	**4.8 (3.0)**	**5.6 (3.5)**	**6.4 (4.0)**	**7.2 (4.5)**	**8.0 (5.0)**
n	*80*	*80*	*80*	*80*	*79*	*78*	*73*	*64*	*25*	*4*
**Direct observation**	225.3 ± 61.1<br>(140 – 505)	337.5 ± 44.9<br>(265 – 525)	418.0 ± 39.3<br>(360 – 550)	480.3 ± 32.1<br>(425 – 575)	529.2 ± 30.1<br>(465 – 605)	568.3 ± 30.2<br>(505 – 635)	606.0 ± 31.1<br>(540 – 675)	642.9 ± 32.1<br>(575 – 715)	685.0 ± 43.4<br>(620 – 780)	712.5 ± 30.7<br>(675 – 740)
n	*78*	*78*	*78*	*78*	*77*	*76*	*71*	*62*	*25*	*4*
**Actical**	1.6 ± 8.4<br>(0 – 70)	27.1 ± 52.9<br>(0 – 370)	224.3 ± 123.5<br>(0 – 520)	451.3 ± 86.8<br>(0 – 558)	531.4 ± 68.5<br>(0 – 615)	562.3 ± 97.9<br>(0 – 648)	607.7 ± 79.7<br>(0 – 685)	644.0 ± 89.6<br>(0 – 728)	699.0 ± 44.2<br>(635 – 820)	726.3 ± 32.8<br>(685 – 755)
n	*79*	*79*	*79*	*79*	*78*	*77*	*72*	*63*	*25*	*4*
**ActiGraph GT9X (Waist)**	3.8 ± 8.2<br>(0 – 45)	63.4 ± 64.7<br>(0 – 260)	207.7 ± 102.5<br>(23 – 425)	440.9 ± 77.6<br>(68 – 558)	522.6 ± 52.7<br>(263 – 588)	556.0 ± 83.8<br>(3 – 648)	601.0 ± 60.0<br>(308 – 685)	634.0 ± 71.3<br>(323 – 728)	656.6 ± 93.2<br>(358 – 803)	576.9 ± 114.3<br>(418 – 685)
n	*79*	*79*	*79*	*79*	*78*	*77*	*72*	*63*	*25*	*4*
**ActiGraph GT9X (Wrist)**	49.8 ± 41.7<br>(0 – 200)	109.4 ± 53.5<br>(25 – 310)	114.6 ± 62.5<br>(8 – 253)	166.7 ± 86.8<br>(3 – 378)	233.9 ± 75.8<br>(5 – 513)	269.7 ± 61.5<br>(78 – 575)	299.7 ± 54.5<br>(113 – 620)	322.0 ± 51.8<br>(168 – 648)	361.1 ± 95.4<br>(260 – 800)	364.4 ± 19.8<br>(340 – 385)
n	*76*	*76*	*76*	*76*	*75*	*74*	*69*	*61*	*24*	*4*
**activPAL**	151.1 ± 89.4<br>(0 – 455)	328.0 ± 56.2<br>(165 – 500)	418.1 ± 39.0<br>(355 – 550)	479.8 ± 32.1<br>(420 – 575)	529.9 ± 29.8<br>(465 – 605)	564.7 ± 45.9<br>(270 – 640)	606.8 ± 31.1<br>(540 – 675)	644.5 ± 32.4<br>(580 – 715)	679.4 ± 36.8<br>(620 – 745)	701.3 ± 19.3<br>(675 – 720)
n	*80*	*80*	*80*	*80*	*79*	*78*	*73*	*64*	*25*	*4*
**Digiwalker SW-200**	19.3 ± 53.0<br>(0 – 435)	107.4 ± 103.6<br>(0 – 510)	277.3 ± 127.8<br>(0 – 585)	401.2 ± 99.6<br>(120 – 710)	497.9 ± 55.4<br>(335 – 595)	559.2 ± 39.3<br>(415 – 625)	605.3 ± 36.6<br>(495 – 700)	641.7 ± 33.5<br>(535 – 705)	685.0 ± 47.3<br>(570 – 780)	712.5 ± 30.7<br>(675 – 740)
n	*80*	*80*	*80*	*80*	*79*	*78*	*73*	*64*	*25*	*5*
**Fitbit One**	2.8 ± 16.2<br>(0 – 135)	208.9 ± 119.0<br>(0 – 475)	413.4 ± 48.9<br>(320 – 655)	477.5 ± 31.2<br>(425 – 570)	527.2 ± 29.9<br>(460 – 605)	567.4 ± 31.4<br>(505 – 645)	605.1 ± 31.0<br>(535 – 675)	641.4 ± 33.3<br>(575 – 715)	683.8 ± 42.9<br>(620 – 780)	569.0 ± 319.3<br>(0 – 740)
n	*80*	*80*	*80*	*80*	*79*	*78*	*73*	*64*	*25*	*4*
**Garmin vivofit 2**	15.5 ± 66.3<br>(0 – 525)	267.1 ± 130.8<br>(0 – 565)	401.7 ± 41.3<br>(320 – 515)	472.7 ± 33.7<br>(390 – 555)	524.9 ± 28.6<br>(460 – 580)	554.1 ± 36.9<br>(395 – 625)	547.9 ± 71.9<br>(295 – 675)	531.2 ± 105.1<br>(265 – 665)	572.4 ± 129.6<br>(325 – 790)	595.0 ± 102.7<br>(460 – 710)
n	*78*	*78*	*78*	*78*	*77*	*76*	*71*	*62*	*25*	*4*
**GENEActiv (Waist)**	245.6 ± 65.0<br>(95 – 440)	385.6 ± 72.9<br>(215 – 583)	421.3 ± 44.0<br>(325 – 538)	477.0 ± 32.2<br>(410 – 558)	529.0 ± 29.5<br>(465 – 603)	565.1 ± 37.7<br>(365 – 638)	604.4 ± 35.2<br>(468 – 673)	641.7 ± 37.8<br>(493 – 715)	689.5 ± 43.1<br>(623 – 788)	716.9 ± 36.8<br>(673 – 755)

n	*76*	*76*	*76*	*76*	*75*	*74*	*70*	*61*	*25*	*4*
**GENEActiv (Wrist)**	216.3 ± 83.7 (40 – 468)	388.8 ± 56.7 (243 – 565)	424.0 ± 32.3 (360 – 510)	461.9 ± 35.6 (345 – 538)	488.7 ± 55.7 (325 – 563)	507.1 ± 67.2 (350 – 605)	506.0 ± 91.4 (320 – 635)	523.3 ± 99.6 (303 – 653)	563.2 ± 128.5 (370 – 805)	616.9 ± 57.9 (553 – 685)
n	*80*	*80*	*80*	*80*	*79*	*78*	*73*	*64*	*25*	*4*
**NL–1000**	9.6 ± 51.9 (0 – 435)	93.2 ± 116.2 (0 – 600)	359.9 ± 112.1 (115 – 625)	474.9 ± 45.6 (315 – 600)	521.6 ± 61.0 (55 – 610)	570.1 ± 30.2 (505 – 635)	606.8 ± 31.5 (535 – 675)	643.6 ± 32.4 (575 – 715)	683.0 ± 41.3 (620 – 780)	706.3 ± 29.3 (670 – 730)
n	*79*	*79*	*79*	*79*	*78*	*77*	*72*	*63*	*25*	*4*
**StepWatch**	218.7 ± 66.0 (90 – 505)	369.1 ± 50.0 (240 – 520)	418.2 ± 37.9 (365 – 540)	479.6 ± 31.9 (420 – 575)	529.9 ± 29.6 (470 – 605)	565.3 ± 41.9 (310 – 640)	606.5 ± 31.0 (540 – 675)	643.1 ± 30.0 (580 – 705)	639.4 ± 49.2 (535 – 705)	607.5 ± 93.1 (520 – 700)

Values represent the average number of steps ± standard deviation (minimum – maximum). See **Additional file 2** for a graphical classification of wearable technologies by age groups.

Additional File 3: Suppl Table 3 Sample sizes (*n*) and number of steps derived from direct observation and each wearable technology at each treadmill speed for Middle-Age Adults (41–60 years)

	**Treadmill speed, km/h (mph)**									
	**Slow**				**Normal**				**Fast**	
	**0.8 (0.5)**	**1.6 (1.0)**	**2.4 (1.5)**	**3.2 (2.0)**	**4.0 (2.5)**	**4.8 (3.0)**	**5.6 (3.5)**	**6.4 (4.0)**	**7.2 (4.5)**	**8.0 (5.0)**
n	*80*	*79*	*79*	*78*	*78*	*78*	*73*	*47*	*12*	*1*
**Direct observation**	262.5 ± 84.9 (154 – 604)	351.0 ± 71.1 (195 – 666)	423.0 ± 54.3 (326 – 707)	480.6 ± 41.2 (386 – 653)	527.8 ± 34.8 (445 – 648)	568.1 ± 34.2 (501 – 655)	600.4 ± 34.3 (528 – 676)	644.5 ± 43.8 (562 – 739)	699.3 ± 45.3 (639 – 759)	787.0
n	*78*	*77*	*77*	*76*	*76*	*76*	*71*	*46*	*11*	*1*
**Actical**	6.7 ± 38.0 (0 – 278)	28.0 ± 80.4 (0 – 650)	177.9 ± 130.4 (0 – 630)	426.8 ± 88.8 (218 – 648)	535.2 ± 33.1 (453 – 655)	578.0 ± 34.6 (510 – 670)	610.7 ± 33.8 (543 – 680)	644.6 ± 108.5 (0 – 818)	713.9 ± 43.3 (660 – 795)	795.0
n	*79*	*78*	*78*	*77*	*77*	*77*	*72*	*46*	*12*	*1*
**ActiGraph GT9X (Waist)**	5.7 ± 18.5 (0 – 138)	77.6 ± 86.6 (0 – 505)	233.1 ± 105.2 (3 – 425)	438.1 ± 74.8 (120 – 538)	523.0 ± 49.6 (255 – 595)	575.0 ± 38.5 (408 – 658)	608.3 ± 39.0 (473 – 685)	629.4 ± 113.1 (0 – 815)	694.2 ± 64.7 (520 – 758)	727.5
n	*79*	*78*	*78*	*77*	*77*	*77*	*72*	*46*	*12*	*1*
**ActiGraph GT9X (Wrist)**	39.5 ± 55.3 (0 – 300)	88.0 ± 75.7 (0 – 343)	103.3 ± 85.1 (0 – 353)	155.4 ± 98.5 (0 – 413)	230.8 ± 82.8 (5 – 400)	273.7 ± 73.6 (23 – 455)	309.8 ± 64.0 (68 – 498)	329.0 ± 63.9 (30 – 475)	346.7 ± 42.5 (245 – 395)	397.5
n	*78*	*77*	*77*	*76*	*76*	*76*	*71*	*46*	*12*	*1*
**activPAL**	163.0 ± 121.7 (0 – 565)	337.4 ± 85.8 (20 – 660)	422.5 ± 52 (325 – 665)	480.9 ± 41.8 (380 – 650)	528.6 ± 35.1 (445 – 645)	569.7 ± 35.3 (505 – 655)	601.3 ± 34.3 (530 – 670)	645.1 ± 43.9 (560 – 775)	697.5 ± 37.1 (645 – 750)	780.0
n	*76*	*75*	*75*	*74*	*74*	*74*	*69*	*43*	*12*	*1*
**Apple Watch Serie 1**	54.3 ± 129.8 (0 – 580)	268.7 ± 144.1 (0 – 666)	420.8 ± 71.0 (115 – 701)	473.7 ± 44.0 (378 – 648)	519.4 ± 39.8 (399 – 640)	562.5 ± 42.1 (449 – 681)	598.3 ± 39.5 (528 – 740)	631.6 ± 47.5 (495 – 739)	704.3 ± 66.9 (624 – 850)	662.0
n	*80*	*79*	*79*	*77*	*78*	*78*	*73*	*46*	*12*	*1*
**Digiwalker SW-200**	30.0 ± 67.9 (0 – 354)	129.4 ± 140.1 (0 – 659)	279.1 ± 140.3 (2 – 703)	404.1 ± 103.0 (82 – 644)	498.9 ± 66.3 (256 – 642)	558.4 ± 41.8 (422 – 653)	598.9 ± 38.8 (500 – 696)	644.0 ± 43.3 (564 – 734)	701.9 ± 47.4 (640 – 771)	801.0
n	*80*	*79*	*79*	*78*	*78*	*78*	*73*	*46*	*12*	*1*
**Fitbit One**	9.2 ± 42.3 (0 – 344)	213.4 ± 151.0 (0 – 666)	412.2 ± 48.9 (317 – 665)	476.7 ± 39.8 (382 – 651)	524.8 ± 34.7 (442 – 647)	565.6 ± 33.9 (498 – 652)	598.2 ± 34.1 (528 – 675)	642.8 ± 43.7 (562 – 740)	693.3 ± 47.3 (641 – 764)	786.0
n	*77*	*76*	*75*	*74*	*75*	*75*	*69*	*44*	*12*	*1*
**Garmin vivoactive HR**	35.1 ± 102.6 (0 – 502)	308.5 ± 146.2 (0 – 665)	402.8 ± 70.0 (47 – 627)	471.0 ± 38.3 (379 – 619)	518.5 ± 31.0 (437 – 595)	546.5 ± 45.6 (322 – 603)	556.8 ± 78.0 (329 – 897)	527.2 ± 90.1 (326 – 727)	510.4 ± 105.9 (369 – 663)	492.0

n	*77*	*76*	*76*	*75*	*75*	*72*	*70*	*45*	*12*	*1*
**Garmin vivofit 3**	60.3 ± 123.0 (0 – 567)	337.1 ± 117.9 (21 – 665)	465.6 ± 95.2 (329 – 727)	477.9 ± 40.4 (413 – 651)	521.9 ± 34.7 (435 – 643)	566.8 ± 40.3 (497 – 757)	599.3 ± 42.7 (527 – 819)	637.9 ± 42.3 (563 – 725)	696.6 ± 52.3 (631 – 795)	781.0
n	*72*	*71*	*71*	*70*	*70*	*70*	*66*	*44*	*11*	*1*
**GENEActiv (Waist)**	234.7 ± 111.9 (40 – 615)	369.2 ± 86.9 (150 – 663)	423.1 ± 53.0 (343 – 660)	479.4 ± 41.7 (385 – 645)	526.8 ± 35.9 (445 – 645)	568.1 ± 35.4 (503 – 658)	600.8 ± 35.0 (530 – 673)	634.1 ± 109.0 (0 – 798)	704.8 ± 44.4 (650 – 780)	780.0
n	*68*	*67*	*67*	*66*	*66*	*66*	*63*	*41*	*12*	*1*
**GENEActiv (Wrist)**	208.7 ± 123.6 (3 – 603)	399.2 ± 79.6 (208 – 670)	427.8 ± 56.7 (338 – 690)	471.5 ± 44.6 (333 – 625)	504.1 ± 48.9 (330 – 620)	522.4 ± 72.9 (295 – 628)	535.4 ± 85.5 (318 – 655)	564.5 ± 104.3 (318 – 720)	540.0 ± 120.5 (370 – 700)	425.0
n	*80*	*79*	*79*	*78*	*78*	*78*	*73*	*45*	*12*	*1*
**NL–1000**	16.8 ± 56.4 (0 – 351)	93.9 ± 122.1 (0 – 679)	333.9 ± 124.7 (35 – 664)	451.2 ± 57.2 (312 – 653)	519.4 ± 58.4 (116 – 645)	572.1 ± 56.6 (499 – 967)	598.3 ± 37.0 (511 – 676)	644.5 ± 43.8 (565 – 741)	698.3 ± 43.0 (643 – 765)	793.0
n	*80*	*79*	*79*	*77*	*77*	*78*	*73*	*47*	*12*	*1*
**Samsung Gear Fit2**	18.4 ± 75.9 (0 – 485)	106.1 ± 147.7 (0 – 631)	407.2 ± 119.4 (31 – 717)	467.1 ± 67.6 (1 – 638)	514.4 ± 36.7 (398 – 621)	557.7 ± 37.9 (467 – 664)	590.3 ± 33.8 (494 – 660)	627 ± 38.8 (557 – 711)	610.1 ± 69.0 (474 – 683)	620.0
n	*79*	*78*	*78*	*77*	*77*	*77*	*72*	*46*	*12*	*1*
**StepWatch**	242.8 ± 107.2 (45 – 620)	377.7 ± 66.8 (200 – 670)	425.7 ± 51.7 (365 – 690)	481.6 ± 40.1 (395 – 645)	528.6 ± 34.7 (450 – 645)	569.9 ± 35.0 (500 – 660)	602.2 ± 34.9 (530 – 675)	639.2 ± 40.2 (530 – 705)	629.6 ± 63.6 (530 – 705)	535.0

Values represent the average number of steps ± standard deviation (minimum – maximum). See **Additional file 2** for a graphical classification of wearable technologies by age groups.

Additional File 3: Suppl Table 4 Sample sizes (*n*) and number of steps derived from direct observation and each wearable technology at each treadmill speed for Older Adults (61–85 years)

	**Treadmill speed, km/h (mph)**								
	**Slow**				**Normal**				**Fast**
	**0.8 (0.5)**	**1.6 (1.0)**	**2.4 (1.5)**	**3.2 (2.0)**	**4.0 (2.5)**	**4.8 (3.0)**	**5.6 (3.5)**	**6.4 (4.0)**	**7.2 (4.5)**
n	*98*	*89*	*86*	*81*	*75*	*67*	*48*	*17*	*3*
**Direct observation**	346.4 ± 93.9 (190 – 657)	403.1 ± 70.0 (253 – 601)	453.0 ± 54.2 (332 – 626)	493.1 ± 42.1 (407 – 649)	531.3 ± 35.1 (467 – 640)	570.7 ± 32.7 (503 – 659)	606.2 ± 39.4 (531 – 718)	647.1 ± 41.7 (563 – 742)	684.7 ± 39.6 (644 – 723)
n	*94*	*86*	*83*	*78*	*73*	*66*	*48*	*17*	*3*
**Actical**	0.4 ± 2.2 (0 – 15)	9.8 ± 24.6 (0 – 155)	129.2 ± 116.3 (0 – 498)	381.3 ± 138.4 (0 – 620)	516.4 ± 113.9 (0 – 660)	553.0 ± 127.8 (0 – 675)	581.9 ± 156.0 (0 – 738)	627.9 ± 167.4 (0 – 765)	716.7 ± 51.4 (668 – 770)
n	*96*	*87*	*84*	*79*	*74*	*66*	*47*	*17*	*3*
**ActiGraph GT9X (Waist)**	12.8 ± 42.8 (0 – 283)	53.8 ± 70.0 (0 – 375)	170.4 ± 92.7 (8 – 448)	378.2 ± 94.0 (30 – 525)	516.3 ± 44.9 (338 – 603)	571.1 ± 56.2 (248 – 675)	608.1 ± 55.6 (368 – 713)	642.4 ± 66.8 (448 – 750)	479.2 ± 177.5 (315 – 668)
n	*96*	*87*	*84*	*79*	*74*	*66*	*48*	*17*	*3*
**ActiGraph GT9X (Wrist)**	64.2 ± 71.3 (0 – 315)	100.9 ± 70.7 (3 – 270)	136.7 ± 88.1 (5 – 413)	187.4 ± 98.3 (5 – 495)	243.0 ± 93.1 (28 – 563)	291.3 ± 90.8 (58 – 608)	325.6 ± 89.9 (165 – 738)	348.5 ± 70.2 (188 – 518)	371.7 ± 75.6 (315 – 458)
n	*95*	*86*	*83*	*78*	*74*	*67*	*48*	*17*	*3*
**activPAL**	225.2 ± 133.1 (0 – 620)	388.0 ± 81.2 (220 – 575)	453.0 ± 52.8 (335 – 605)	492.9 ± 42.4 (395 – 650)	533.0 ± 34.6 (465 – 650)	574.3 ± 33.5 (510 – 665)	611.6 ± 38.7 (535 – 725)	654.1 ± 41.3 (575 – 745)	703.3 ± 53.0 (655 – 760)
n	*98*	*89*	*86*	*81*	*75*	*66*	*48*	*17*	*3*
**Apple Watch Serie 1**	150.7 ± 164.7 (0 – 641)	309.8 ± 120.2 (0 – 586)	425.9 ± 77.9 (118 – 564)	478.0 ± 69.6 (98 – 728)	528.6 ± 81.4 (418 – 981)	568.9 ± 43.2 (490 – 677)	572.2 ± 77.7 (217 – 723)	627.9 ± 103.1 (272 – 751)	670.0 ± 31.8 (635 – 697)
n	*98*	*89*	*86*	*81*	*75*	*64*	*48*	*17*	*3*
**Digiwalker SW-200**	20.5 ± 47.1 (0 – 238)	69.3 ± 95.6 (0 – 432)	173.6 ± 141.0 (0 – 518)	255.9 ± 152.7 (0 – 574)	342.7 ± 144.0 (23 – 576)	449.6 ± 126.9 (57 – 624)	522.6 ± 96.0 (302 – 662)	599.5 ± 72.2 (427 – 723)	660.7 ± 13.7 (646 – 673)
n	*98*	*89*	*86*	*81*	*75*	*66*	*48*	*17*	*3*
**Fitbit Ionic**	48.3 ± 118.4 (0 – 526)	185.9 ± 160.9 (0 – 534)	404.1 ± 90.2 (22 – 581)	477.4 ± 49.6 (385 – 640)	517.6 ± 40.3 (384 – 634)	550.2 ± 42.8 (420 – 659)	576.0 ± 52.1 (442 – 705)	629.7 ± 53.1 (534 – 749)	631.0 ± 44.6 (580 – 663)
n	*98*	*89*	*86*	*81*	*75*	*66*	*48*	*17*	*3*
**Fitbit Zip**	3.8 ± 27.5 (0 – 267)	35.4 ± 75.6 (0 – 383)	385.7 ± 124.6 (0 – 563)	480.1 ± 63.4 (29 – 608)	533.1 ± 62.3 (467 – 979)	566.1 ± 31.9 (503 – 652)	603.6 ± 38.6 (526 – 712)	644.8 ± 41.3 (563 – 738)	681.0 ± 39.2 (640 – 718)

n	*96*	*89*	*86*	*80*	*73*	*66*	*48*	*17*	*3*
**Garmin vivoactive 3**	47.7 ± 114.6 (0 – 553)	271.0 ± 156.7 (0 – 550)	430.6 ± 64.8 (236 – 636)	482.1 ± 37.2 (398 – 573)	515.2 ± 54.4 (154 – 615)	564.0 ± 33.0 (495 – 655)	597.2 ± 34.4 (527 – 707)	637.4 ± 38.7 (558 – 707)	671.3 ± 31.9 (637 – 700)
n	*74*	*66*	*65*	*62*	*61*	*55*	*42*	*16*	*3*
**GENEActiv (Waist)**	228.0 ± 118.3 (30 – 625)	394.2 ± 63.1 (243 – 575)	450.1 ± 50.2 (333 – 580)	489.3 ± 39.4 (400 – 610)	532.6 ± 35.1 (468 – 650)	571.5 ± 32.6 (510 – 665)	613.3 ± 40.9 (533 – 725)	654.4 ± 43.5 (570 – 750)	703.3 ± 47.6 (658 – 753)
n	*73*	*66*	*64*	*61*	*60*	*54*	*42*	*16*	*3*
**GENEActiv (Wrist)**	213.3 ± 112.1 (8 – 623)	389.6 ± 62.7 (223 – 563)	439.1 ± 44.8 (348 – 573)	482.5 ± 41.8 (355 – 598)	520.2 ± 41.5 (378 – 625)	554.2 ± 61.9 (308 – 665)	596.4 ± 67.8 (350 – 723)	629.8 ± 86.2 (345 – 740)	699.2 ± 51.4 (650 – 753)
n	*98*	*89*	*86*	*81*	*75*	*66*	*48*	*17*	*3*
**NL–1000**	11.2 ± 30.1 (0 – 214)	99.9 ± 102.4 (0 – 440)	332.7 ± 123.9 (3 – 620)	450.2 ± 78.1 (74 – 596)	518.0 ± 58.9 (112 – 632)	551.3 ± 86.2 (90 – 652)	583.1 ± 104.9 (62 – 715)	641.6 ± 39.7 (562 – 732)	675.7 ± 33.7 (640 – 707)
n	*98*	*89*	*85*	*81*	*75*	*66*	*48*	*17*	*3*
**PiezoRx**	85.7 ± 132.0 (0 – 553)	342.0 ± 126.9 (0 – 568)	448.2 ± 54.5 (217 – 578)	489.3 ± 37.6 (406 – 610)	530.8 ± 34.5 (465 – 639)	570.0 ± 33.4 (502 – 657)	594.2 ± 82.1 (105 – 717)	672.5 ± 130.1 (566 – 1159)	680.7 ± 36.7 (642 – 715)
n	*97*	*87*	*85*	*80*	*74*	*65*	*47*	*17*	*3*
**Polar M600**	62.3 ± 123.6 (0 – 512)	174.0 ± 160.2 (4 – 564)	347.3 ± 134.7 (34 – 578)	463.4 ± 86.0 (20 – 617)	510.8 ± 65.6 (292 – 803)	552.4 ± 82.5 (24 – 708)	585.6 ± 63.8 (456 – 844)	626.2 ± 47.4 (548 – 731)	666.7 ± 36.1 (635 – 706)
n	*98*	*89*	*86*	*81*	*74*	*66*	*48*	*17*	*3*
**Samsung Gear Fit2 Pro**	33.9 ± 103.1 (0 – 589)	143.1 ± 157.7 (0 – 529)	386.4 ± 136.0 (0 – 679)	468.9 ± 38.9 (394 – 577)	508.8 ± 40.3 (439 – 613)	552.6 ± 41.9 (356 – 658)	584.9 ± 34.9 (485 – 685)	609.2 ± 27.5 (559 – 662)	637.0 ± 12.5 (625 – 650)
n	*95*	*86*	*83*	*78*	*72*	*64*	*45*	*16*	*3*
**StepWatch**	339.7 ± 106.6 (70 – 625)	414.4 ± 67.6 (245 – 592)	456.1 ± 53.1 (355 – 622)	493.4 ± 41.2 (410 – 648)	532.4 ± 35.1 (468 – 650)	571.4 ± 34.0 (505 – 665)	608.0 ± 32.7 (535 – 700)	635.3 ± 42.9 (520 – 685)	633.3 ± 84.6 (540 – 705)

Values represent the average number of steps ± standard deviation (minimum – maximum). See **Additional file 2** for a graphical classification of wearable technologies by age groups.
