# Supplementary material for: A catalog of validity indices for step counting wearable technologies during treadmill walking: the CADENCE-adults study
Source: Int J Behav Nutr Phys Act. 2022 Sep 8;19:117. doi: 10.1186/s12966-022-01350-9 (PMC9461139; doi:10.1186/s12966-022-01350-9)
Supplement: Supplementary file 6 — Additional file 6. Figures for descriptive Mean Percentage Error (MPE) representing bias of each wearable technology across walking speeds, and also presented by wear location and by age groups. [file 12966_2022_1350_MOESM6_ESM.pdf]

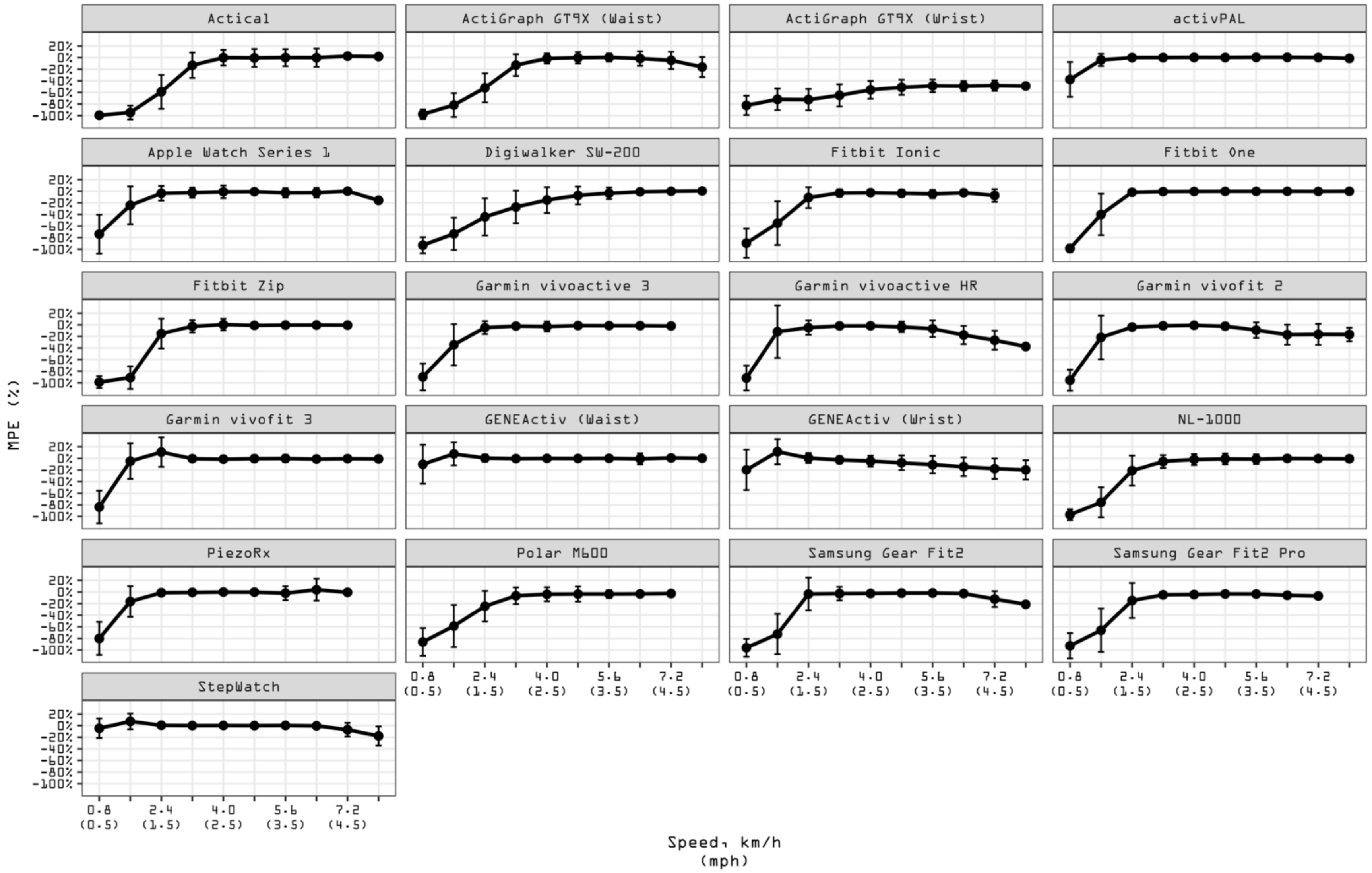

Additional file 6: Suppl Fig 1 Mean percentage error (MPE) of each wearable technology across walking speeds. Participants walked on a treadmill for 5-min bouts beginning at 0.8 km/h (0.5 mph) and increasing in 0.8 km/h (0.5 mph). MPE was computed for each

person bout subtracting the directly observed steps (criterion measurement) from the wearable technology-derived steps and dividing it by the directly observed steps. The result is a scaled index that explains the difference, regardless of the total number of steps. Black dots represent the averaged MPE across all sample for a given speed. Bars represent standard deviation of MPE. The standard deviation bars are not drawn when they are shorter than the height of the symbol. MPE values closer to 0% indicate improved bias of the wearable technology of interest. See **Additional file 2** for a graphical classification of wearable technologies by age groups.

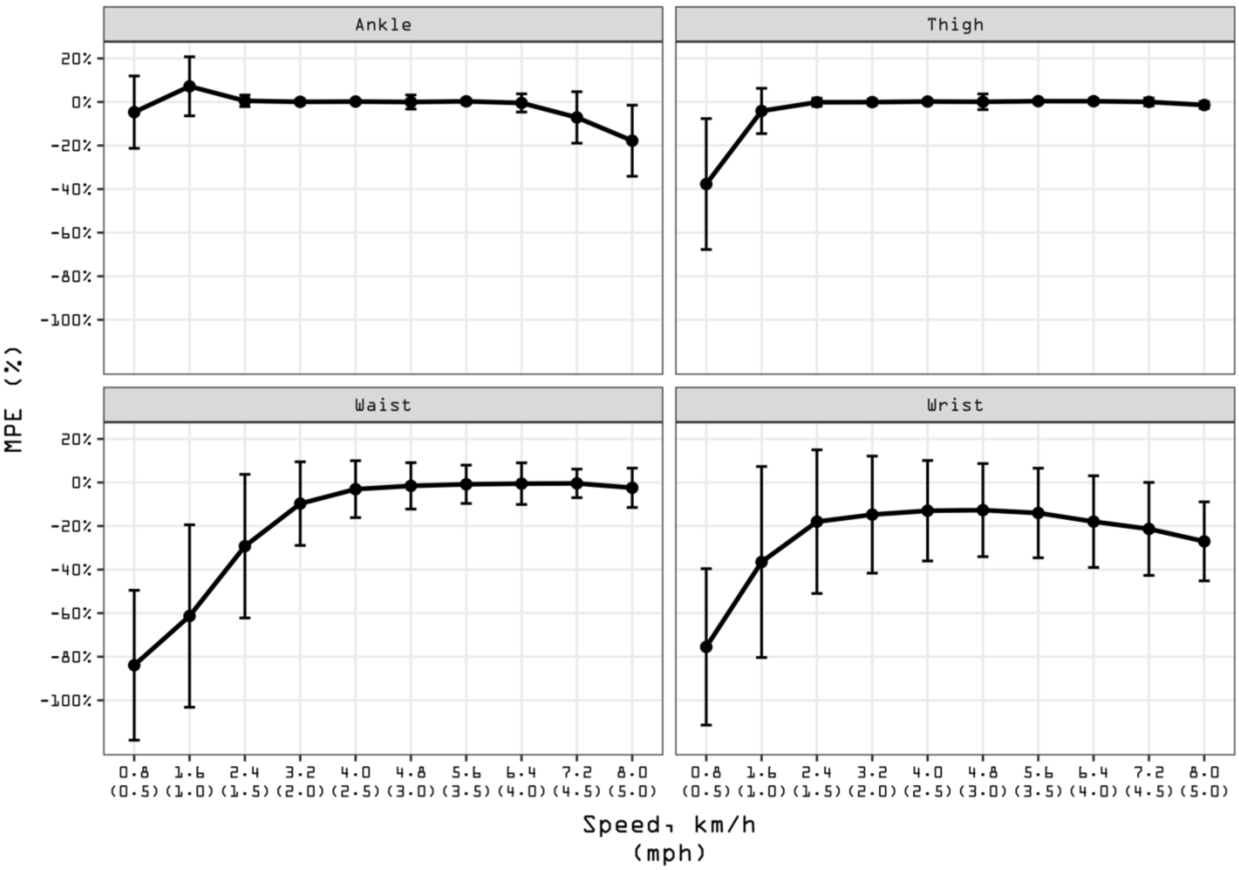

Additional file 6: Suppl Fig 2 Mean percentage error (MPE) across walking speeds presented by wear location. Participants walked on a treadmill for 5-min bouts beginning at 0.8 km/h (0.5 mph) and increasing in 0.8 km/h (0.5 mph). MPE was computed for each person bout subtracting the directly observed steps (criterion measurement) from the wearable technology-derived steps and dividing it by the directly observed steps. The result is a scaled index that explains the difference, regardless of the total number of steps. Black dots represent the averaged MPE across specific wear location for a given speed. Bars represent standard deviation of MPE. The standard deviation bars are not drawn when they are shorter than the height of the symbol. MPE values closer to 0% indicate improved bias of wear location of interest. Ankle-worn wearable: StepWatch. Thigh-worn wearable: activPAL. Waist-worn wearables: Actical, ActiGraph GT9X, Digi-Walker SW-200, Fitbit One, Fitbit Zip, GENEActiv, NL-1000, PiezoRx. Wrist-worn wearables: ActiGraph GT9X,

Apple Watch Serie 1, Fitbit Ionic, Garmin vivoactive 3, Garmin vivoactive HR, Garmin vivofit 2, Garmin vivofit 3, GENEActiv, Polar M600, Samsung Gear Fit2, Samsung Gear Fit2 Pro. See **Additional file 2** for a graphical classification of wearable technologies by age groups and **Additional File 8: Suppl Table 1** for a tabular description of validity indices by wear locations.

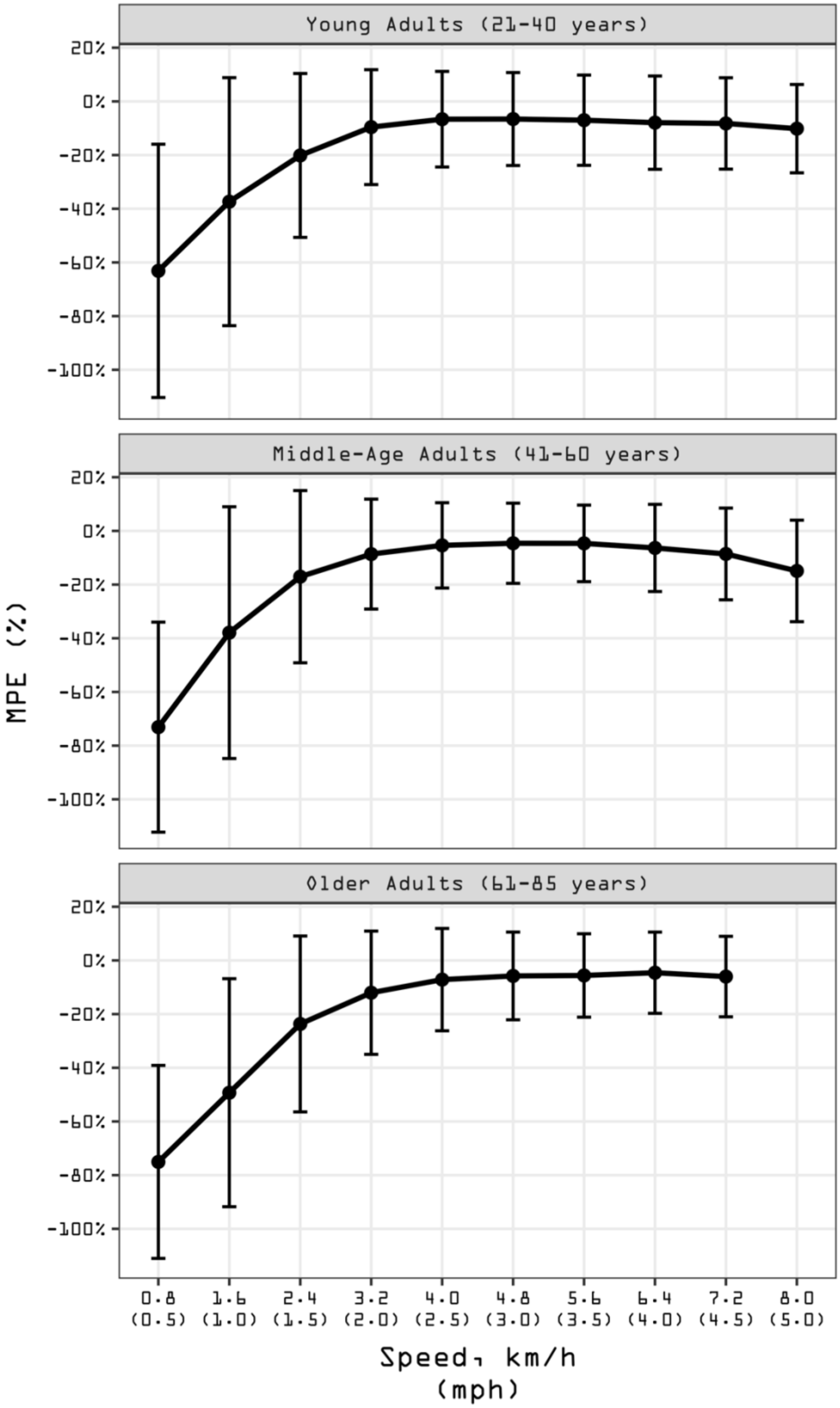

Additional file 6: Suppl Fig 3 Mean percentage error (MPE) across walking speeds presented by age group. Participants walked on a treadmill for 5-min bouts beginning at 0.8 km/h (0.5 mph) and

increasing in 0.8 km/h (0.5 mph). MPE was computed for each person bout subtracting the directly observed steps (criterion measurement) from the wearable technology-derived steps and dividing it by the directly observed steps. The result is a scaled index that explains the difference, regardless of the total number of steps. Black dots represent the averaged MPE across specific wear location or age group for a given speed. Bars represent standard deviation of MPE. MPE values closer to 0% indicate improved bias of the age group of interest. All age groups (21-85 years) wore the Actical, ActiGraph GT9X (Waist), ActiGraph GT9X (Wrist), activPAL, Digi-Walker SW-200, GENEActiv (Waist), GENEActiv (Wrist), NL-1000, and the StepWatch. Young Adults (21-40 years) also wore the Fitbit One and Garmin vivofit 2. Middle-Age Adults (41-60 years) also wore the Apple Watch Serie 1, Fitbit One, Garmin vivoactive HR, Garmin vivofit 3, and the Samsung Gear Fit2. Older Adults (61-85 years) also wore the AppleWatch Series 1, Fitbit Ionic, Fitbit Zip, Garmin vivoactive 3, PiezoRx, Polar M600, and the Samsung Gear Fit2 Pro. See **Additional file 2** for a graphical classification of wearable technologies by age groups. See **Additional File 8: Suppl Table 2** for a tabular description of validity indices by age groups.
