# Supplementary material for: A catalog of validity indices for step counting wearable technologies during treadmill walking: the CADENCE-adults study
Source: Int J Behav Nutr Phys Act. 2022 Sep 8;19:117. doi: 10.1186/s12966-022-01350-9 (PMC9461139; doi:10.1186/s12966-022-01350-9)
Supplement: Supplementary file 7 — Additional file 7. Graphical representation of correlation coefficients (r) of the relationship between directly observed steps and steps derived from wearable technologies. [file 12966_2022_1350_MOESM7_ESM.pdf]

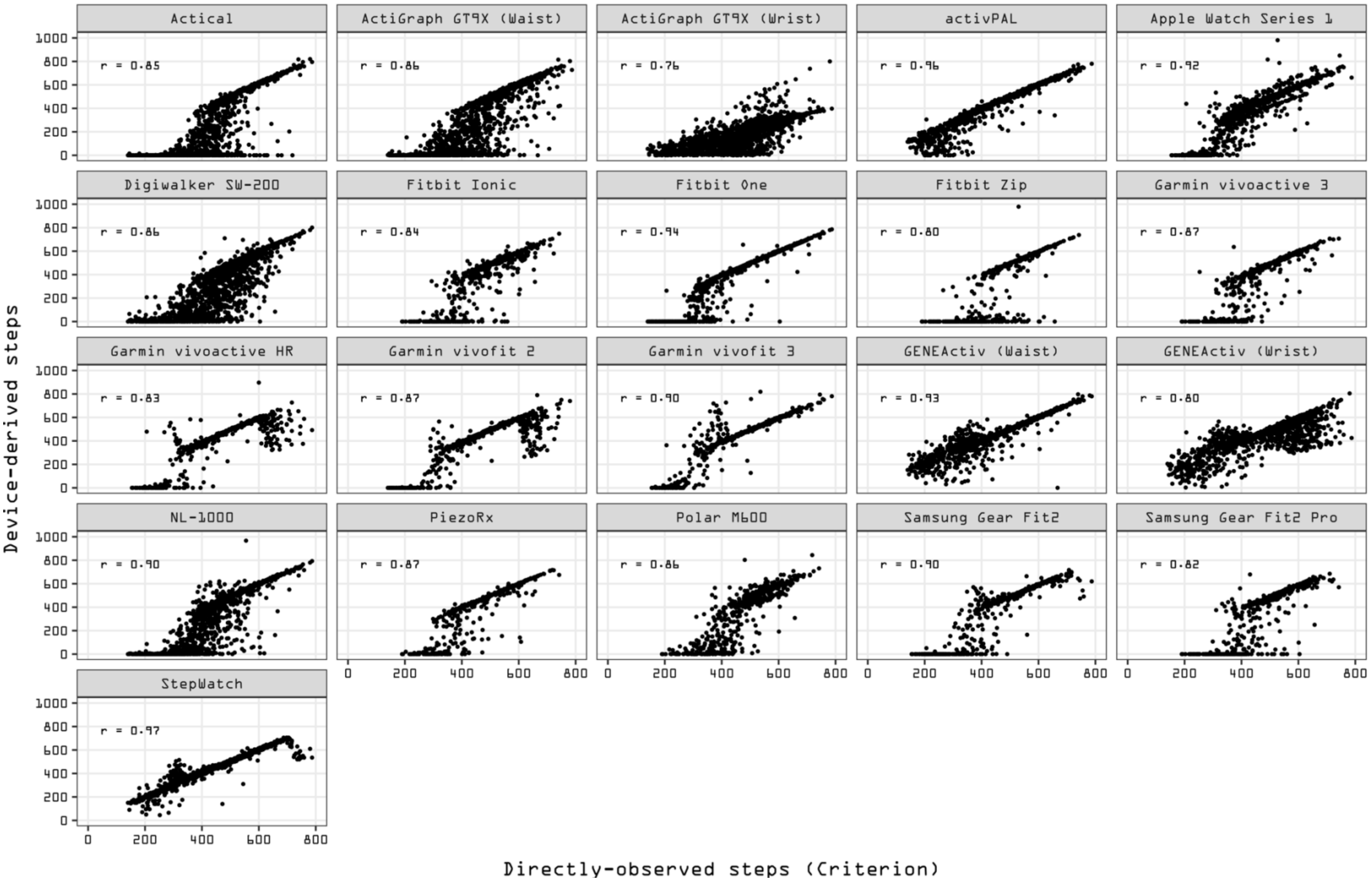

Additional file 7: Suppl Fig 1 Representation of the correlation between directly observed steps and steps derived from wearable technologies across all walking bouts. The X-Y scatterplot is representative of how tightly the wearable technology step counts hold to

a linear relationship to directly observed steps across all walking bouts. X axis represents directly observed steps, and Y axis represents steps derived from wearable technology. Correlation coefficients (*r*) closer to 1.0 indicate tighter relationship (more precise) to directly-observed steps.
