# Supplementary material for: A catalog of validity indices for step counting wearable technologies during treadmill walking: the CADENCE-adults study
Source: Int J Behav Nutr Phys Act. 2022 Sep 8;19:117. doi: 10.1186/s12966-022-01350-9 (PMC9461139; doi:10.1186/s12966-022-01350-9)
Supplement: Supplementary file 8 — Additional file 8. Tabular description of validity indices by wear locations and by age groups. [file 12966_2022_1350_MOESM8_ESM.pdf]

Additional File 8: Suppl Table 1 Validity indices (accuracy, bias, and precision) averaged across wear location for each speed bout

	**Treadmill speed, km/h (mph)**									
	**Slow**				**Normal**				**Fast**	
	**0.8 (0.5)**	**1.6 (1.0)**	**2.4 (1.5)**	**3.2 (2.0)**	**4.0 (2.5)**	**4.8 (3.0)**	**5.6 (3.5)**	**6.4 (4.0)**	**7.2 (4.5)**	**8.0 (5.0)**
Ankle										
**MAPE ± SD**	9.3 ± 14.5	8.4 ± 12.8	1.6 ± 2.2	0.9 ± 0.9	0.8 ± 0.7	1.0 ± 3.1	0.8 ± 1.1	1.5 ± 3.9	8.1 ± 11.1	17.8 ± 16.3
**MPE ± SD**	-4.7 ± 16.6	7.2 ± 13.6	0.5 ± 2.7	0.1 ± 1.3	0.2 ± 1.0	0.0 ± 3.2	0.3 ± 1.3	-0.4 ± 4.2	-7.1 ± 11.8	-17.8 ± 16.3
Thigh										
**MAPE ± SD**	37.9 ± 29.8	5.8 ± 9.6	1.3 ± 1.3	1.0 ± 1.0	0.8 ± 0.8	1.0 ± 3.4	0.8 ± 0.8	0.9 ± 1.0	1.2 ± 1.3	1.4 ± 1.6
**MPE ± SD**	-37.7 ± 30.0	-4.1 ± 10.4	-0.1 ± 1.8	-0.1 ± 1.4	0.2 ± 1.1	0.1 ± 3.6	0.4 ± 1.0	0.3 ± 1.3	0.0 ± 1.8	-1.4 ± 1.6
Waist										
**MAPE ± SD**	86.3 ± 27.8	64.7 ± 36.4	30.8 ± 31.5	10.5 ± 18.8	4.5 ± 12.7	3.1 ± 10.3	2.4 ± 8.5	2.3 ± 9.3	2.3 ± 6.2	3.5 ± 8.7
**MPE ± SD**	-83.9 ± 34.4	-61.3 ± 41.9	-29.2 ± 33.0	-9.7 ± 19.2	-3.1 ± 13.1	-1.6 ± 10.6	-0.8 ± 8.8	-0.5 ± 9.5	-0.4 ± 6.6	-2.4 ± 9.0
Wrist										
**MAPE ± SD**	77.7 ± 30.8	45.1 ± 35.0	22.6 ± 30.0	15.7 ± 26.4	13.8 ± 22.6	13.4 ± 20.9	14.6 ± 20.1	18.2 ± 20.8	22.0 ± 20.6	27.0 ± 18.1
**MPE ± SD**	-75.5 ± 35.9	-36.5 ± 43.8	-18.0 ± 33.0	-14.8 ± 26.9	-13.0 ± 23.1	-12.7 ± 21.4	-14.0 ± 20.6	-18.0 ± 21.0	-21.3 ± 21.3	-27.0 ± 18.1

All mean absolute percentage error (MAPE) and mean percentage error (MPE) values are presented as mean percentage ± standard deviation (SD). MAPE and SD values closer to 0% indicate higher accuracy and precision, respectively. MPE values closer to 0% indicate lower bias. Ankle-worn wearable: StepWatch. Thigh-worn wearable: activPAL. Waist-worn wearables: Actical, ActiGraph GT9X, Digi-Walker SW-200, Fitbit One, Fitbit Zip, GENEActiv, NL-1000, PiezoRx. Wrist-worn wearables: ActiGraph GT9X, Apple Watch Serie 1, Fitbit Ionic, Garmin vivoactive 3, Garmin vivoactive HR, Garmin vivofit 2, Garmin vivofit 3, GENEActiv, Polar M600, Samsung Gear Fit2, Samsung Gear Fit2 Pro. **See Additional file 2** for a graphical classification of wearable technologies by age groups.

Additional File 8: Suppl Table 2 Validity indices (accuracy, bias, and precision) averaged across all wearable technologies and presented by age group

	**Treadmill speed, km/h (mph)**									
	**Slow**				**Normal**				**Fast**	
	**0.8 (0.5)**	**1.6 (1.0)**	**2.4 (1.5)**	**3.2 (2.0)**	**4.0 (2.5)**	**4.8 (3.0)**	**5.6 (3.5)**	**6.4 (4.0)**	**7.2 (4.5)**	**8.0 (5.0)**
Young Adults (21–40 years)										
**MAPE ± SD**	68.8 ± 38.4	47.2 ± 36.1	22.7 ± 28.6	10.4 ± 21.0	7.5 ± 17.4	7.4 ± 17.0	7.9 ± 16.4	8.7 ± 17.0	9.4 ± 16.4	10.7 ± 16.1
**MPE ± SD**	-63.1 ± 47.2	-37.4 ± 46.2	-20.1 ± 30.5	-9.6 ± 21.4	-6.6 ± 17.8	-6.6 ± 17.3	-7.0 ± 16.8	-7.9 ± 17.4	-8.2 ± 17.0	-10.2 ± 16.5
Middle-Age Adults (41–60 years)										
**MAPE ± SD**	75.0 ± 35.5	46.4 ± 38.5	21.6 ± 29.2	9.5 ± 20.1	6.2 ± 15.6	5.8 ± 14.5	5.8 ± 13.8	7.5 ± 15.8	10.0 ± 16.3	15.4 ± 18.5
**MPE ± SD**	-73.1 ± 39.1	-37.9 ± 46.9	-17.1 ± 32.1	-8.6 ± 20.5	-5.4 ± 15.9	-4.6 ± 14.9	-4.6 ± 14.3	-6.3 ± 16.2	-8.6 ± 17.1	-14.9 ± 18.9
Older Adults (61-85 years)										
**MAPE ± SD**	75.9 ± 34.3	51.4 ± 39.9	25.2 ± 31.6	12.9 ± 22.5	8.7 ± 18.4	7.1 ± 15.8	7.0 ± 15.0	6.4 ± 14.4	7.9 ± 14.1	NA
**MPE ± SD**	-75.1 ± 36.0	-49.3 ± 42.5	-23.7 ± 32.8	-12.0 ± 23.0	-7.2 ± 19.1	-5.8 ± 16.3	-5.6 ± 15.5	-4.6 ± 15.1	-6.0 ± 15.0	NA

All mean absolute percentage error (MAPE) and mean percentage error (MPE) values are presented as mean percentage ± standard deviation (SD). MAPE and SD values closer to 0% indicate higher accuracy and precision, respectively. MPE values closer to 0% indicate lower bias. All age groups (21-85 years) wore the Actical, ActiGraph GT9X (Waist), ActiGraph GT9X (Wrist), activPAL, Digi-Walker SW-200, GENEActiv (Waist), GENEActiv (Wrist), NL-1000, and the StepWatch. Young Adults (21-40 years) also wore the Fitbit One and Garmin vivofit 2. Middle-Age Adults (41-60 years) also wore the Apple Watch Serie 1, Fitbit One, Garmin vivoactive HR, Garmin vivofit 3, and the Samsung Gear Fit2. Older Adults (61-85 years) also wore the AppleWatch Series 1, Fitbit Ionic, Fitbit

Zip, Garmin vivoactive 3, PiezoRx, Polar M600, and the Samsung Gear Fit2 Pro. See **Additional file 2** for a graphical classification of wearable technologies by age groups.
