# Supplementary material for: A catalog of validity indices for step counting wearable technologies during treadmill walking: the CADENCE-adults study
Source: Int J Behav Nutr Phys Act. 2022 Sep 8;19:117. doi: 10.1186/s12966-022-01350-9 (PMC9461139; doi:10.1186/s12966-022-01350-9)
Supplement: Supplementary file 9 — Additional file 9. Figures of the effect of speed, wear location, and age on the overall precision of wearable technologies step counting ability. [file 12966_2022_1350_MOESM9_ESM.pdf]

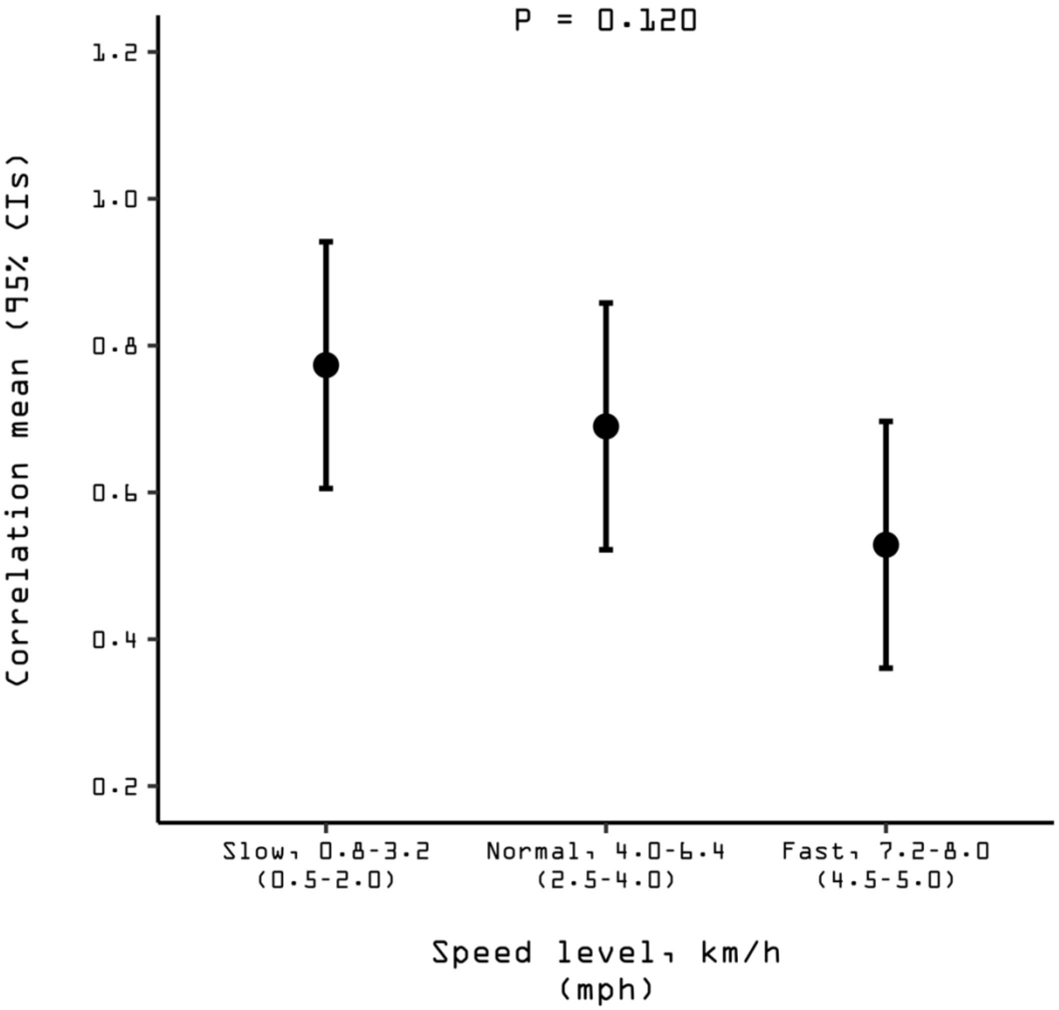

Additional file 9: Suppl Fig 1 Effect of speed on overall precision of wearable technologies' step counting ability. Each dot represents correlation coefficients averaged across wearable technologies and each specific speed level (i.e., slow, normal or fast), with corresponding 95% confidence intervals (CIs) extending above and below that point estimate. Correlation coefficients closer to 1.0 indicate tighter relationship (more precise) to directly-observed steps, i.e., increased precision. 95% CIs that do not overlap indicate significant differences.

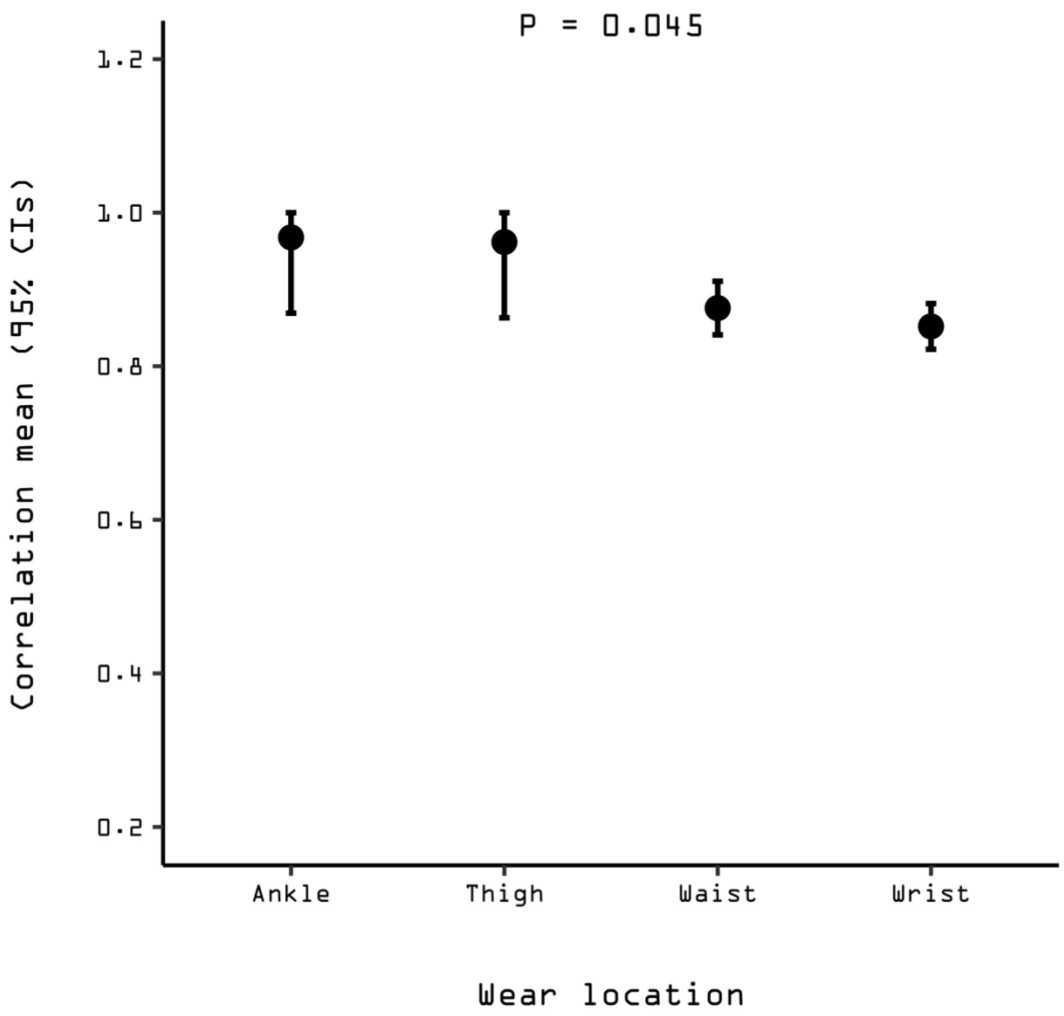

Additional file 9: Suppl Fig 2 Effect of wear location on overall precision of wearable technologies' step counting ability. Each dot represents correlation coefficients averaged across wearable technologies and each specific wear location for all walking bouts, with corresponding 95% confidence intervals (CIs) extending above and below that point estimate. Correlation coefficients closer to 1.0 indicate tighter relationship (more precise) to directly-observed steps, i.e., increased precision. 95% CIs that do not overlap indicate significant differences.

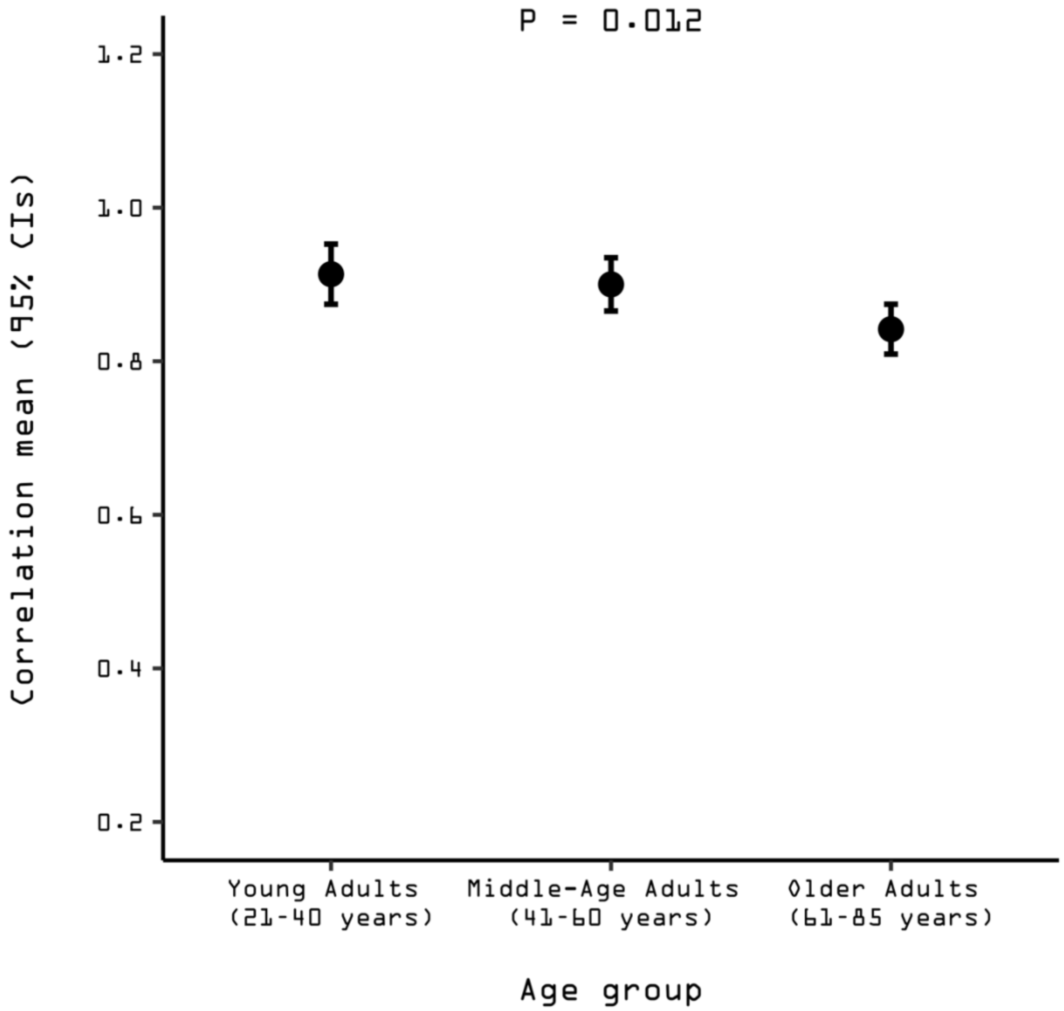

Additional file 9: Suppl Fig 3 Effect of age on overall precision of wearable technologies' step counting ability. Each dot represents correlation coefficients averaged across wearable technologies and each specific age group for all walking bouts, with corresponding 95% confidence intervals (CIs) extending above and below that point estimate. Correlation coefficients closer to 1.0 indicate tighter relationship (more precise) to directly-observed steps, i.e., increased precision. 95% CIs that do not overlap indicate significant differences.
