# Supplementary material for: A catalog of validity indices for step counting wearable technologies during treadmill walking: the CADENCE-adults study
Source: Int J Behav Nutr Phys Act. 2022 Sep 8;19:117. doi: 10.1186/s12966-022-01350-9 (PMC9461139; doi:10.1186/s12966-022-01350-9)
Supplement: Supplementary file 10 — Additional file 10. Figures of the effect of speed, wear location, and age on bias of wearable technologies step counting ability. [file 12966_2022_1350_MOESM10_ESM.pdf]

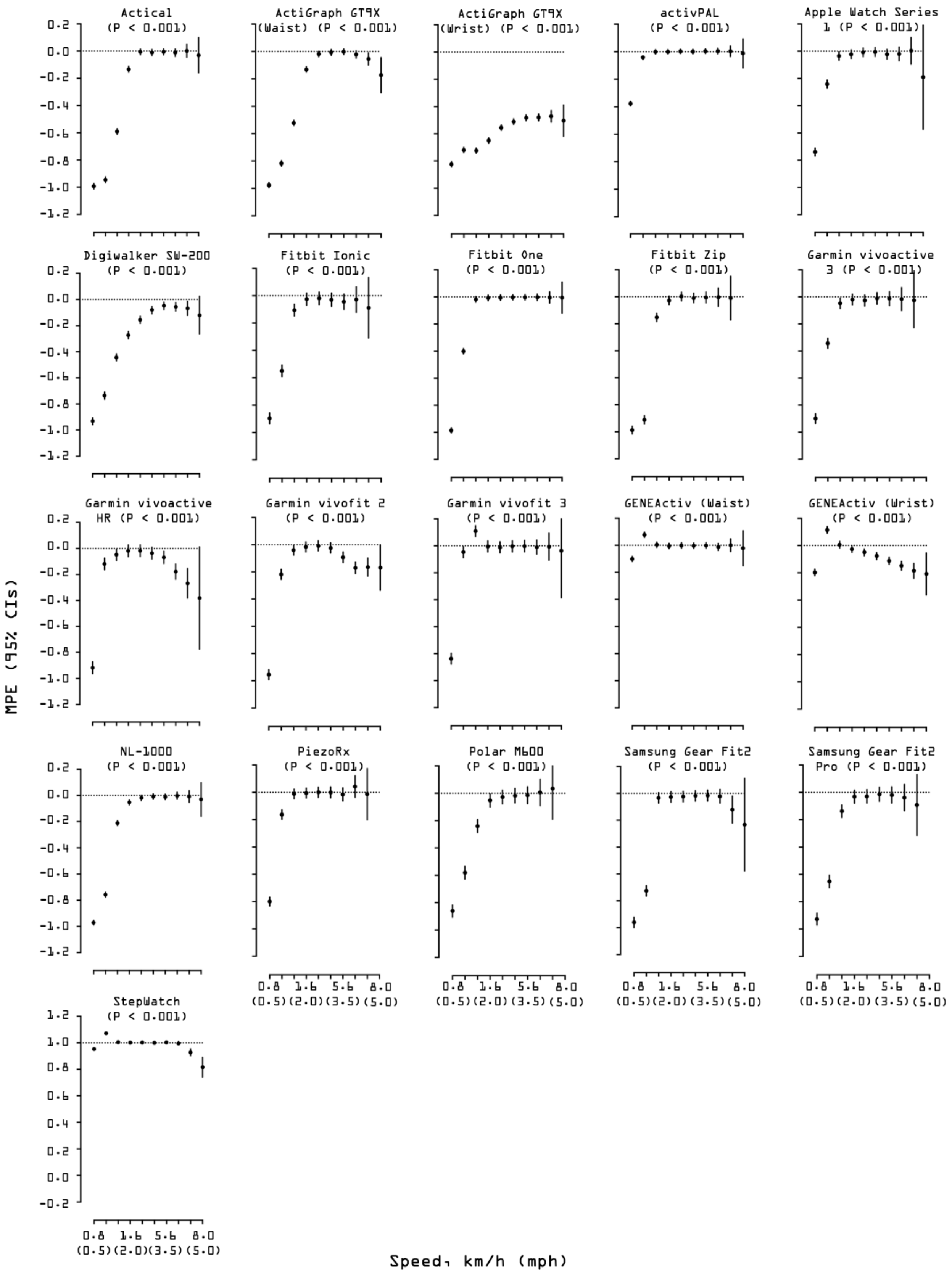
Actical (P < 0.001)
ActiGraph GT9X (Waist) (P < 0.001)
ActiGraph GT9X (Wrist) (P < 0.001)
activPAL (P < 0.001)
Apple Watch Series 1 (P < 0.001)
Digiwalker SW-200 (P < 0.001)
Fitbit Ionic (P < 0.001)
Fitbit One (P < 0.001)
Fitbit Zip (P < 0.001)
Garmin vivoactive 3 (P < 0.001)
Garmin vivoactive HR (P < 0.001)
Garmin vivofit 2 (P < 0.001)
Garmin vivofit 3 (P < 0.001)
GENEActiv (Waist) (P < 0.001)
GENEActiv (Wrist) (P < 0.001)
NL-1000 (P < 0.001)
PiezoRx (P < 0.001)
Polar M600 (P < 0.001)
Samsung Gear Fit2 (P < 0.001)
Samsung Gear Fit2 Pro (P < 0.001)
StepWatch (P < 0.001)
MPE (95% CIs)
0.2
0.0
-0.2
-0.4
-0.6
-0.8
-1.0
-1.2
1.2
1.0
0.8
0.6
0.4
0.8 1.6 5.6 8.0
(0.5) (2.0) (3.5) (5.0)
Speed, km/h (mph)

Additional file 10: Suppl Fig 1 Effect of speed on bias (mean percentage error, MPE) of wearable technology's step counting ability. MPE (%) and corresponding 95% confidence intervals (CIs; estimated using mixed effect models) respective to each technology are plotted across speed levels (slow, normal and fast). Slow speed bouts: 0.8, 1.6, 2.4, 3.2 km/h (0.5, 1.0, 1.5, 2.0 mph); normal speed bouts: 4.0, 4.8, 5.6, 6.4 km/h (2.5, 3.0, 3.5, 4.0 mph); fast speed bouts: 7.2, 8.0 km/h (4.5, 5.0 mph). Each black dot represents grouped averages of MPE values, with 95% CIs estimated using mixed effect models and extending above and below that point estimate. The 95% CIs bars are not drawn when they are shorter than the height of the symbol. MPE values closer to 0% (indicated by a dashed line) are indicative of improved bias and negative values mean undercounting. 95% CIs that do not overlap are interpreted as significantly different, while 95% CIs that overlapped with another point estimate are interpreted as not significantly different. Overall likelihood ratio test *P* value is reported for the effect of all speeds on MPE. See **Additional file 2** for a graphical classification of wearable technologies' location by age groups.

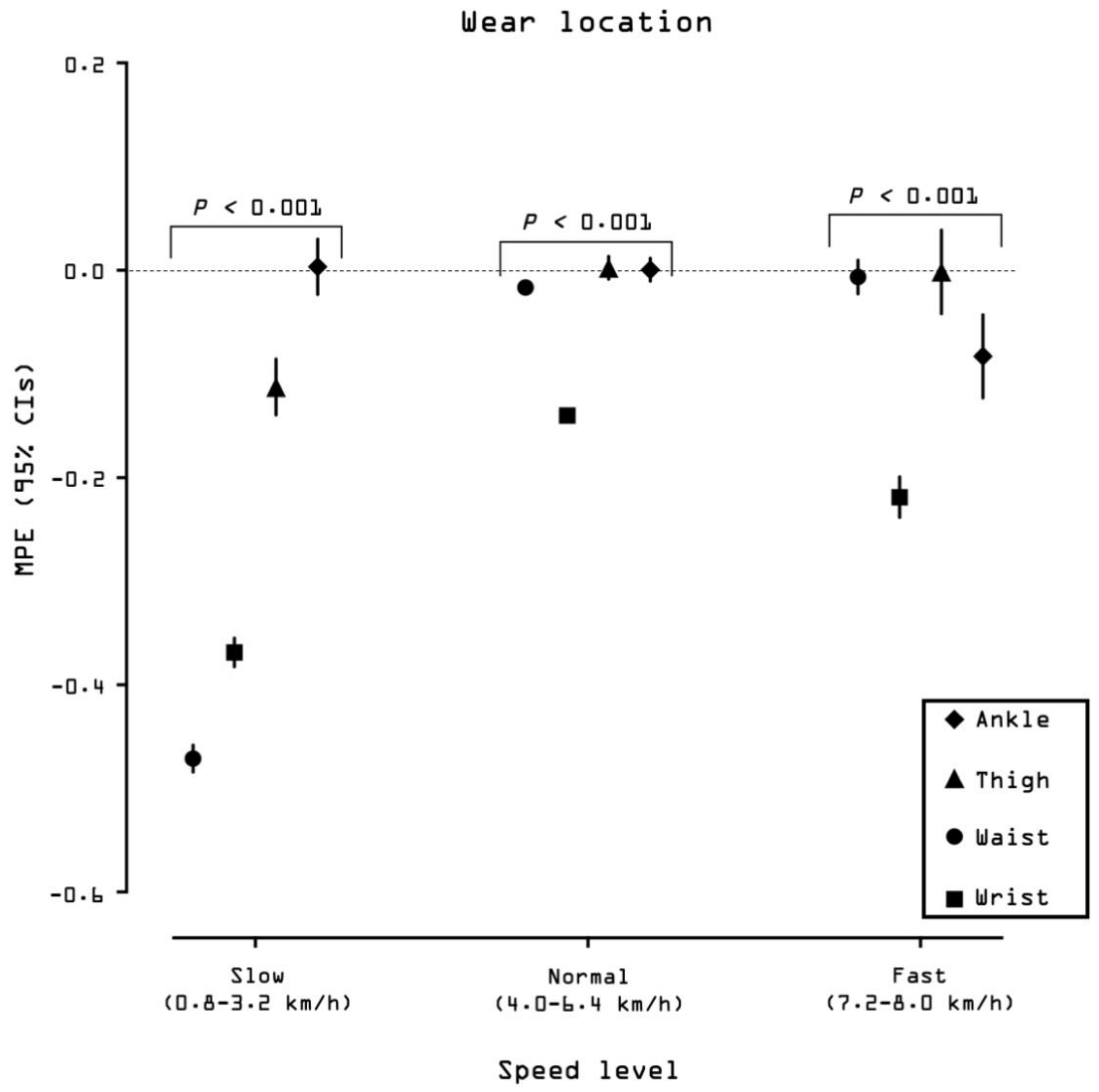

Additional file 10: Suppl Fig 2 Effect of wear location on bias (mean percentage error, MPE) of wearable technologies' step counting ability. MPE (%) and corresponding 95% confidence intervals (CIs; estimated using mixed effect models) of each wear location are presented at slow, normal, and fast walking speeds. MPE values were averaged across devices respective to each wear location for slow, normal, and fast walking speeds. MPE values closer to 0% are indicative of improved bias and negative values mean undercounting. The 95% CIs bars are not drawn when they are shorter than the height of the symbol. Further, where 95% CIs do not overlap, there are

significant differences between locations. *P* value is reported for the effect of wear location on MPE for each specific speed level. Ankle-worn wearable: StepWatch. Thigh-worn wearable: activPAL. Waist-worn wearables: Actical, ActiGraph GT9X, Digi-Walker SW-200, Fitbit One, Fitbit Zip, GENEActiv, NL-1000, PiezoRx. Wrist-worn wearables: ActiGraph GT9X, Apple Watch Serie 1, Fitbit Ionic, Garmin vivoactive 3, Garmin vivoactive HR, Garmin vivofit 2, Garmin vivofit 3, GENEActiv, Polar M600, Samsung Gear Fit2, Samsung Gear Fit2 Pro. See **Additional file 2** for a graphical classification of wearable technologies by age groups.

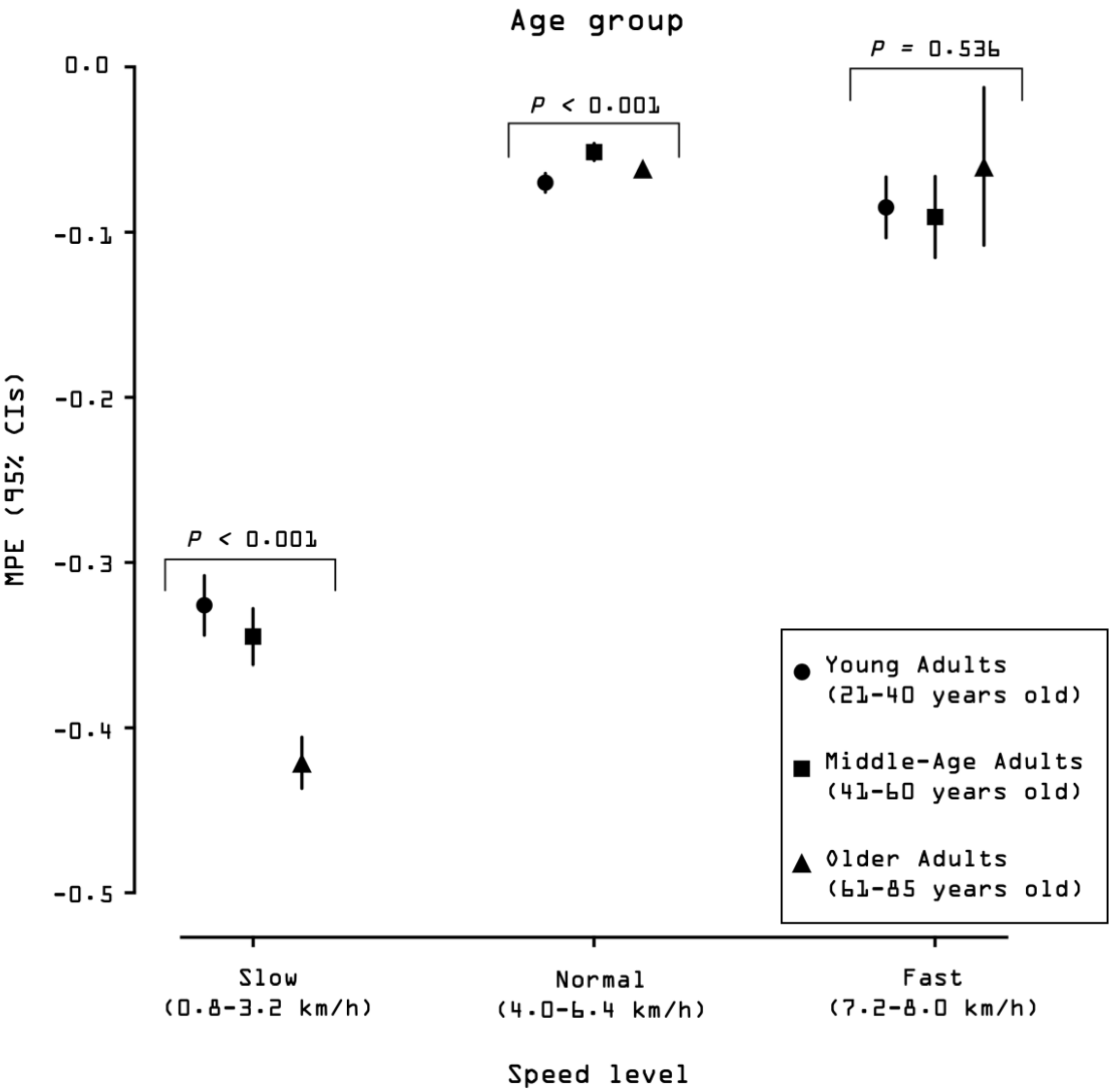

Additional file 10: Suppl Fig 3 Effect of age on bias (mean percentage error, MPE) of wearable technologies' step counting ability. MPE (%) and corresponding 95% confidence intervals (CIs; estimated using mixed effect models) of each age group are presented at slow, normal, and fast walking speeds. MPE values were averaged across devices respective to each age group for slow, normal, and fast walking speeds. MPE values closer to 0% indicate improved bias and negative values mean undercounting. The 95% CIs bars are not drawn when they are shorter than the height of the symbol. Further, where 95% CIs do not overlap, there are significant differences between

locations. Likelihood ratio test *P* value is reported for the effect of age on MPE for each specific speed level. All age groups (21-85 years) wore the Actical, ActiGraph GT9X (Waist), ActiGraph GT9X (Wrist), activPAL, Digi-Walker SW-200, GENEActiv (Waist), GENEActiv (Wrist), NL-1000, and the StepWatch. Young Adults (21-40 years) also wore the Fitbit One and Garmin vivofit 2. Middle-Age Adults (41-60 years) also wore the Apple Watch Serie 1, Fitbit One, Garmin vivoactive HR, Garmin vivofit 3, and the Samsung Gear Fit2. Older Adults (61-85 years) also wore the AppleWatch Series 1, Fitbit Ionic, Fitbit Zip, Garmin vivoactive 3, PiezoRx, Polar M600, and the Samsung Gear Fit2 Pro. See **Additional file 2** for a graphical classification of wearable technologies by age groups.
